# High-Throughput Glycomic Methods

**DOI:** 10.1021/acs.chemrev.1c01031

**Published:** 2022-07-07

**Authors:** Irena Trbojević-Akmačić, Guinevere S. M. Lageveen-Kammeijer, Bram Heijs, Tea Petrović, Helena Deriš, Manfred Wuhrer, Gordan Lauc

**Affiliations:** † Genos, Glycoscience Research Laboratory, Borongajska cesta 83H, 10 000 Zagreb, Croatia; ‡ Center for Proteomics and Metabolomics, Leiden University Medical Center, PO Box 9600, 2300 RC Leiden, The Netherlands; § Faculty of Pharmacy and Biochemistry, University of Zagreb, A. Kovačića 1, 10 000 Zagreb, Croatia

## Abstract

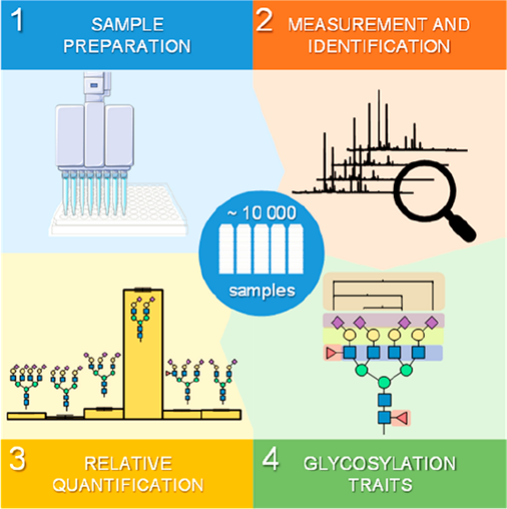

Glycomics aims to identify the structure and function of the glycome, the complete set of oligosaccharides (glycans), produced in a given cell or organism, as well as to identify genes and other factors that govern glycosylation. This challenging endeavor requires highly robust, sensitive, and potentially automatable analytical technologies for the analysis of hundreds or thousands of glycomes in a timely manner (termed high-throughput glycomics). This review provides a historic overview as well as highlights recent developments and challenges of glycomic profiling by the most prominent high-throughput glycomic approaches, with *N*-glycosylation analysis as the focal point. It describes the current state-of-the-art regarding levels of characterization and most widely used technologies, selected applications of high-throughput glycomics in deciphering glycosylation process in healthy and disease states, as well as future perspectives.

## 1 Introduction

Glycosylation is the most common and complex post-translational protein modification. In addition to proteins, many lipids are glycosylated, and just recently it was shown that elaborated glycan structures can also be attached to RNA.^1^ In addition, the field of glycomics comprises polysaccharides such as glycosaminoglycans (GAGs). Regarding protein glycosylation, the attachment of different glycans to the same glycosylation site (alternative glycosylation or microheterogeneity) greatly contributes to the structural variability of these molecules and influences their function in a way that is analogous to the effects of changes in protein sequence caused by mutations in the corresponding gene.^2^

The most intensively studied example of the importance of alternative protein glycosylation for biological functions are immunoglobulins, which are among the main weapons in our arsenal for the multifacetted war against pathogens. They are an elaborate tool that can specifically recognize foreign structures. However, the binding to an antigen is only one aspect of their function. Namely, immunoglobulins have to activate proper molecular mechanisms to “deal with” this foreign, nonself object. The choice of how to react to a foreign antigen is one of the most complex decisions that has to be made, and these choices have to be made continuously throughout our lifetime. Alternation of immunoglobulin G (IgG) glycosylation appears to be a check point for initiation of specific effector functions directing immunoglobulins to different receptors, and in this way activating different branches of our immune system.^3,4^ Glycans attached to the fragment crystallizable (Fc) region are an integral part of the constant region domain (C_H_2) of antibodies, and as such represent an integral structural component that participates in the interaction with Fc receptors and other proteins. Attaching a different glycan to the polypeptide backbone changes the structure of the antibody and modifies its affinity for different receptors. The best currently known example is the role of core-fucose that acts as a “safety-switch” against antibody-dependent cellular cytotoxicity (ADCC) by attenuating binding of IgG to Fc-γ-receptor IIIA.^5^ Other effector functions modulated by IgG Fc glycosylation include antibody-dependent phagocytosis and complement activation with an often complex dependency of effector function on various glycosylation features.^6,7^ Glycosylation is an essential element in the development of different therapeutic monoclonal antibodies (mAbs), and glycoengineered drugs are already on the market.^8^ Interindividual differences in glycosylation are large and may be an important underlying element for the response or nonresponse to a given drug, ABO blood groups are a good example, but for the vast majority of drugs, data is still missing and this field needs further exploration.

Contrary to the polypeptide sequences of a protein that are predominantly changed by inducing changes in the sequence of the corresponding genes, glycans are encoded in a complex network of at least several dozen genes that are (beside allelic variants) also affected by epigenetics and the environment.^9^ This enables flexible and dynamic regulation of protein function and is extensively used to fine-tune functions of many proteins. More than 30 years ago, the initial discovery of changes in the IgG glycome composition in diseases was made,^10^ and until now, over 150 000 different glycomes have been analyzed in different diseases and physiological states (see section 6, *Applications*). Changes in glycosylation associate with numerous diseases, often even before any other symptoms of the disease are detectable, indicating that they might be a part of the molecular pathophysiology leading to the disease.^11^ With aging, the IgG *N*-glycome converts from a composition that is suppressing inflammation to an inflammation-promoting *N*-glycome that seems to be an underlying risk factor in many cardiometabolic and inflammatory diseases.^4,12,13^

The importance and critical role of carbohydrate post-translational modifications on many cell surfaces and secreted proteins’ structure as well as function have been studied for several decades. The majority of both high-throughput (HT) and functional glycomic studies of individual glycoproteins were performed on IgG, thus this is by far the most studied glycoprotein. Nevertheless, there’s still much more we don’t know about glycosylation, than what we do know. HT methods for the analysis of other proteins have been developed only recently, thus our level of understanding of the importance of interindividual difference in protein glycosylation is very low. Nevertheless, because glycosylation is an integral part of the proper functioning of every organism in nature the development of effective tools for detecting carbohydrate structures, and their changes under various physiological and pathological conditions is of utmost importance.^14^

This review provides a historical background of the field of glycomics with a focus on recent developments in methodology and applications of HT glycomics (roughly defined as the glycan analysis of thousand(s) of samples in large-scale studies). For the purpose of this review, we will define medium-throughput glycomics as analysis of hundred(s) of samples and low-throughput as methodology applicable to glycan analysis in small-scale studies (less than one hundred samples) taking into account the whole process from sample preparation to data analysis. Because of the complexity of glycan analysis workflows, analytical limitations of any of the steps ranging from study design via sample preparation, measurement, data processing, and data analysis may limit the advancement from low- to high-throughput applications. Therefore, significant methodological and technological efforts that are not necessarily at a HT level for the moment have also been highlighted with a promise of future implementation in HT glycomics workflows.

## 2 Levels of Characterization

**Figure 1:**
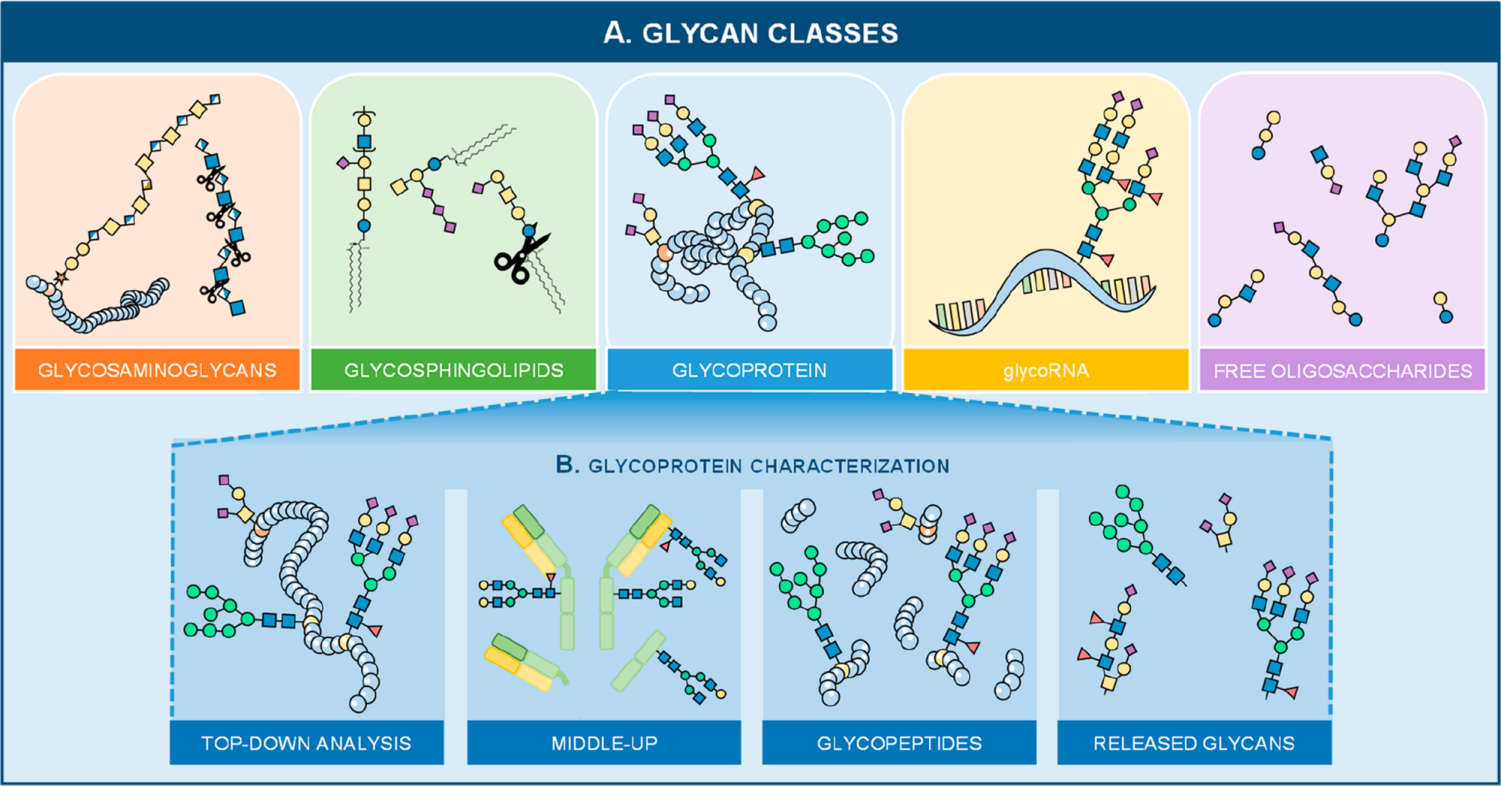
(A) Various glycan classes exist such as glycosaminoglycans (GAGs), glycosphingolipids (GSLs), glycoRNA, free oligosaccharides, and glycoproteins. (B) A distinction can be made in various characterization categories of the latter glycan type. Namely, glycoproteins can be analyzed intact using a top-down method or a middle-up approach that can be used to study the subunits of (monoclonal) antibodies. Other methods include enzymatic digestions, which either cleave the protein into (glyco)peptides, also known as the bottom-up approach, or cleave the glycan portion from its conjugate (released glycan analysis). The latter two are currently the only characterization approaches that can be processed and analyzed in a HT manner. Several enzymes are available for GAGs which will result in disaccharides (indicated by the scissors), while enzymes available for GSL analysis will release the glycan headgroup from the lipid portion (indicated by the scissors).

Glycosylation occurs on a diverse range of biomolecules, resulting in glycoproteins, proteoglycans, as well as glycosphingolipids (GSLs). Next to these glycoconjugates, other glycan classes exist such as free oligosaccharides, GAGs, and the just recently discovered glycosylated RNA^1^ (Figure 1A). Significant progress in analytical approaches for their analysis has been achieved in recent years (especially in the case of free oligosaccharides in, e.g., human milk.^15−17^ While all glycan classes are mutually important, the only glycan class that is currently analyzed in HT large-scale studies are the glycoproteins (Figure 1B) and will be the main focus of the next sections.

**Figure 2:**
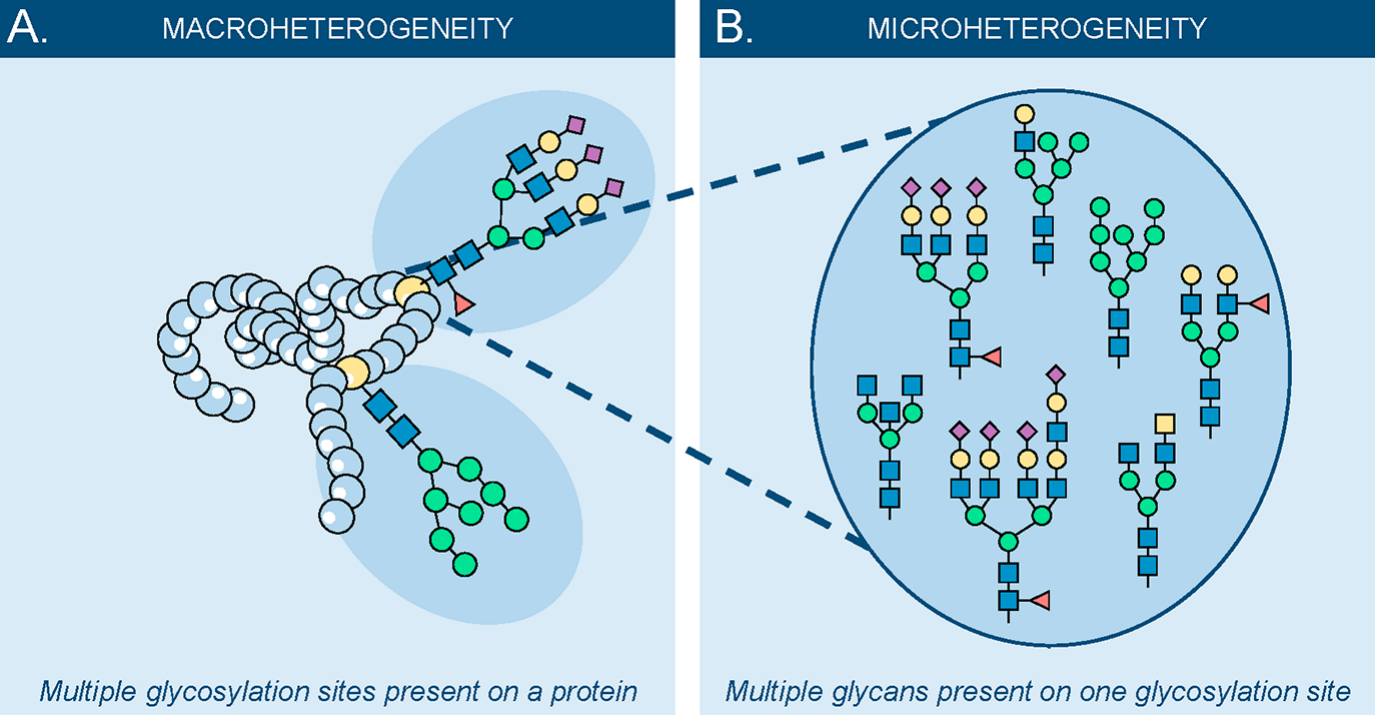
Macro- versus microheterogeneity of a glycoprotein. (A) Macroheterogeneity is the diversity of (multiple) glycosylation sites on a single glycoprotein. (B) Microheterogeneity is the variety on a single glycosylation site, where various glycan structures can be found.

Glycoproteins can be further distinguished into those containing *N*-linked glycosylation (attachment through a nitrogen atom) and *O*-linked glycosylation (attachment through an oxygen atom). *N*-Linked glycosylation occurs only within a specific amino acid sequence (asparagine–X–serine or threonine (Asn–Xxx–Ser/Thr or N–X–S/T)). In this consensus sequence, X can be any of the amino acids except for proline (Pro or P). Unlike *N*-linked glycosylation, there is no clear amino acid sequence for *O*-linked glycosylation except that the glycan is attached to a Ser (S) or Thr (T). A glycoprotein can have several glycosylation sites (macroheterogeneity; Figure 2A) and various glycan species on a single site (microheterogeneity; Figure 2B). Whether or not the glycosylation site of a protein is fully occupied, partially occupied, or unoccupied depends on the conformation of the protein, spatial and temporal availability of glycosyltransferases, their activity, transcription factors, as well as the availability of sugar precursors.

The level of characterization can be selected dependent on the research question, varying from the intact analysis of a purified glycoprotein up to the analysis of the total released glycome of complex biological matrices (Figure 1B). The intact analysis allows, next to its glycosylation, to study the presence of other (post-translational) modifications, also known as proteoforms. While this provides insightful information about the protein it also requires pure substances, high sensitivity, high-resolution mass spectrometry (MS) analysis, and often additional separation techniques are applied. Moreover, the data analysis can be rather complicated as multiple modifications should be taken into account. In the case of biopharmaceuticals, the data analysis can be simplified by using a middle-up or middle-down approach. For this purpose, specific enzymes are available that perform a proteolytic cleavage in the hinge regions of the IgG resulting in specific Fc and antigen-binding fragment (Fab) domain. Dependent on the enzyme and/or additional reduction steps, individual subunits (Fc/2 or Fab/2) can be analyzed or the complete Fc2 and Fab2 domain can be investigated. This approach allows getting insights into the macroheterogeneity of the glycoprotein. However, it becomes complicated in the case of a large macroheterogeneity, and often it remains difficult to define glycoprotein microheterogeneity. HT glycomic studies on an intact level are still sparse on large sample sets (e.g., one of the largest intact HT glycomic studies was performed using 96 serum samples)^18^ and are more applied to glycopeptide and released glycan approaches.

The identification and characterization of glycopeptides can be established through a bottom-up approach. This method allows confirmation of the protein identity when a glycopeptide holds a unique peptide backbone as well as the occupancy of a specific glycosylation site. But also here, in the case of a large microheterogeneity, the complexity of the data can be a bottleneck, slowing down the throughput. By applying tandem MS, more information can be obtained about the structural composition of the glycan and the peptide. For HT analysis, the complexity of the sample needs to be condensed by efficient sample preparation steps such as affinity enrichment steps. Excellent examples for HT workflows are those for IgG, immunoglobulin A (IgA), and α-1-acid glycoprotein (AGP) enriched from serum or plasma^19−21^ or that of prostate-specific antigen (PSA) from seminal fluid,^22^ all allowing the complete sample preparation step in a 96-well plate format.

**Figure 3:**
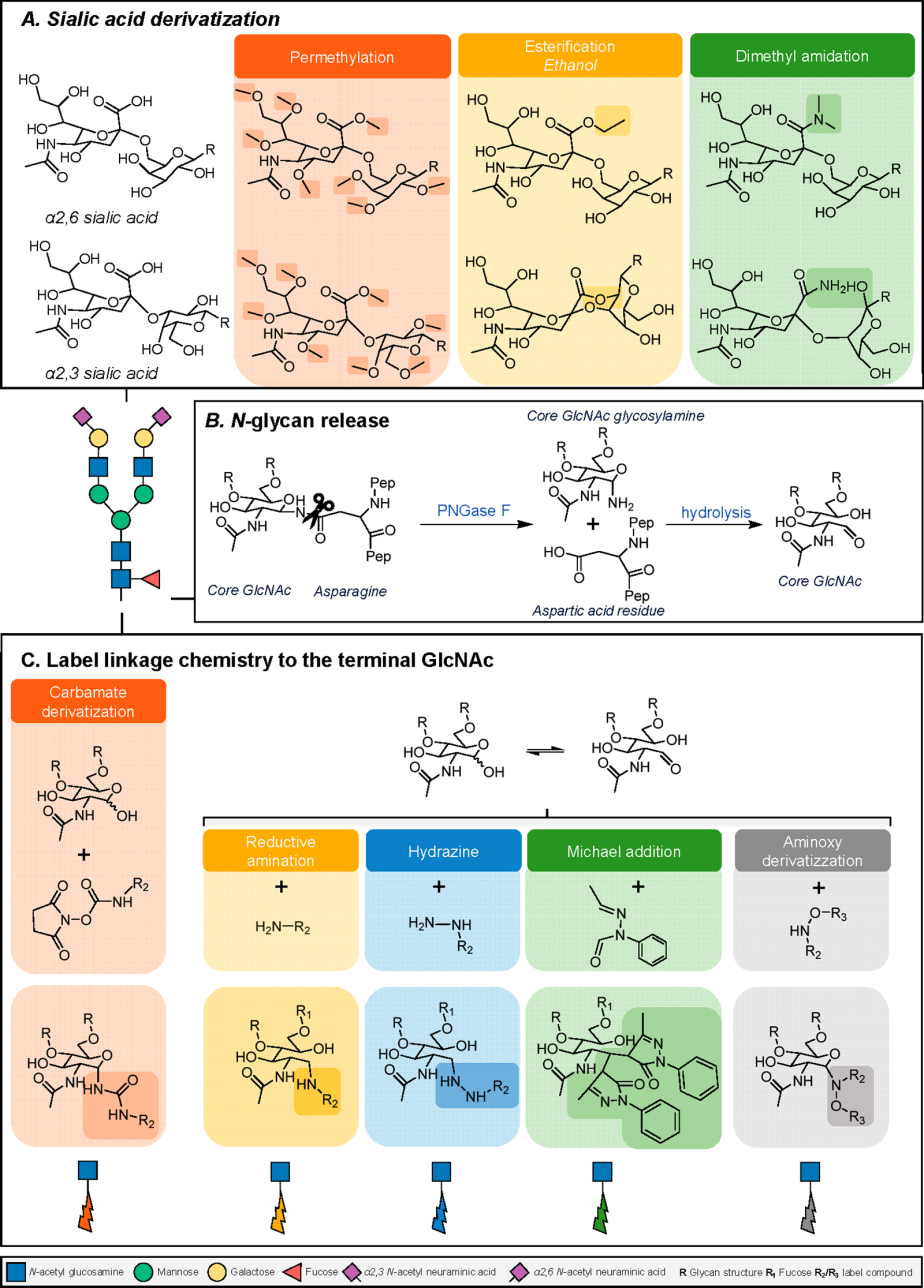
A diversity of different chemistries is available to enable glycan analysis. (A) The most common derivatization strategies applied on terminal sialic acids, enabling stabilization and, in regard to esterification or dimethylamidation, also identification of the different isomers based upon mass difference by MS. In regard to the dimethylamidation procedure, the reaction consists of two parts. In the first step, α2,3-linked sialic acids react with the adjacent galactose to form a lactone, and the α2,6-linked sialic acids form a stable dimethylamide. The second step involves the addition of ammonia, with the lactone undergoing aminolysis, thereby transforming the carboxylic acid into a stable amide.^26^ (B) Illustration how an *N*-glycan attached to a protein (via an asparagine) is cleaved using the enzyme PNGase F. (C) Common procedures that are performed at the reducing end of the glycan: fluorescence detection can be enabled by introducing a label with a fluorophore, or MS ionization can be improved by adding a permanent positive charge (e.g., hydrazide labeling) or introducing a tertiary amine (e.g., carbamate chemistry), which could also allow the simultaneous analysis of glycans from different samples through the incorporation of stable isotopes (e.g., TMT-labeling).

Released glycan analysis allows the analysis of the total glycomic profile of complex biomatrices by releasing all the glycans from the glycoproteins present in the sample. This is in great contrast to that of the intact, middle-down, and bottom-up approach where a rather pure sample is required prior to MS analysis. For the analysis of *N*-linked glycans, the enzyme peptide-*N*-glycosidase F (PNGaseF) is often utilized as it is able to release all *N*-linked glycans with a free reducing end from proteins (except when a core-fucose is present in α3-linkage) (Figure 3). More challenging is the release of *O*-linked glycans, as there is no universal enzyme available that can cleave all *O*-glycans, therefore, reductive β-elimination, a chemical approach, is often applied. Just recently, a workflow was published that allows the sequential release of *N*- and *O*-glycan in a 96-well format for approximately 500 000 cells using a PVDF membrane.^23^ However, this approach has not yet been performed on a larger sample set, which is mainly due to the time-consuming (manual) data interpretation and is therefore not yet to be considered a HT approach. Of note, this approach has been further optimized by de Haan et al.^24^ and employs additional 2-aminobenzamide (2-AB) labeling step, facilitating *O*-glycan isomer separation and enhancing sensitivity of detection.

In contrast, various workflows are developed to target solely the *N*-glycans, and most HT glycomic platforms result in two-dimensional (2D) data (intensity versus time or *m*/*z*), which can be used for the characterization and detection of the *N*-glycome. Next to the fact that the 2D format makes it easier to perform data analysis, established platforms with sizable glycan databases or repositories can be used (Table 1). Several extensive studies have been performed by using a separation platform coupled to a fluorescent detector (FLD) or by direct analysis using MS. However, it should be noted that all of these analytical platforms require extra sample preparation steps to enable their detection. For example, glycans themselves do not contain a fluorophore, which makes it impossible to detect them at high sensitivity by fluorescence. Therefore, the reducing end is often chemically modified by adding a label with fluorescent properties (Figure 3). For the detection by MS, it should be taken into account that sialic acids tend to be rather unstable in positive ionization mode when a time-of-flight (TOF) analyzer is being used. To avoid this, a derivatization approach is applied (Figure 3) and dependent on the chemistry a distinction can be made between the differently linked sialic acids as a mass difference is introduced (esterification or dimethylamidation).^25^

## 3 Experimental Design of High-Throughput Glycomics Study

HT glycomics results in large amounts of data being generated during a course of usually several weeks or months and requires several steps of processing. Samples are analyzed in 96-sample (or 384-sample) batches which can be affected by experimental conditions, e.g., different batches of reagents, variable laboratory conditions, multiple analysts as well as different instruments. Moreover, glycosylation is confounded by different biological variables that have to be accounted for during the analysis. Proper experimental design is therefore of utmost importance to ensure the quality of generated data.

Human protein glycosylation is shown to be vastly variable between individuals of different ages, sex, body mass index (BMI), smoking habits, medication use, pregnancy, and inflammatory status, as well as a geographical origin.^27−33^ These variables are important confounders that should be taken into account in the design of a study, making sure they are equally distributed between studied biological groups (e.g., controls and patients, different therapy groups, etc). Moreover, although glycans are generally stable at common sample storage conditions, the differential sample preprocessing steps (especially for complex biological samples like plasma/serum, tissue samples, etc.) might have an impact on the obtained results due to loss/aggregation of specific glycoproteins if the analysis is done on the level of released glycans. Therefore, sample preprocessing, as well as storage information and location of the sample collection, should also be considered.

Proper experimental design of HT glycomics studies takes into account known biological confounders during blocked randomization and ensures that technical replicates of a representative standard sample are included in each batch of samples. Through blocked randomization, all known biological and experimental confounders should be equally distributed between each batch (block) of samples. In that case, any observed systematic shifts of individual batches most likely originate from experimental and not biological variation. Replication of a standard sample goes hand-in-hand with the blocked design of a study because it allows for the detection of potential batch effects (systematic shifts in measured data originating from the same 96- or 384-well plate) and any other technical variations caused by experimental conditions. When the abovementioned conditions in experimental design are met, it is possible to perform statistical batch correction which can somewhat aid in obtaining better quality data that would otherwise be skewed due to batch effects. Standard sample replicates also enable quality control of overall method variability during cohort analysis.

In addition to standard sample replicates added at the beginning of a study and processed together with the samples, it is common to add another set of standards (system suitability standards) during sample measurement. These depend on the technology being used for the glycan analysis, e.g., fluorescently labeled released *N*-glycans in ultrahigh-performance liquid chromatography (UHPLC) and capillary gel electrophoresis (CGE) analysis and are analyzed in parallel to cohort samples during every data acquisition sample set on each instrument used for the cohort analysis. This ensures that the instruments used in the study are working within the desired specifications and allow detection of any significant impact that deteriorating instrument components (e.g., UHPLC column, CGE polymer) might have on the analysis.

**Table 1:** Most Common Databases, Repositories and Software Tools for Glycomic Studies^a^

database	description	URL
**Carbohydrate Databases**		
Carbohydrate Structure Database (CSDB)	manually curated natural carbohydrate structures, taxonomy, bibliography, NMR, and other data from the literature (up to 2019).	http://csdb.glycoscience.ru/
GAG-DB	a database that contains 3D structures of GAG binding proteins	https://gagdb.glycopedia.eu/
GlycoBase (now GlycoStore)	over 650 *N*- and *O*-linked glycan structures available (exoglycosidase sequencing, U(H)PLC, and MS (MALDI-MS, LC-MS/MS) data	http://www.glycostore.org
GlycomeDB	contains glycan structures but has now been implemented in GlyTouCan	http://www.glycome-db.org/
GlyConnect	contains glycan structures and their association with proteins, glycopeptide and glycosylation sties (curated); GlyConnect is integrated with GlyGen	https://glyconnect.expasy.org/
Glycosciences.DB	a web portal that contains glycoinformatic databases and tools with a specific focus on 3D structures and 3D models; contains over 27 000 glycan entries, 13 900 3D structures, and almost 3500 NMR spectra	http://www.glycosciences.de/
GlycoStore	a curated chromatographic, electrophoretic, and mass spectrometry composition database of *N*-, *O*-, and GSL glycans and free oligosaccharides associated with a range of glycoproteins, glycolipids, and biotherapeutics; approximately 850 unique glycan structure entries supported by over 10 000 retention positions determined (HILIC-FLD, U(H)PLC-FLD, PGC-LC-MS, and CGE-LIF).	https://www.glycostore.org/
GlyGen	contains glycan structures as well as their association with proteins	https://www.glygen.org/
GlyTouCan	a repository where more than 120 000 glycan structures are registered (uncurated); each glycan structure is assigned with a unique accession number	https://glytoucan.org/
GlyXbase	database that contains the GU values of more than 400 glycan structures (glyXera)	Goldberg et al.^34^
GUdatabase	library of APTS labeled *N*-glycans analyzed by C100 HT multicapillary electrophoresis system	https://lendulet.uni-pannon.hu/index.php/tools/2-uncategorised/46-c100htdatabase
KEGG	contains glycan structures present in KEGG pathways	http://www.genome.jp/ligand/kcam/
Lipopolysaccharide	database for glycolipid structures (updated until 2007)	http://lipidbank.jp/cgi-bin/main.cgi?id=CLS
UniCarb-DB	a database and repository for glycomic MS data. Over 1100 structures and 1550 MS spectra are provided.	https://unicarb-db.expasy.org/
		
**Glycan Binding Proteins**		
Glycan Array Dashboard (GLAD)	automatically analyzes and visualizes glycan array data using glycan-binding intensities as input	https://glycotoolkit.com/GLAD/
Glydin’	visualizes and map the similarity of glycoepitopes in an interactive network	https://glycoproteome.expasy.org/epitopes/
GlycoEpitopeDB	a database of antibodies that recognizes carbohydrates and glyco-epitopes (curated)	https://www.glycoepitope.jp/epitopes
MatrixDB	database that is focused on the interaction by ECM proteins, proteoglycan and GAGs (curated)	http://matrixdb.univ-lyon1.fr/
SugarBindDB	database that provides information on carbohydrate sequences to which pathogenic organisms specifically adhere (curated)	https://sugarbind.expasy.org/
		
**Glycan Processing Pathways and Enzymes**		
Carbohydrate-Active enZYmes Database (CAZy)	a database that holds information about carbohydrate-active enzymes	http://www.cazy.org/
GlycoGene DataBase (GGdb)	a database that provides information about which genes are associated with the biosynthetic pathway of glycans (e.g., glycosyltransferases, sugar nucleotide synthases, sugar-nucleotide transporters, sulfotransferases); over 180 human glycogenes are identified, cloned, and characterized	https://acgg.asia/ggdb2/
Glycologue	prediction of glycosyltransferases and enzymes involved in the biosynthetic pathway of *O*- and *N*-glycans, human milk oligosaccharides (HMOs) as well as gangliosides	https://glycologue.org/
SphinGOMAP	provides a pathway map for (glyco)sphingolipid biosynthesis	http://sphingolab.biology.gatech.edu/
		
**Software Tools**		
AutoGU	automated annotation and quantification of glycans using the GU index (HPLC-FLD data)	https://academic.oup.com/bioinformatics/article/24/9/1214/206953
AutoGUI	automated annotation and quantification of glycans using the GU index (LC-MS data)	https://github.com/ruizhang84/GlycanGUIApp
Byonic	commercial tool for automated identification of glycopeptides using MS/MS data	https://www.proteinmetrics.com/products/byonic/
Cartoonist	annotation of MS peaks with *N*-glycan cartoons	available at request to the authors^35^
GlycanAnalysis	annotation of MS/MS spectra using glycan structures from GlycomeDB and KEGG glycan	https://www.shimadzu.co.jp/mass-research/soft.html
GlycanAnalyzer	automatically interprets exoglycosidase array by pattern matching of peak shifts of *N*-glycans after exoglycosidase digestion	https://glycananalyzer.neb.com
GlycoDigest	a software tool that simulates the exoglycosidase digestion (based upon GlycoBase)	https://glycoproteome.expasy.org/glycodigest/
GlyConnect Compozitor	visualizes a set of glycan compositions and creates a network based upon shared monosaccharides	https://glyconnect.expasy.org/compozitor/
GlycoForest	software tool that uses a partial de novo algorithm for sequencing glycan structures based on MS/MS spectra	https://glycoforest.expasy.org/
GlycanMass	assists in the calculation of glycans masses (free reducing end, permethylated, or peracetylated) in Daltons	https://web.expasy.org/glycanmass/
Glyco@Expasy	provides an overview of web-based glycoinformatic resources (portals, tools, and databases)	https://glycoproteome.expasy.org/glycomicsexpasy/
GlycoMod	prediction of possible glycan compositions on proteins based upon mass (free, derivatized glycans and glycopeptides)	https://web.expasy.org/glycomod/
Glycologue	a simulator of the enzymes involved in the biosynthesis of HMOs	https://glycologue.org/m/
GlycoPAT	automated identification of glycopeptides of MS/MS data	https://virtualglycome.org/glycopat/
GlycoPeptideGraphMS	automated identification of glycopeptides LC- and CE-MS data based upon known elution/migration criteria; at least one glycopeptide (node) should be assigned in the data using MS/MS.	https://bitbucket.org/glycoaddict/glycopeptidegraphms/src/master/
GlycoReSoft	automated identification of glycopeptides of MS/MS data	https://github.com/mobiusklein/glycresoft
GlycoWorkbench	tool to assist in the interpretation of glycomic MS data	https://code.google.com/archive/p/glycoworkbench/
Glynsight	visualizes and enables an interactive comparison of *N*- and *O*-glycan expression profiles	https://glycoproteome.expasy.org/glynsight/
glyXtool^MS^	automated identification of glycopeptides using MS/MS data	https://github.com/glyXera/glyXtoolMS
glyXtool^CE^	commercial tool for the automatic annotation of CE-LIF data	https://www.separations.eu.tosohbioscience.com/OpenPDF.aspx?path=~/File%20Library/TBG/Products%20Download/Application%20Note/a21l02a.pdf
GRITS	assists in the interpretation of glycomics MS data	http://www.grits-toolbox.org/
GUcal	integrated approach with GlycoStore for the analysis of *N*-glycans by CE-LIF	https://lendulet.uni-pannon.hu/index.php/tools
HappyTools	automatic annotation of LC- and CE fluorescence data	https://github.com/Tarskin/HappyTools
LaCyTools	automatic annotation of LC- and CE-MS data	https://github.com/Tarskin/LaCyTools
Mass Spectrometry-Based Automated Glycopeptide Identification Platform (MAGIC)	automatic annotation of glycopeptides using CID spectra	https://ms.iis.sinica.edu.tw/COmics/Software_MAGIC.html
MassyTools	automatic annotation of MS data	https://github.com/Tarskin/MassyTools
O-pair (MetaMorpheus)	automated identification of *O*-glycopeptides of MS/MS data	https://github.com/smith-chem-wisc/MetaMorpheus
Peptoonist	annotation of MS peaks with *N*-glycan cartoons including the peptide backbone	https://pubs.acs.org/doi/10.1021/pr070239f
PepSweetner	assists in the manual annotation of intact glycopeptide spectra, matching the molecular mass of the precursor mass to theoretical human *N*-glycopeptides	https://glycoproteome.expasy.org/pepsweetener/app/
pGlyco	annotation of *N*-glycopeptides	https://github.com/pFindStudio/pGlyco3/releases
SimGlycan	commercial tool for the annotation of MS data using an internal database	http://www.premierbiosoft.com/glycan/
Skyline	assists in the annotation and identification of glycan and glycopeptides using LC- or CE-MS data	https://skyline.ms/
SwissMassAbacus	assists in the calculation of glycan and glycopeptide masses in Daltons	https://glycoproteome.expasy.org/swiss-mass-abacus/
UNIFI	commercial tool for automatic assignment of glycans based upon GU values (LC) and m/z value (MS)	https://www.waters.com/nextgen/us/en.html

a NMR, nuclear magnetic resonance; 3D, three-dimensional; MALDI, matrix assisted laser desorption/ionization; LC, liquid chromatography; HILIC, hydrophilic-interaction liquid chromatography; PGC, porous graphitic carbon; LIF, laser-induced fluorescence detection; GU, glucose unit; APTS, 8-amino-1,3,6-pyrenetrisulfonic acid trisodium salt; ECM, extracellular matrix; HPLC, high-performance liquid chromatography.

## 4 Historical Overview

Although a variety of analytical methods already exist for small-scale glycan studies, the demand for HT approaches that allow large-scale glycoprofiling continues to develop. These large-scale studies include from several hundred to several thousand samples of patients as well as healthy individuals. Methods used in such immense studies must possess the capacity to precisely analyze the glycome of many samples in a reasonable time frame and at a reasonable cost.

**Figure 4:**
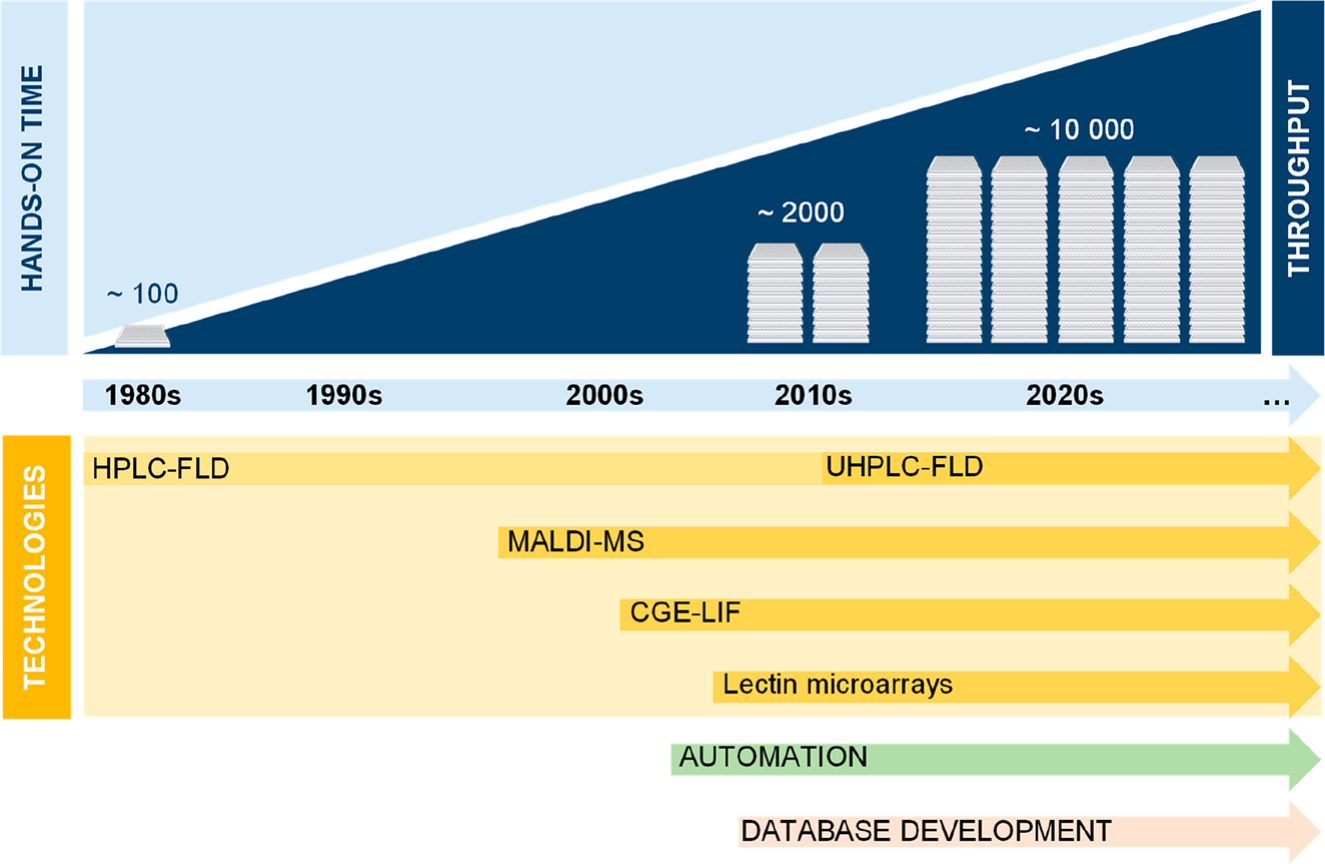
Historical overview of HT glycomic technologies applied for released *N*-glycan analysis.

The development of HPLC, as one of the main techniques for glycan separation and detection, and a forerunner technique of UHPLC, began in the 1980s for small-scale studies, using laborious and time-consuming sample preparation protocols (Figure 4).^36^ These studies set the groundwork for the development of HT glycomics. Seminal work by Mullinax et al. in 1976,^37^ Parekh et al. in 1985^10^ and 1988,^38^ and Ashford et al. in 1987^10,39^, analyzing between 50 and 150 samples have set the stage for future HT applications. Glycans were released by hydrazinolysis developed by Takasaki et al. in 1982.^40^ For labeling of released glycans, the radioactive isotope NaB_3_H_4_ was used as described in 1974 by Takasaki and Kobata.^41^ Nowadays, a combination of these two sample preparation approaches is practically obsolete for HT analyses, as novel approaches are now available that avoid the use of radioactive compounds and come with less demanding sample preparation steps. Glycans were fractioned by Bio-Gel P-4 (400 mesh) gel filtration chromatography to separate neutral glycans based on their effective hydrodynamic volumes and coupled to HPLC radioactivity flow monitor to collect analogue signals.

Hase et al., first introduced fluorescent tagging of free oligosaccharides by reductive amination back in 1979,^42^ and glycans derivatized in such a way were analyzed by 2D paper electrophoresis. The sensitivity of fluorescent labeling was further improved by using HPLC with an FLD in 1981,^43^ and workflow for fluorescent labeling and reversed-phase (RP-)HPLC-FLD analysis of *N*-glycans was further optimized by Hase et al. in 1986,^44^ enabling glycan structure estimation from only several hundreds pmol of glycans. Glycans were released by hydrazinolysis and after subsequent *N*-acetylation, the reducing ends of glycans were coupled with 2-aminopyridine (2-PA) with sodium cyanoborohydride (NaBH_3_CN) as a reducing agent. The reaction of reductive amination was performed at 90 °C for 15 h, after which the obtained fluorescent derivatives were purified by Bio-Gel P-2 column chromatography and separated using RP-HPLC. In 1987, Takahashi et al., published a comparative structural study of the neutral *N*-linked glycans of human normal polyclonal IgG and pathological IgG1 isolated from the sera of patients with multiple myeloma.^45^ After desialylation using neuraminidase as well as pepsin digestion, IgG glycopeptides were digested with *N*-oligosaccharide glycopeptidase from almonds. The reducing ends of the obtained *N*-glycan chains were reductively aminated with the fluorescent reagent 2-PA and purified by gel filtration. The mixture of *N*-glycans was separated by RP-HPLC into 15 peaks. This study is one of the first comparative studies of IgG *N*-glycosylation employing today’s most widely used general workflow for LC analysis of released *N*-glycans consisting of enzymatic digestion, fluorescent labeling, cleanup, and analysis. In conjunction with hydrazinolysis, in most of the previously mentioned studies, labeled glycans were subjected to exoglycosidase (e.g., neuraminidase) treatment prior to oligosaccharide analysis, so that only particular glycosylation traits such as galactosylation were analyzed.

Glycan release by hydrazinolysis has several significant flaws, including the requirement of using anhydrous hydrazine, which is an extremely hazardous and explosive substance. In 1984 and 1991, Plummer et al., described the discovery^46^ and purification^47^ of a novel enzyme PNGase F, an amidase (amidohydrolase) that hydrolyzes the asparagine side chain amide bond of a wide variety of glycoprotein/glycopeptide substrates, generating an aspartic acid residue on a protein backbone and liberating the 1-amino oligosaccharide (glycosylamine). The latter slowly hydrolyzes nonenzymatically to ammonia and an oligosaccharide with a di-*N*-acetyl-chitobiose unit at the reducing end. The ability to cleave the major *N*-oligosaccharide classes of human glycoproteins, without the use of any other enzyme and under mild reaction conditions, makes the PNGase F a perfect candidate for obtaining released *N*-glycans in HT analysis.^48^

In the late 1980s, when Karas et al., were developing MALDI-MS, they already recognized its relevance for the MS-based analysis of saccharides. In the publication of 1987, which introduces MALDI for the analysis of nonvolatile compounds, it was demonstrated that ultraviolet laser desorption (UVLD)-MS analysis of stachyose (Gal(α1→6)Gal(α1→6)Glc(α1↔2β)Fru, where Gal, galactose; Glc, glucose; Fru, fructose) benefitted from the use of an ultraviolet (UV)-absorbing chemical matrix, nicotinic acid (NicAc), or tryptophan (Trp) to produce quasimolecular ions ([M + Na]^+^) of stachyose. The matrix-assisted analysis showed superior sensitivity compared to the UVLD-based analysis.^49^ A few years after, in 1993, Harvey showed the quantitative capabilities of MALDI-MS in oligosaccharide measurements.^50^ He confirmed a linear relation between oligosaccharide concentration and measured MS signal intensity over several orders of magnitude in analyte concentration. Additionally, it was shown that MALDI-MS is able to detect low femtomol amounts of various oligosaccharides using 2,5-dihydroxybenzoic acid (DHB) as a matrix, highlighting the usability of MALDI-MS as a comprehensive readout for complex oligosaccharide mixtures with large variations in dynamic range.

In 1997, Rudd et al., described the release of *N*-glycans directly from a band on a sodium dodecyl sulfate-polyacrylamide gel electrophoresis (SDS-PAGE) gel using PNGase F.^51^ The reducing termini of the *N*-glycans were labeled with 2-AB to allow direct quantitation from the HPLC profiles. An amide-silica HPLC column enabled high-resolution separations of both charged and neutral *N*-glycans. The equivalent approach combined with exoglycosidase digestions was used by Küster et al. in 1997 to profile *N*-glycans by MALDI-MS and HPLC.^52^ In 2008, Royle et al. presented a robust, fully automatable technology platform, including software for the detailed analysis of *N*-linked glycans released from glycoproteins, which included sample immobilization in 96-well plates, glycan release with PNGase F, and fluorescent labeling.^53^ Relative quantitative HPLC analysis included the monosaccharide sequence, linkage, and arm-specific information for charged and neutral *N*-glycans, as well as an automatic structural assignment of peaks from HPLC profiles via web-based software that accessed the GlycoBase database of more than 350 *N*-glycan structures, including 117 present in human serum glycome. Another software (autoGU) was used to stepwise analyze data from exoglycosidase digests to generate a refined list of final structures. *N*-Glycans from a 96-sample plate could be released and purified in two or three days and profiled in two days. Although the SDS-PAGE and in-gel deglycosylation of IgG are unfavorable for HT analysis, these methods represented a milestone for the further development of HT approaches for released *N*-glycan analysis, especially the latter method in 96-well plate format. The first application of this method on a large cohort revealed variability, heritability, and key environmental factors that influence plasma *N*-glycome composition.^27^

The development of MALDI-MS instrumentation in the years following its introduction resulted in the recognition that the analytical tool is perfectly suited for HT analysis. Initially recognized by Hsieh et al. in the context of pharmaceutical compound screening, MALDI-MS was praised for its relative ease of the sample preparation and minute amounts of sample required, its high salt tolerance compared to electrospray ionization (ESI)-based platforms, the large mass range, and its measurement sensitivity.^54^ In the presented context and in the early days of HT MALDI-MS platforms, its use was able to increase throughput to approximately 10 s per sample. Hsieh et al. concluded the work with the mention that upon the introduction of MALDI-MS as a HT readout platform, the main throughput-limiting factor in the wet lab would become the sample preparation.^54^ However, the ease and repetitive nature of the MALDI-specific sample preparation steps and the similarities of the MALDI target plate to a well-based sample plate make the complete MALDI-MS workflow extremely compatible with automation strategies. As mentioned above, the conversion of sample preparation to the 96-well format was a major breakthrough for HT glycomics. One of the more common strategies for the MALDI-MS-based analysis of released *N*-glycans from complex protein mixtures, or purified proteins is through the capturing of proteins on a polyvinylidene fluoride (PVDF) membrane and subsequent enzymatic removal of the glycans from their carrier proteins. The use of PVDF membranes for protein sequencing^55^ and *N*-glycan analysis^56,57^ originates from the late 1980s to mid 1990s of the previous century. Papac et al. were the first to implement this workflow in a 96-well format for the effective deglycosylation of 50 μg of the glycoprotein recombinant tissue-type plasminogen activator (rt-PA).^58^ The basic workflow consisted of capturing the glycoproteins on the PVDF membrane, a reduction and alkylation of disulfide bonds disrupting the tertiary and quaternary of proteins as well as protein complexes, and blocking of all open PVDF protein binding sites using a polyvinylpyrrolidone polymer (PVP360). Prior to MALDI-MS analysis, a 3 h enzymatic deglycosylation step using PNGase F was performed and released *N*-glycans were desalted using cation exchange. This initial report showed the possibility of deglycosylating and analyzing 60 samples in 8 h, effectively being 8 min per glycoprotein sample.^58^

In parallel with developments of HT approaches for HPLC *N*-glycan analysis, innovative work by Callewaert et al. in the 2000s has set grounds for *N*-glycan analysis by CGE technology using widely available DNA sequencers.^59−61^ The workflow was equivalent to the one developed for HPLC *N*-glycan profiling consisting of enzymatic glycoprotein digestion by PNGaseF, desalting, fluorescent labeling with APTS, and cleanup procedure performed in 96-well plates. In 2004, Guttman et al. presented an automated 96-capillary array that allows the analysis and characterization of mono- and oligosaccharides.^62,63^ However, as this workflow did not involve the enzymatic release of *N*-glycans, an improved sample preparation method was introduced that could be applied to study the *N*-glycome of glycoproteins^64^ and to study for IgG specific glycosylation.^65^

Another breakthrough in HT *N*-glycan analysis was the deployment of the so-called in-solution *N*-glycan release and labeling instead of deglycosylation of immobilized proteins in SDS-PAGE gels as reported by Royle et al. in 2008.^53^ Not only was the in-gel method demanding in terms of invested labor, it also showed lower efficiency of recovering *N*-glycans from the gel, which affected the overall reproducibility of the method. In 2008, Ruhaak et al. published a protocol where plasma proteins were first denatured with SDS, followed by overnight incubation at 37 °C for the *N*-glycan release using PNGase F, all in the same 96-well plate.^66^ The samples were further processed in the same plate, released *N*-glycans were labeled with a mixture of a fluorescent dye 2-aminobenzoic acid (2-AA) and a reducing agent NaBH_3_CN in dimethyl sulfoxide (DMSO)/glacial acetic acid (10/3; *v*/*v*), a method developed a couple of years earlier by Bigge et al. in 1995.^67^ Labeled *N*-glycans were then purified using a HILIC-based solid-phase extraction (SPE) method with microcrystalline cellulose as the stationary phase. Eventually, the purified 2-AA labeled *N*-glycans were separated using normal phase HILIC-HPLC. This workflow was further optimized in 2010 to enable the separation and detection of released total plasma *N*-glycans by CGE-LIF.^68^*N*-Glycans were released enzymatically from denatured plasma glycoproteins in the same manner already described in 2008.^66^ However, labeling was performed by using a fluorescent dye 9-aminopyrene-1,4,6-trisulfonic acid in citric acid and 2-picoline borane (2-methylpyridine borane) complex as a nontoxic and efficient reducing agent in DMSO.

Large-scale studies on individual glycoprotein glycomes were partially hindered by the lack of methodologies that could enable fast and robust purification of a glycoprotein of interest from a large number of samples at an affordable price. Protein purification was therefore one of the major bottlenecks in large-scale proteomics and glycomics studies. IgG *N*-glycome analysis has largely been facilitated by the development of a Protein G monolithic 96-well plate. In 2011, Pučić et al. published the first large-scale population study of the IgG glycome applying a novel HT method for isolation of IgG from 2298 plasma samples and IgG *N*-glycan analysis using HILIC-UHPLC-FLD.^69^ The entire procedure for 96 samples, including the binding of plasma samples, washing, and elution of isolated IgG, was performed in less than 1.5 h and provided a significant milestone in HT analysis of IgG *N*-glycome, enabling subsequent large-scale population studies and genome-wide association studies (GWAS).^70,71^

In 2013, Burnina et al., developed a practical procedure to prepare fluorescently labeled *N*-glycans for both qualitative and quantitative analysis by MS and UHPLC.^72^ In a single 96-well filter plate with a hydrophobic membrane, antibodies (trastuzumab) samples were denatured, reduced, and deglycosylated by PNGase F digestion. The released *N*-glycans were then fluorescently labeled (InstantAB label for a rapid labeling procedure; 5 min at 37 °C) in a collection plate before being filtered using a hydrophilic membrane filter plate. The complete sample preparation method took less than 90 min and was done entirely in ready-to-use 96-well plates with simple buffer systems. These novel rapid labeling chemistries relied on the presence of glycosylamines, and therefore a fast deglycosylation step is required. On the basis of the research from Ruhaak et al.^66^ and Burnina et al. in 2015. Trbojević-Akmačić et al. further optimized a workflow for HT analysis of released *N*-glycans from plasma by HILIC-UHPLC-FLD^73^ by combining in solution deglycosylation, 2-AB labeling, and cleanup using only a hydrophilic membrane filter plate, which was the basis for some of the largest HT glycomic studies on total plasma/serum *N*-glycome and IgG *N*-glycome by the same technology.^74−78^

With the development of faster sample preparation protocols and analysis of large sample numbers, new challenges emerge regarding data processing and analysis. For separation-based techniques (e.g., UHPLC), manual peak integration is one of the most time-consuming tasks. Although (semi)automatic integration methods do exist in the proprietary software tools used for data acquisition, these are often not adapted for large cohorts due to small shifts in peak retention times that happen with the time of analysis. A significant shift in decreasing processing time occurred with the development of an automatic semisupervised method for peak integration.^79^

Although the detection and identification of *N*-glycans in HT glycomics, in general, predominantly relies on UHPLC, CGE, and MS methods,^80^ the drawbacks of these analytical platforms are the need for expensive equipment and complicated sample preparation steps, making lectin-based methods attractive for glycosylation research.^81^ Because of their high specificity and affinity toward glycans, lectins are another promising tool for glycan detection and the study of cells’ glycosylation. Using carbohydrate–lectin interactions, problems like separation and purifying carbohydrate-containing biomolecules are solved.

Lectins are a group of proteins that contain at least one noncatalytic site for specific and reversible binding to carbohydrates and the carbohydrate determinants of biopolymers without changing their structures.^82,83^ They are widely distributed in nature and have been isolated from viruses, fungi, bacteria, invertebrates, unicellular organisms, animals, and plants.^84,85^ Lectins play an important role in the immunological defense against pathogens,^86^ blocking of viral infections,^87^ regulation of microbial cell adhesion and migration,^88^ and control of protein levels in the blood.^89^

The carbohydrate-binding properties of lectins have historically been used for the separation and purification of glycoproteins, glycopeptides, and oligosaccharides by affinity chromatography (covalently bound to agarose, silica, or other polymer stationary phases),^90−93^ as histochemical and cytochemical reagents for detection of glycoconjugates in tissue sections, on cells and subcellular organelles,^94,95^ and in investigations of intracellular pathways of protein glycosylation and study of membrane transport,^96^ as well as for sorting cells using flow cytometry.^97^ Nowadays lectin microarrays are widely used as an analytical tool in various applications, e.g., to investigate the glycosylation in diverse cells during cell development and differentiation for analyzing cell processes, including cell differentiation and development, cell–cell communication, cell surface biomarker identification, and pathogen–host recognition.^98^

The first reports on lectin microarrays were published in 2005,^99−102^ and already in 2007 Rosenfeld et al. developed GlycoArray kits that enabled glycosylation analysis of intact glycoproteins within 4–6 h.^103^ This lectin microarray technology, utilizing nitrocellulose membrane-coated glass slides and labeled secondary antibodies with automated data analysis, showed high potential as a first-line tool for HT analysis directly from complex biological samples.

Development of sample preparation approaches for lectin microarray analysis allowed glycan profiling at the subnanogram level.^104^ However, lectin microarray application in HT glycomics has been lagging behind other, above-mentioned, techniques. Major reasons for that have been the limited repertoire of naturally occurring lectins (mostly from plants) that cannot detect all human glycosyl epitopes as well as insufficient sensitivity for some clinical samples and therefore rely on other techniques for structural elucidation.^105^ Even though they are currently not used in a HT glycomic analysis of hundreds to thousands of samples per cohort, lectin microarrays have recently been used for the analysis on the level of 50 to hundreds of samples. For example, this technology has been used in different applications from salivary glycoprotein analysis in type 2 diabetes mellitus,^106^ glycan analysis in hepatocellular carcinoma specimens^107^ and serum of prostate cancer (PCa) patients,^108^ IgG glycan analysis in primary biliary cholangitis,^109^ to seminal plasma glycome analysis in fertile and infertile subjects,^110^ demonstrating a perspective for future use on a larger scale. Lectins have also recently been employed in lectin-based biosensors.^111,112^

## 5 Technologies

**Figure 5:**
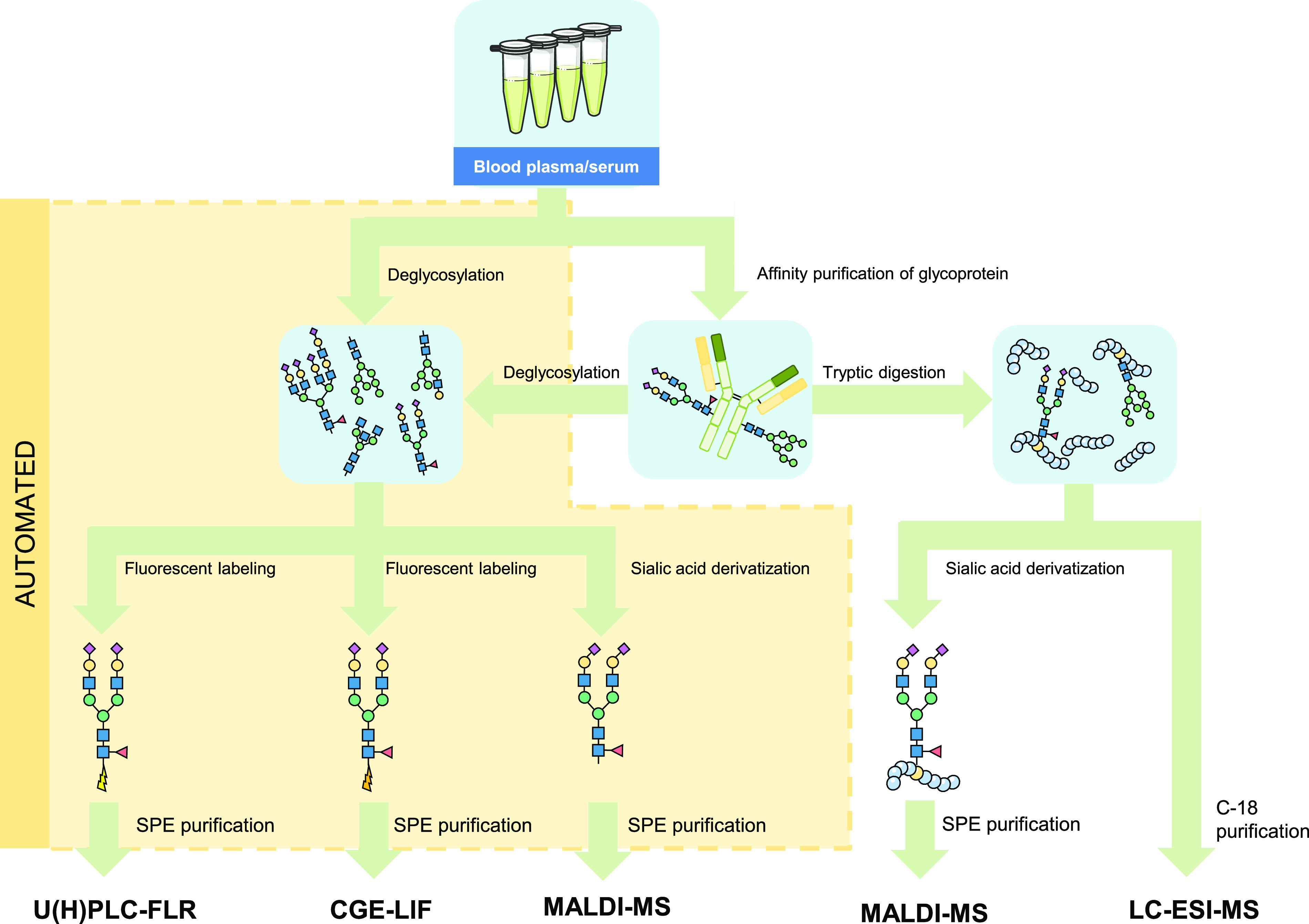
A diversity of workflows are available for HT glycomic analysis. For the different derivatization and labeling procedures as well as specific labels, see Figures 3 and 7, respectively. Please note that the sialic derivatization step of the glycopeptides will also modify the peptide backbone.

**Figure 6:**
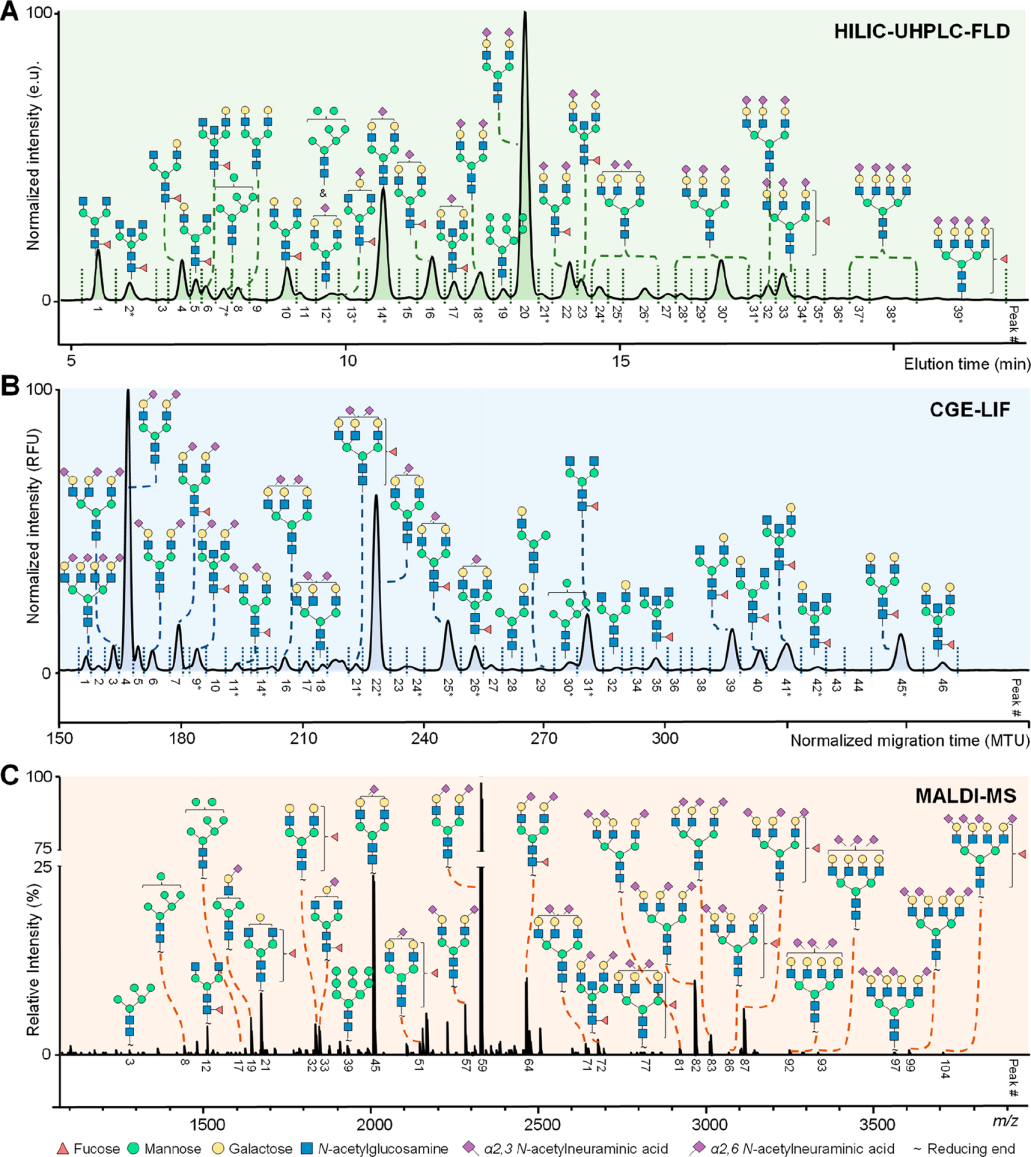
Exemplary profiles of the total serum *N*-glycome using (A) HILIC-UHPLC-FLD, (B) CGE-LIF, and (C) MALDI-MS. (A) Chromatogram after 2-AB labeling by HILIC-UHPLC-FLD. (B) Electropherogram after APTS labeling by CGE-LIF. (C) Mass spectrum after differential sialic acid esterification by MALDI-FT-ICR-MS. The assigned signals in the MS spectra correspond to [M + Na]^+^. Please note that HILIC-UHPLC-FLD and CGE-LIF provide (in some cases) isomer separation in regard to branching (galactose arm, bisection, and fucose position). Structures are assigned based on exoglycosidase treatment and/or tandem MS data as well as literature knowledge on biosynthetic pathway of *N*-glycans. *Some signals for the HILIC-UHPLC-FLD and CGE-LIF correspond to multiple *N*-glycan compositions for which the most abundant one is assigned in the figure.

HT glycomics relies on several technologies utilized for glycan profiling, mostly on the level of released glycans or glycopeptides. These technologies are often used in parallel because they offer different advantages and provide complementary information, e.g., UHPLC-FLD and LC-MS. Although general approaches in the sample preparation workflows are similar (Figure 5), each of these technologies also require some specific analytical conditions and data processing steps to obtain reliable and robust data (Figure 6).

### 5.1 Liquid Chromatography

Currently, several HT approaches for *N*-glycan analysis are in use, with HILIC-UHPLC-FLD being the most prevalent.^113^ While in the 1970s and 1980s glycan preparation workflows generally required sizable quantities of starting material, long hands-on time for sample preparation, and long analysis runs, the advancement of applicable chemistries, miniaturization of reaction volumes, and column particle technologies enabled UHPLC to become one of the most robust HT technologies used for glycan analysis. The UHPLC in combination with FLD allows complete characterization of complex glycan mixtures in a relatively short time and, although it requires high-end instrumentation, is much less expensive than MS. Thus, UHPLC is the method of choice for routine analysis of protein glycosylation with previously characterized glycan structures. For characterization of a novel glycan structure, UHPLC may be coupled with other methods that can provide further structural information, especially MS with LC-MS techniques.

#### 5.1.1 Sample Preparation

**Figure 7:**
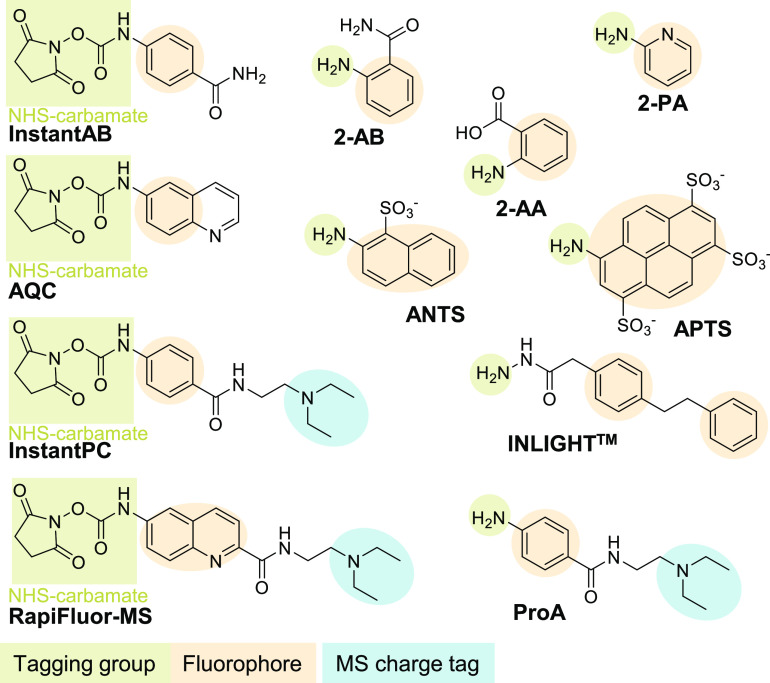
A diversity of commonly used labels that can be attached to the reducing end of the glycan. Labeling is performed to enable fluorescence detection (all labels), to improve retention on RP-LC-FLD (2-AA, 2-AB, and 2-PA), to enable separation by introducing a negative charge for CGE-LIF (APTS, ANTS, 2-AA, or 2-PA), or to enhance MS ionization by introducing a tertiary amine through carbamate chemistry (InstantPC, RapiFluor-MS, ProA). 2-AB, 2-AA, and 2-PA can also be used as reactive MALDI matrices as these labels absorb UV light that is in the wavelength range of most common MALDI lasers (330–360 nm).

UHPLC is widely used for the analysis of released glycans, which means that prior to their separation and detection, glycans must be cleaved of their protein carrier. Samples are prepared usually by employing enzymatic deglycosylation by PNGase F, the most widely used enzyme in HT studies, fluorescent labeling of released glycans, cleanup procedure, and chromatographic analysis. Of note, while PNGase F is widely used in released *N*-glycan analysis, it appears to have poor efficiency for releasing highly truncated *N*-glycans with, e.g., only chitobiose or a single *N*-acetylglucosamine that appear to be rather common.^114,115^ Sometimes, an additional step of a cleanup procedure is being used after deglycosylation, facilitating the fluorescent labeling reaction efficiency.^116,117^ Historically, 2-AB has been the most widely used fluorescent label, starting from the first HT studies performed on the level of thousand samples.^27,69,118,119^ Although it results in high fluorescent signals, it is not easily ionizable, hindering MS characterization of labeled *N*-glycan species. Several other labels have recently been more and more applied, e.g., procainamide (ProA),^120^ 6-aminoquinolyl-*N*-hydroxysuccinimidyl carbamate (AQC),^121^ and RapiFluor-MS^122^ (Figure 7), showing equivalent or enhanced fluorescence compared to 2-AB, as well as ionization in the cases of ProA and RapiFluor-MS due to the introduction of a charged tertiary amine tag.^117,120,123,124^ Moreover, INLIGHT labeling strategy has been used to increase hydrophobicity and ionization of *N*-glycans for more efficient RP-LC-MS analysis.^125,126^ A typical fluorescent labeling reaction with 2-AB or ProA is performed at 65 °C for 2–3 h, creating a balance between labeling efficiency and the loss of sialic acids. With the development of rapid labeling chemistries (Figure 7), e.g., AQC,^127^ InstantAB,^128^ InstantPC,^129^ and RapiFluor-MS,^130^ the reaction time is significantly decreased to only 5 min, making this approach even more HT. Although rapid labeling chemistries significantly decrease the hands-on time, they suffer from a narrow range of starting sample quantity because all amine groups from the sample (*N*-glycosylamines, proteinaceous amines, other free amines) are being labeled, requiring a large excess of the labeling reagent. Therefore, rapid labels are currently more applicable to isolated glycoprotein *N*-glycan analysis (e.g., IgG) than for more complex samples like plasma/serum, where the level of amines can vary significantly between individual samples and might be more difficult to optimize before analysis. As the newly introduced labels also result in differential elution order of *N*-glycans, when it is FLD-based, it becomes complicated to compare the obtained results with previous studies as well as for data integration. Therefore, 2-AB is still widely used even though other labels have shown their advantages.

The cleanup procedure is generally performed by HILIC-SPE using different hydrophilic stationary phases, e.g., hydrophilic filters (in the form of 96-well plates)^72,73^ and hydrophilic bead-based cartridges or plates^73,117^ because these have proven to be effective in the removal of excess reagents and proteins from the previous sample preparation steps. Additionally, magnetic nanoparticles can also be used as a cleanup approach.^120,131^

Different variations of the standard glycan preparation workflows have recently become available in a kit format, e.g., GlycoWorks RapiFluor-MS *N*-Glycan Kit (Waters), AdvanceBio Gly-X *N*-Glycan Prep Kits (Agilent), and Glycoprofile Labeling Kits (Merck), expediting their application in basic HT glycomic research and biomarker discovery as well as automation.

#### 5.1.2 Measurement and Data Processing

Fluorescently labeled glycans are generally analyzed in HT mode by HILIC-based LC due to its remarkable capabilities to separate polar and hydrophilic analytes in an aqueous–organic mobile phase. A linear gradient with an increasing percentage of 50 or 100 mM ammonium formate in acetonitrile enables efficient separation of *N*-glycan species depending on their charge, size, and linkages. Column chemistries are based on modified amide-based residues with 1.7–1.8 μm particles. Recent advancements in the column hardware surface preparation reduce the interactions between glycans and the metal surface of the column, resulting in improved peak recovery, shape, and reproducibility, which is especially important for sialylated species.^79^ After manual or automatic^79^ integration, total area normalization is usually used to extract glycan amounts as relative percentage areas (%area) used for further analysis, followed by batch correction and statistical analysis. Only recently, a systematic evaluations of other normalization methods for glycomics data, in addition to total area normalization, have been reported.^132,133^

#### 5.1.3 Peak Assignment/Glycan Identification

GU values based on fluorescently labeled dextran ladder as an external standard are appointed to individual glycan structures separated by HILIC-UHPLC-FLD and used for the peak assignment. GU values are more stable than retention times of individual glycans, which can vary depending on the (U)HPLC system and chromatographic conditions used for the analysis and are usually used as reference values in the databases. Nowadays, extensive HPLC/UHPLC glycan databases containing GU values exist, although these are predominantly populated with 2-AB labeled glycans data, while migration information for alternatively used fluorescent labels is still largely missing and is one of the goals for future advancement of HT glycomic applications.

**Figure 8:**
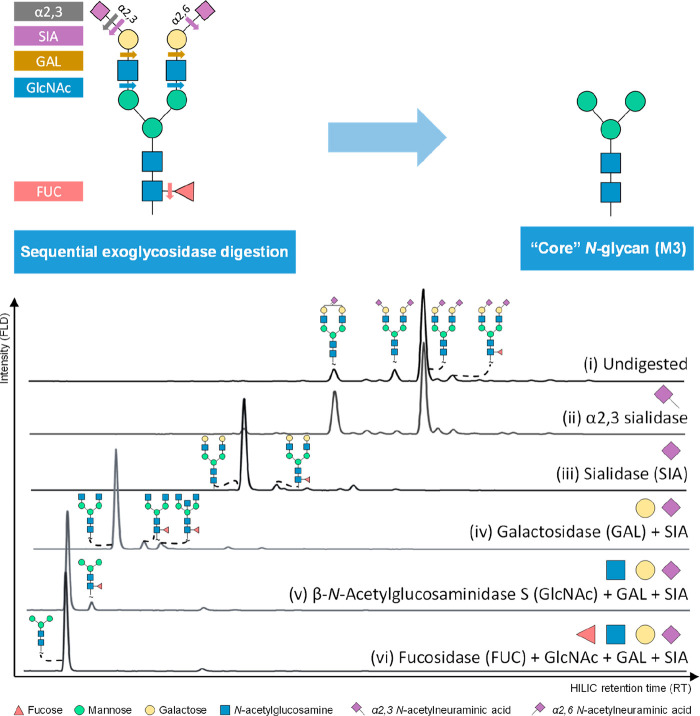
Exoglycosidase digestions of 2-AB labeled human plasma transferrin (Tf) *N*-glycans. (i) Undigested samples. (ii) *Streptococcus pneumoniae* α2–3 neuraminidase S. (iii) *Arthrobacter ureafaciens* α2-3,6,8,9 neuraminidase A (SIA). (iv) *Streptococcus pneumoniae* β1–4 galactosidase S (GAL) + SIA. (v) *Streptococcus pneumoniae* β-*N*-acetylglucosaminidase S (GlcNAc) + GAL + SIA. (vi) *Omnitrophica bacterium* α1-2,4,6 fucosidase O (FUC) + GlcNAc + Gal + SIA.

Exoglycosidase sequencing (glycan trimming with enzymes specific for different types of terminal sugar monomers) has been historically used as a complementary approach to indicate specific structural features found on glycans eluting in individual chromatographic peaks (Figure 8).^51,134,135^ Although it is worth noting that exoglycosidase sequencing has to be accompanied with MS characterization,^69^ either off-line after chromatographic peak collection or online by connecting UHPLC system to MS detector. Of note that, depending on the fluorescent label used for released glycan derivatization, exoglycosidase sequencing conditions might have to be optimized to achieve reliable results.^136^ In contrast to coupling CE to MS, the buffers used for HILIC separation are compatible with MS ionization techniques and recently used fluorescent labels have functional groups designed to facilitate ionization and UHPLC-MS analysis, making this approach highly desirable for structural characterization of glycans.

### 5.2 Capillary (Gel) Electrophoresis

Historically, CGE-LIF has been mostly employed for DNA sequencing.^137^ However, because it has been used for fetuin *N*-glycan analysis in 1996 by Guttman et al.^138^ and AGP *N*-glycan analysis in 2001 by Callewaert et al.,^59^ it has shown great potential for sensitive *N*-glycan analysis, and as 96 capillaries can be used in parallel, it theoretically allows for very HT. Practically, it has been mostly used in 4-, 8-, or 16-capillary setup due to capillary-to-capillary variations as well as the cost-effectiveness of analyzing smaller cohorts. Compared to UHPLC and MS, it offers advantages such as higher sensitivity and the separation of isobaric glycan structures, respectively, without sacrificing robustness and cost of analysis.^61^ However, its large-scale application in HT glycomics has been hindered by underdeveloped databases for glycan peak annotation and the lack of glycan standards. Moreover, coupling of CGE to MS is still challenging, making structural annotation of glycan peaks rather complex. On the other hand, capillary electrophoresis (CE) in combination with positive ion mode ESI-MS via a sheathless interface allows efficient droplet desolvation and analyte ionization. Although, because of longer analysis time and lower robustness, it has been mostly used for in-depth profiling and not in HT glycomics.^139^ Nevertheless, CGE-LIF has been routinely used for IgG *N*-glycan profiling both in biopharma studies^140^ and HT population and clinical studies^141^ and less routinely for other isolated glycoproteins and total plasma/serum protein *N*-glycome analysis.^141,142^

#### 5.2.1 Sample Preparation

The sample preparation approach for CGE-LIF analysis is analogous to the approach used for UHPLC analysis of free *N*-glycans, and it consists of (usually enzymatic) deglycosylation, fluorescent labeling of free *N*-glycans, and cleanup procedure followed by CGE separation with LIF detection. In contrary to UHPLC fluorescent labels, the ones used for subsequent CGE-LIF analysis are charged, next to having a fluorophore, to allow electrophoretic separation of all glycans (not exclusively sialylated species).

The most commonly used dye is a triply negatively charged label, APTS, which is coupled to the *N*-glycans by reductive amination using NaBH_3_CN or nontoxic 2-picoline borane as a reducing reagent and acetic or citric acid. Acetic acid has been frequently used in the first studies and required a significant excess of APTS (more than 100-fold) to achieve a 95% glycan labeling efficiency.^143^ In addition to the high cost of analysis, excessive reagent amounts require extremely efficient cleanup procedures to obtain clean fluorescently labeled *N*-glycans and ensure reproducibility of analysis. Because temperature and duration of the derivatization procedure are a tradeoff between fast and efficient labeling on one hand and minimal desialylation (potentially skewing the results), on the other hand, this step requires optimization and fine-tuning. Váradi et al., have demonstrated that APTS glycan labeling in the presence of acetic acid has approximately 50% lower efficiency in a 2 h reaction compared to overnight labeling but decreases loss of sialic acids.^144^ Citric acid, which is stronger than acetic acid, enables a faster labeling reaction (50 min at 50 °C in contrast to an overnight incubation at 37 °C) with 10 times lower use of APTS and little or no loss of sialic acids.^64^ Therefore, labeling reactions in HT studies are performed in solution using citric acid. An alternative fluorescent label has been used for IgG *N*-glycan labeling and subsequent CGE-LIF analysis, e.g., singly negatively charged 2-amino-1-naphthalenesulfonic acid (2-ANTS), which resulted in drastically reduced glycan separation and more efficient ionization in CE-MS analysis compared to APTS-labeled IgG *N*-glycans.^145^

The introduction of a cleanup procedure using Sephadex G10 packed 96-well filter plates enabled *N*-glycan profiling at low picomolar amounts of the glycoprotein.^59^ Previously, *N*-glycans had been analyzed immediately after labeling and sensitivity of detection had been achieved by diluting the reaction mixture prior to analysis in order to lower the concentration of contaminants.^138^ The cleanup procedure is nowadays mostly done via HILIC-SPE^68^ as in the UHPLC-FLD *N*-glycan analysis. Alternative approaches like magnetic bead-based sample preparation have also been used.^144^ These are based on magnetic microparticles coated with carboxyl groups that reversibly bind to APTS labeled *N*-glycans with hydrophilic interactions in >80% acetonitrile environment (which acts as a crowding reagent), while deglycosylated proteins and excess reagents are washed away. Another mechanism of purification using carboxyl-coated magnetic beads that can readily be used is binding of released *N*-glycans (in positively charged glycosylamine form) by ionic interactions immediately after PNGase F release. In this later case, released *N*-glycans are eluted from the beads with aqueous APTS solution followed by the addition of the reducing agent to immediately initiate the labeling reaction without any interim concentration steps.^144^ This protocol enables faster sample preparation with the possibility for automation,^146^ although it has been mostly used on a lower scale for therapeutic antibodies *N*-glycan analysis due to higher cost per sample.

In recent years, HT application of CGE-LIF technology has been facilitated by the development of *N*-glycan preparation kits, e.g., Glycan Assure APTS Kit (Thermo Fisher), Fast Glycan Labeling and Analysis Kit (SCIEX), AdvanceBio Gly-X *N*-Glycan Prep Kit (Agilent), and glyXprep Sample Preparation Kit (glyXera), containing all reagents needed for a typical CGE-LIF workflow–glycoprotein deglycosylation, released *N*-glycan purification, APTS labeling, and removal of excess reagents. Although less cost-effective for large cohort analysis, sample preparation kits enable a more streamlined automatable solution in the case of *N*-glycan analysis in the standard types of samples, e.g., IgG *N*-glycans. This approach has been recently used for glycan analysis on IgG Fc fragment by CGE-LIF after IgG isolation and on-beads IgG digestion with IdeS to analyze IgG-Fc *N*-glycans in latent, active, and treated tuberculosis patients and healthy controls (*n* = 83).^147^ The same analytical approach has been used both for IgG *N*-glycan analysis and total plasma protein *N*-glycan analysis by CGE-LIF in a cohort of post-treatment controllers and post-treatment noncontrollers of human immunodeficiency virus (HIV) after antiretroviral therapy (ART) termination (*n* = 98),^148^ demonstrating a potential application of used technology for robust quantification of glycans as noninvasive plasma biomarkers.

#### 5.2.2 Measurement and Data Processing

Fluorescently labeled negatively charged glycans are electrokinetically injected into capillaries by applying a low voltage for a short period of time. Injected glycans migrate in the applied electric field through capillaries and are being separated based on their hydrodynamic volumes and their mass-to-charge ratios or, as recently demonstrated for HMOs, based on the secondary equilibrium of the borate–vicinal diol complexation.^149^ Migration time alignment standards (coinjected bracketing standards) are used to minimize migration time shifts between samples and facilitate glycan identification and quantification, by enabling electropherogram alignment and GU unit assignation. After manual or automated peak integration, total area normalization is usually used to extract glycan amounts as relative %area used for further analysis, again followed by batch correction and statistical analysis. Alternatively, total height normalization can also be used to obtain relative peak height proportions (%rPHP).

#### 5.2.3 Glycan Structure and Characterization

Analogous to UHPLC, structures of glycans separated by CGE-LIF are also elucidated by comparison of individual glycan peak glucose unit (GU_CGE_) with the GU_CGE_ values of specific glycan structures in available databases and utilization of exoglycosidases sequencing.^142,150^

GU_CGE_ values are assigned based on fluorescently (e.g., APTS) labeled standard oligosaccharide ladder, usually maltodextrin (homopolymer of α1,4-linked glucose), although dextran has also been used. The retention time of each unknown oligosaccharide correlates with the length of the sugar oligomer and is converted to a GU_CGE_ scale used for a database search. It is of utmost importance that the same standard oligosaccharide ladder is used for analysis and the database buildup because CGE migration depends on hydrodynamic volumes affected by the molecular configuration and conformation.^151^ The development of databases containing CGE-LIF separated glycans has been lagging behind HPLC/UHPLC glycan databases due to more complex structural confirmation of individual glycans caused by difficulties of CGE coupling to MS. However, this is slowly changing, and nowadays several expanding databases, e.g., GUcal^152^ (recently broadened with the GlycoStore data)^152,153^ and glyXbase,^154^ exist (Table 1). Populating these databases with glycans labeled with alternative fluorescent labels and originating from glycoproteins other than human IgG will facilitate the use of CGE-LIF technology for low- and HT glycomic studies.

Exoglycosidase sequencing has been used as a complementary approach to assist the glycan structure characterization both for *N*-glycans analyzed by CGE-LIF and UHPLC. Although labor-intensive and less straightforward for more complex samples (e.g., total plasma/serum protein glycan pool),^142^ this approach has been often used for elucidation and confirmation of glycans originating from isolated glycoproteins (e.g., IgG)^155^ during HT method establishment. Exoglycosidase sequencing is shown to be automatable by using a temperature-controlled sample storage compartment of a capillary electrophoresis (CE) machine for enzymatic reactions and the separation capillary for delivery of the exoglycosidase enzymes, speeding up the process.^155^

While a fluorescent label used for glycan derivatization should enable efficient glycan separation in CGE and ionization in MS, the coupling to MS has been challenging due to the incompatibility of gel and buffers used for CGE separation with MS analysis as well as maintaining the closed electrical circuit which is needed for the CE analysis. Therefore, to enable subsequent MS analysis the CGE separation needs to be sacrificed, which complicates the one-on-one comparison between CGE-LIF and CE-MS for glycan structure confirmation. Several attempts to connect capillary zone electrophoresis (CZE) to MS in the past have shown some promise.^156,157^ Recently, CZE combined with positive ion mode ESI-MS has been successfully employed for *N*-glycan profiling after linkage-specific derivatization of sialic acids and labeling the reducing end of all *N*-glycans with Girard’s reagent P.^139^ This approach was shown to be highly sensitive and applicable to free *N*-glycan analysis of a complex biological sample, total human plasma glycoproteins. Although it allows in-depth profiling of low abundant glycans, this approach is currently more suitable for low- to semi-HT glycan analysis due to the long separation times as well as capillary to capillary variation.

### 5.3 Mass Spectrometry of Glycans

**Figure 9:**
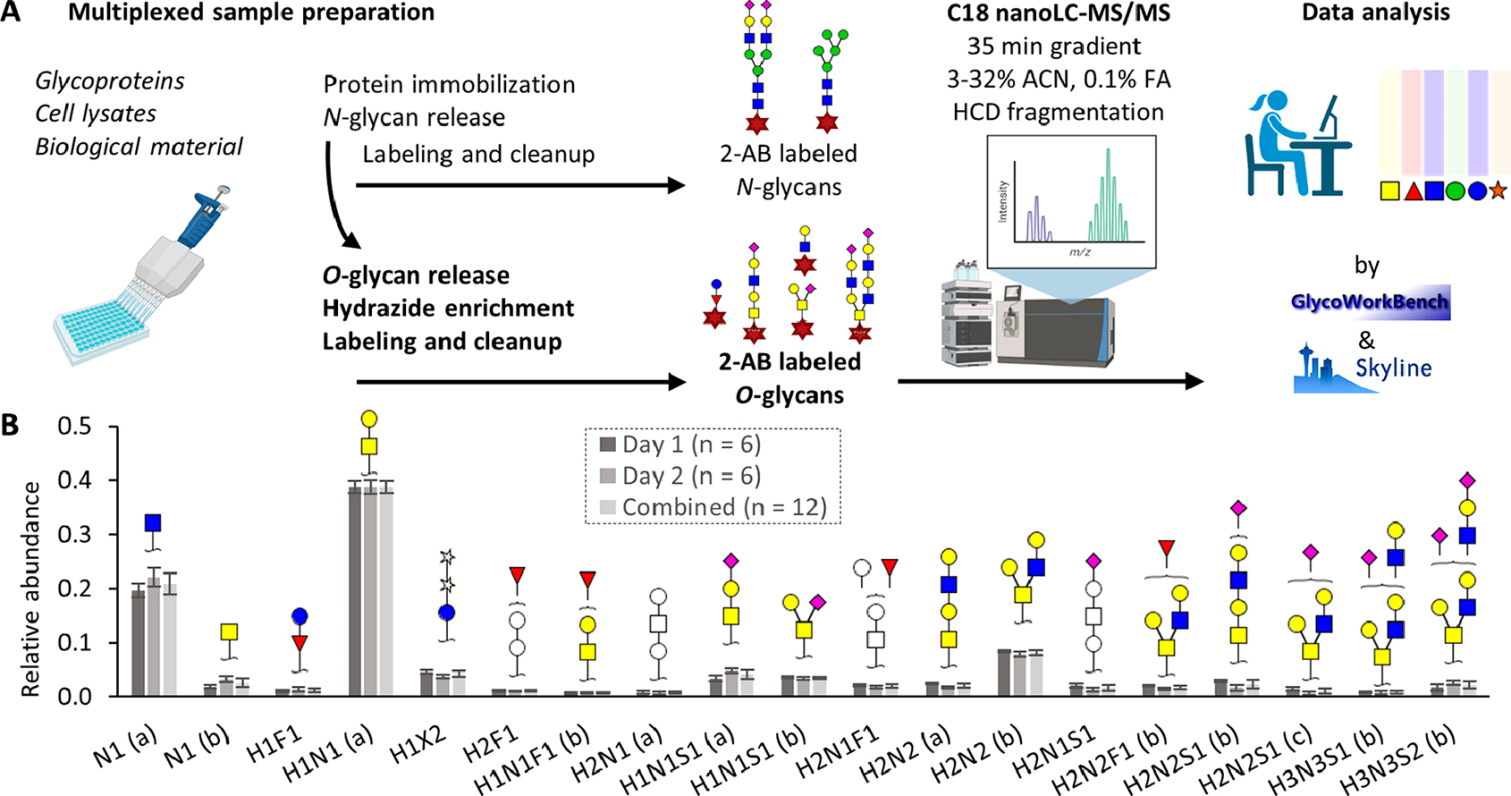
Multiplexed sample preparation workflow for *N*- and *O*-glycan profiling. Intra- and interday repeatability of the optimized method. (A) Proteins are immobilized on a polyvinylidene fluoride (PVDF) membrane by the addition of a (pure) glycoprotein, cell lysates, or derived from biological material (e.g., plasma). *N*-Glycans are released by the addition of PNGase F and eluted from the PVDF membrane. The *O*-glycans are released by adding a release agent and eluted from the PVDF membrane followed by a labeling procedure (2-AB). Eventually, the samples were analyzed using C18 nano-LC-MS/MS followed by data analysis. (B) Inter- and intraday repeatability of the *O*-glycan workflow. Average relative intensities for the *O*-glycans are displayed for those with a relative abundance above 1% per day. Error bars represent the standard deviations. Graphics in (A) were created using https://biorender.com/: H, hexose; N, *N*-acetylhexosamine; F, fucose; S, *N*-acetylneuraminic acid. Reproduced with permission from ref (24). Copyright 2022 de Haan et al.

The use of MS is common in glycomics research and extends to all oligosaccharide classes (*N*- and *O*-linked glycosylation; Figure 9,^24^ glycolipids,^158,159^ GAGs,^160,161^ and free saccharides^15^) and to all levels of glycosylation characterization.^162−166^ MALDI-MS is very powerful when it comes to rapidly generating molecular fingerprints of complex samples, which makes it the preferred analytical strategy for HT profiling studies of released *N*-glycans from either purified glycoproteins or complex sample matrices like liquid biopsies (i.e., full blood, plasma, serum). As such, MALDI-MS is most commonly used as a stand-alone platform. ESI-MS is commonly hyphenated to molecular separation methods (i.e., HPLC or CE as described above), which is a limiting factor when it comes to throughput. Hence, ESI-MS-based platforms are more commonly used for the analysis of more complex analytes in which MALDI-MS does not provide sufficient resolving power, dynamic range, or sensitivity, like for example glycopeptides.^167^

Different LC-MS and CE-MS approaches have been successfully employed for the analysis of complex sugar mixtures, e.g., HMOs, and recent developments in this area have been reviewed by Auer et al.^17^ In addition to LC-MS and CE-MS that enable limited glycan isomer separation, hyphenation of MS to ion mobility (based on a gas-phase separation) has emerged as another technique enabling analysis of glycan and glycopeptide isomers. Ion mobility MS does not rely on derivatization or enzymatic reactions for isomer identification. Instead, isoforms are identified based on collision cross-sections differences of fragment ions facilitating elucidation of a complex and diverse glycan repertoire, not applicable exclusively to *N*-glycans but also to other glycan classes, e.g., *O*-glycans, HMOs, GAGs, etc.^168,169^ This technique has been applied for glycan analysis in smaller sample sets^170−172^ and has the potential for the analysis of glycan isomers in larger cohorts.

#### 5.3.1 Sample Preparation

While the individual steps of the sample preparation workflow for hyphenated ESI-MS-platforms are largely defined by the molecular separation technology, the use of MALDI-MS brings some technology-specific aspects which include the spotting of the sample and MALDI matrix (or a mixture of both) on a target plate compatible with the MS platform.^173^ One additional step in the sample preparation that became very relevant upon the introduction of MALDI-MS analysis of released *N*-glycans, and the analysis of glycoconjugates by MS in general, was the stabilizing and linkage-specific derivatization of sialic acids through, for example, methyl^174^ or ethyl esterification^175^ and dimethylamidation.^176,177^ Beyond the scope of this review, a comprehensive review was published by De Haan et al., describing in great detail the different sialic acids, the different chemistries used for their derivatization, as well as their applications.^178^ However, the problems related to the MS-based analysis of sialylated *N*-glycans are multifaceted. First, *N*-glycans are most commonly analyzed as pseudomolecular cations, in the form of alkali-metal adducts. The negatively charged sialic acids have an adverse effect on the cation formation of sialylated species, which consequently results in quantitative biases of sialylated glycan species compared to neutral glycan species.^179,180^ Second, the sialylated glycan species are prone to forming a heterogeneous and unpredictable set of alkali-metal adducts. This results in a phenomenon called “peak splitting”, in which a single analyte population is represented by multiple ion species, different in their net mass through the incorporation of varying numbers or species of alkali-metal ions, and thus occupying different spaces in the *m*/*z* continuum. Typically, this results in ionization biases, a reduction of measurement sensitivity, and issues in peak annotation.^181^ Third, the sialic acid residue in sialylated glycan species is extremely fragile and prone to both in-source and post-source metastable fragmentation. Partial, as well as full loss of sialic acid residues, will result in loss of biologically relevant information and induce quantitative biases in complex glycan mixtures. Finally, sialylation introduces a large source of (biologically relevant) variation as the sialic acids can be bound to the rest of the glycan moiety through various linkages (i.e., α2,3, α2,6, α2,8, and α2,9). Sialylated glycans with multiple sialic acid residues often show linkage heterogeneity, resulting in a large number of potential isomeric glycan compositions. Without the exoglycosidase treatment (which cannot be considered HT), it is impossible to differentiate these in a typical MS1 analysis (which is common when using MALDI-MS), unless using chemical derivatization, which was shown to be feasible in HT fashion, for ethyl esterification by Reiding et al.^175^

To increase measurement sensitivity, substantial efforts were made to purify and concentrate glycans and/or glycopeptides prior to the spotting procedure. The most commonly used method in HT glycomics is the use of SPE applying the HILIC principle. The 96-well format adaptation of this method was pioneered by Selman et al.^182^ The presented method showed capable of analyzing 384 samples in less than 36 h using high-resolving power MALDI-Fourier transform ion cyclotron resonance (FTICR)-MS; less than 6 min per sample, of which approximately 18% (∼1 min) was consumed by the MALDI-FTICR-MS measurement.

Rather than using HILIC- or RP-SPE, glycoblotting has been proposed as an alternative for enrichment of released glycans or glycoconjugates from complex samples. Introduced by Nishimura et al. in 2005, the glycoblotting strategy is based on affinity purification of carbohydrates using beads coated with Fischer’s reagents.^183^ Following the mixing of the capture beads with a solubilized sample and the concomitant ligation of the carbohydrates to the Fischer’s polymers, the beads are separated from the liquid through spin filtration. Carbohydrates are then released from the beads and spotted with a MALDI matrix for MALDI-MS analysis. While the strategy has a high potential for HT application,^184,185^ it is limited by the high costs of the reagents.

An additional strategy to improve measurement sensitivity and decrease ionization bias, through reducing the metastable nature of the oligosaccharide ions, is the derivatization of the reducing end with a charge-containing or UV-reactive group. Mentioned previously as labels for fluorescence detection, 2-AA, 2-AB, and 2-PA absorb UV light in the wavelength range of the most common MALDI lasers (330–360 nm) and can thus be used as reactive MALDI matrices.^186−189^ Furthermore, reducing end derivatization using charge carriers Girard’s Reagent P and T have shown to have a beneficial effect on ionization in MALDI-MS applications.^139,190−192^

**Figure 10:**
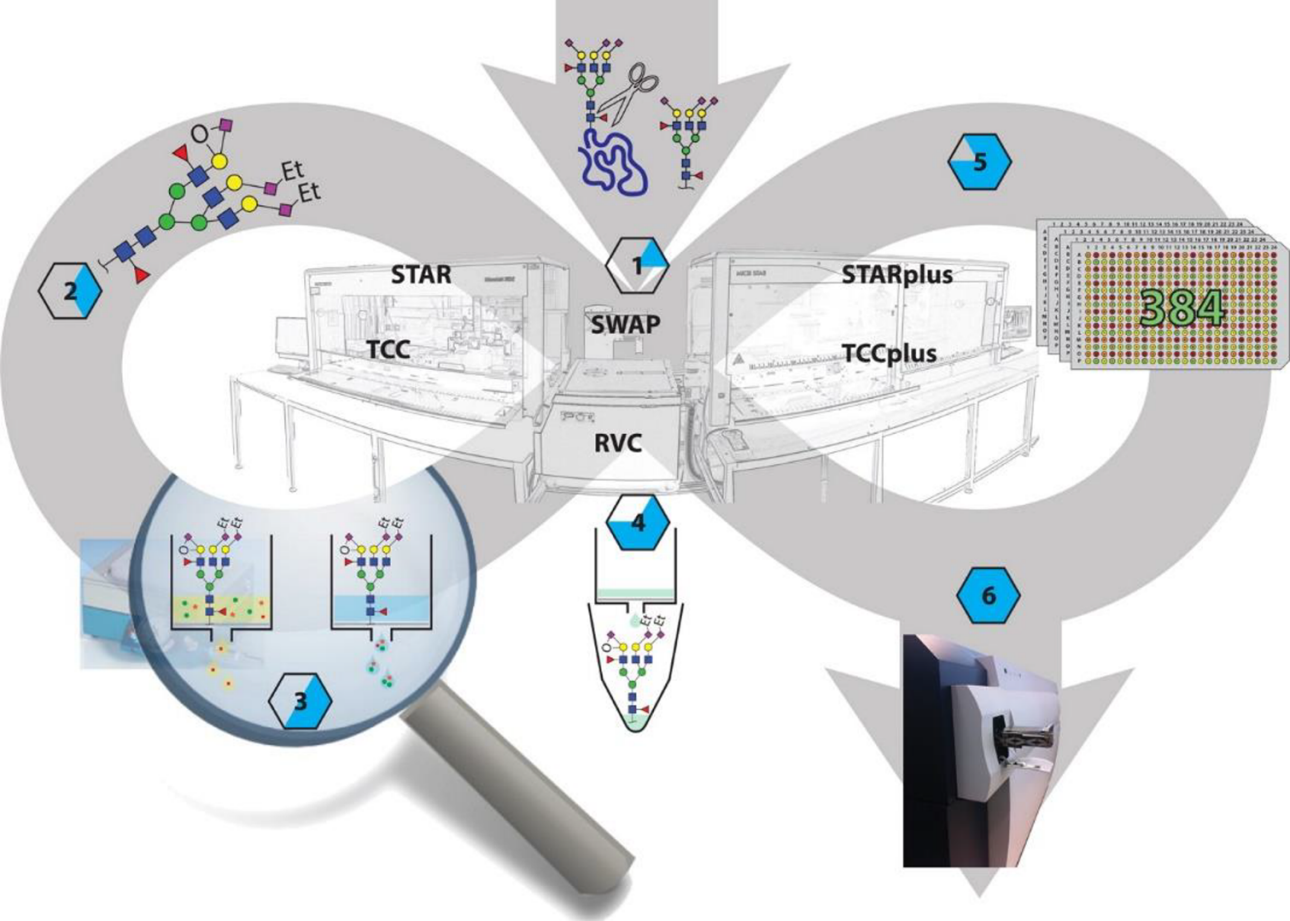
Automation of released *N*-glycan analysis by MALDI-TOF-MS. A graphical representation of the setup is provided and the consecutive processing steps are as follows: (1) PNGase F release, (2) ethyl esterification, (3) glycan enrichment by hydrophilic polypropylene (GH Polypro, GHP) HILIC–SPE, (4) glycans are eluted from the GHP membrane, and (5) MALDI target spotting followed by (6) MALDI-TOF–MS analysis. Reprinted with permission from ref (193). Copyright 2015 American Chemical Society.

Major strides forward in increasing throughput in MALDI-MS-based studies were achieved through the automation of the sample preparation by robotization.^193^ Doherty et al. explored this for the analysis of released IgG *N*-glycans using HILIC-UHPLC-FLD.^194^ Here, the most time-consuming steps of the preparation, like disulfide bond reduction and alkylation, enzymatic *N*-glycan release, and analytical separation time, were optimized and reduced. The total length of the sample preparation protocol was reduced from 16 h when performed manually to 60 min when performed on the robotized platform. Bladergroen et al. were the first to integrate a complete robotized and HT sample preparation workflow for the MALDI-MS-based analysis of *N*-glycans released from total plasma (Figure 10).^193^ Minute amounts (6 μL) of total plasma were denatured using SDS, and *N*-glycans were released using PNGase F in a 96-well plate. Following enzymatic digestion, the sample preparation platform, based on the Hamilton MicrolabSTAR robot system, performed sialic acid derivatization through ethyl esterification, HILIC-SPE *N*-glycan purification, premixing of the purified and derivatized *N*-glycans with super-DHB matrix (9:1 (*w*/*w*) mixture of DHB and 2-hydroxy-5-methoxybenzoic acid) and sodium hydroxide, and subsequent spotting on a MALDI target plate with a 384-well format. The nature of the setup allowed for parallel processing of 384 samples in approximately seven hours (excluding *N*-glycan release), which comes close to an effective one minute per sample.

Alternative slide-based strategy to directly profile *N*-glycans from serum and plasma samples has been recently developed by Blaschke et al.^195^ The approach consists of 1 up to 2 μL of serum/plasma spotting on an amine-reactive slide, delipidation, and desalting steps followed by PNGase F deglycosylation by spraying the enzyme directly on the slide and analysis with MALDI-FTICR-MS. Equivalent approach based on MALDI-MS imaging (MSI) can also be used to profile *N*-glycome of more complex samples, e.g., urine, prostatic fluids, and expressed prostatic secretions.^196^ Although this novel rapid workflow has not yet been applied on a larger sample set, the approach does not require steps of glycan labeling, derivatization, and/or purification, making it attractive for future HT clinical applications.

#### 5.3.2 Measurement and Data Processing

Although it was established early on that the introduction of MS in HT glycomics shifted the limits in throughput from analysis to sample workup and data processing, there have been substantial advances in (the use of) MS instrumentation that have pronounced effects on throughput. There are several ways of improving MS-related throughput: (i) directly through increasing the speed of the MS analysis/detection and (ii) indirectly through increasing the sensitivity of the MS detection and thus requiring less time for sampling.

The biggest impact for MALDI-MS-based HT glycomics is the recent release of high-speed MALDI-time-of-flight (TOF)-MS platforms.^197−199^ Both the linear MALDI-TOF/TOF-MS and the orthogonal MALDI-quadrupoleTOF-MS platforms operate using high-speed MALDI-stages and UV lasers with a 10k Hz repetition rate. This makes them capable of analyzing samples approximately 1 order of magnitude quicker than the previous generation of MALDI-MS equipment typically equipped with 2k Hz lasers and slower MALDI-stages.

For high-resolving power MALDI-FTICR-MS-based detection of released *N*-glycans, the major throughput affecting development is the introduction of 2Ω FTICR detection.^200^ Commercialized by Bruker Daltonics, 2Ω detection FTICR offers the ability to record double the number of datapoints within the same transient length of a conventional FTICR analysis and thus providing superior mass resolving power in the same analysis time. However, it can also be exploited to record data at the resolving power of a conventional FTICR analysis, albeit with half the transient length, increasing throughput by a factor of two.

Another fundamental development that has the potential to increase throughput for HT glycomics studies is the introduction of laser-induced postionization following MALDI, also referred to as MALDI-2.^201,202^ In short, MALDI-2 results in enhanced ionization for a number of analyte classes (including released *N*-glycans) and is based on the illumination of the developing MALDI-plume with a secondary UV laser following material ablation and desorption with the primary MALDI laser. Explorative studies into the effects of MALDI-2 of (oligo)saccharides have shown predominant enhancement of the formation of deprotonated ions in the negative polarity. A similar development has surfaced for ESI-MS-based approaches, where the enhancement of both desolvation and ionization is achieved through the use of dopant-enriched nitrogen (DEN) gas.^139,203^ The positive effect of DEN gas on measurement sensitivity of released *N*-glycans has been shown for both negative and positive ionization, and both CE-ESI-MS and (n)LC-ESI-MS approaches.

Optimal signal processing for HT approaches occurs on-the-fly, and generally data acquisition software has basic on-board signal processing capabilities like smoothing, baseline subtraction and recalibration. Nevertheless, reprocessing data or processing data offline can be valuable to have additional control over processing steps and parameters. Also, additional quality control measures are implanted which can aid in automatically removing outlier spectra or features. Although not specific for HT glycomics data, GeenaR is an open-source and publicly available R-package that performs spectral preprocessing, quality control, feature extraction, and spectral clustering for MALDI-TOF-MS spectra at substantial speed.^204^ The full GeenaR workflow takes approximately 6 s per spectrum, which is negligible when put in perspective of the time spent per sample on sample preparation, which is in the order of minutes. Important for HT clinical studies is the availability of a quality control module that identifies potential outliers and which takes into account the number of *m*/*z* values per spectrum in relation to the mass resolution, the intensity range of the *m*/*z* values, the presence of null or empty spectra, and resolution irregularities. Every processed spectrum is scored, and the user can decide in the postprocessing to discard (potential) outliers. Another open source solution to perform spectral outlier detection is the R-package MALDIrppa.^205^ The package assesses the multipeak structure of a MALDI-TOF-MS spectrum and provides it with an “atypical” (A-)score. The user can define or calculate A-score tolerances to identify outliers. Through removing outlier spectra prior to processing, precious time can be saved by only processing high-quality spectra.

#### 5.3.3 Peak Assignment

The annotation of *N*-glycan compositions to *m*/*z* features is historically performed manually and based on accurate mass and isotopic pattern matching, following basic knowledge on *N-*glycan biosynthetic pathways. However, there has been a substantial effort in automating the peak annotation from MALDI-TOF-MS spectra to make it more reliable and faster. Cartoonist is a software tool that assigns *m*/*z* values to what the authors refer to as “cartoons” or glycan monosaccharide compositions.^35,206^ It includes the annotation of isomeric variants, although because the software works with MALDI-MS spectra, it cannot differentiate between the isomeric species.^35,206^ The mGIA method,^207^ and its improved version called GlycoMaid,^208^ published by Xu et al., are based on the matching of glycan isotope abundances to a previously constructed theoretical database. Uniquely, GlycoMaid is set up to deal with overlapping and isobaric glycan compositions and deconvolute the contribution of the individual *N*-glycan species to the measured isotopic distribution. *Toolbox Accelerating Glycomics* (TAG) is a tool for *m*/*z* annotation with *N*-glycan compositions and pathway analysis of MALDI-TOF-MS data.^209^

### 5.4 Mass Spectrometry of Glycopeptides

**Figure 11:**
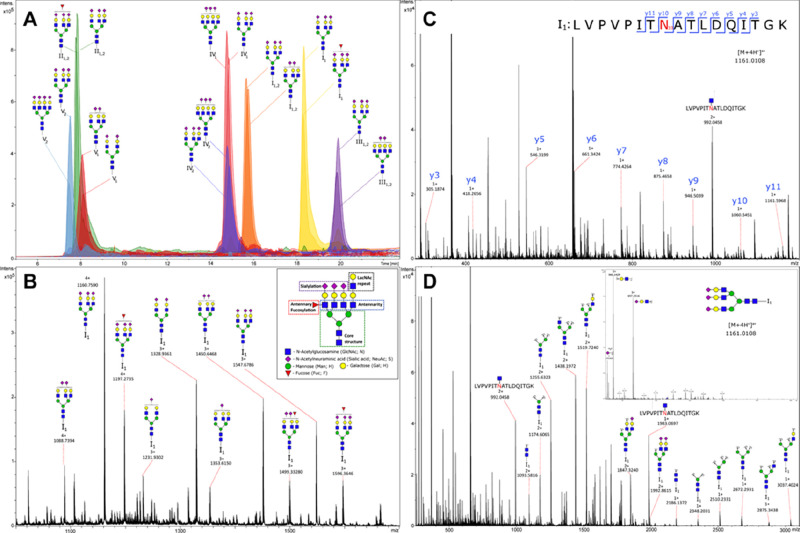
Results of the HT and site-specific *N*-glycosylation LC-MS analysis of human AGP. (A) Typical chromatogram with extracted ion traces of the most abundant glycopeptides from each glycosylation site. Trifluoroacetic acid was used in the mobile phase as an ion-pairing agent. (B) Summed mass spectrum for glycopeptide I1 with the most abundant glycan structures annotated. (C) MS/MS fragmentation spectrum for the peptide part of glycopeptide I1 with glycan composition N5H6S3. (D) MS/MS fragmentation spectrum for the glycan part of glycopeptide I1 N5H6S3. Reproduced with permission from ref (20). Copyright 2022 Keser et al. under the CC BY license (http://creativecommons.org/licenses/by/4.0/).

HT glycopeptide analysis has, hitherto, been largely focused on the analysis of IgG Fc glycopeptides, with recent expansions toward combined IgG and IgA glycosylation analysis^19,210^ as well as analysis of AGP (Figure 11).^20^ Fc glycopeptide analysis is achieved by LC-MS, with a few occasional MALDI-based analyses.^211,212^ RP-LC-MS is the main method used for HT IgG Fc glycopeptide profiling. LC-MS approaches for IgG glycosylation analysis at the glycopeptide level are already around for several decades, building on bottom-up proteomics workflows for the analysis of mAbs (peptide maps with MS detection) to monitor primary sequences of antibodies as well as a range of modifications including glycosylation.

#### 5.4.1 Sample Preparation

In regard to sample preparation, the use of 96-well plate methods has been key for achieving HT.^211,212^ Workflows tend to start with complex matrices such as blood serum or plasma. The most commonly used initial step is an affinity enrichment of IgG using, e.g., immobilized protein G or protein A, with an acidic elution step.^69,212,213^ The choice of a volatile acid, such as formic acid, allows retrieving the eluted IgG using vacuum centrifugation. Sample preparation is continued by tryptic cleavage, generating for human IgGs a glycosylated nonapeptide covering the conserved *N*-glycosylation site of the C_H_2 domain of the IgG heavy chain.^213^

The acid elution step followed by vacuum centrifugation appears to have an important role in preparing the immunoglobulins for tryptic digestion. Importantly, this acid treatment appears sufficient to prime the antibody for the generation of tryptic Fc glycopeptides, without the need for reduction and alkylation steps.^214^ Tryptic cleavage can be achieved in low strength volatile buffer (e.g., 25 or 50 mM ammonium bicarbonate) by overnight incubation at 37 °C. The resulting IgG (glyco-)peptide mixtures can be directly used for LC-MS analysis but may also be stored for a longer period at −20 °C prior to MS analysis.^215^ Affinity purification with acidic elution has been successfully implemented not only for HT IgG glycoprofiling but also for other target glycoproteins such as IgA.^19,210^

In the past decade, HT IgG Fc glycosylation analysis has been expanded toward the analysis of antigen-specific IgG responses, such as autoantibodies, alloantibodies, antipathogen, and antivirus antibodies.^216−220^ For this, adaptations of enzyme-linked immunoassay (ELISA) procedures are most often applied: microtitration plates are coated with target antigens and used to capture the specific antibodies from serum or plasma, followed by extensive washes and elution of the adsorbed antibodies with an acidic solution. Further sample preparation is identical to that for affinity-purified, total IgG.

#### 5.4.2 Measurement and Data Processing

Generally, no glycopeptide enrichment is being applied, and the complete tryptic IgG digests are analyzed by RP-LC-MS. Hereby, retention of polyclonal human IgG Fc glycopeptides is governed by sequence variations in the peptide portion and differs between subclasses, providing distinct IgG Fc glycopeptide clusters for the IgG1, IgG4, and IgG2/3 subclasses.^221^ Depending on the specific IgG3 allotype, IgG2 and IgG3 can result in an identical mass for their Fc glycopeptide portion. Notably, dependent on the ethnicity, other IgG allotypes may be dominant, with identical Fc glycopeptide signals for the IgG3 and IgG4 subclasses.^221^

Signal intensity in IgG Fc glycopeptide analysis can be boosted by applying a DEN gas, which, in combination with nanoscale separation (nanoESI), allows detection of low attomole amounts of IgG Fc glycopeptides on-column.^213^ Of note, this sensitivity is not needed for analysis of total serum or plasma IgG as the concentrations are approximately 10 mg/mL. However, the high sensitivity detection pays off with regard to method robustness, as high-quality measurements can be obtained by using only minute amounts of material, thereby minimizing contamination of the LC system and the ionization source. The high sensitivity is a prerequisite for the analyses of various specific IgG responses, e.g., some autoantibodies^222^ or alloantibodies^219^ of low abundance. Both high-resolution and low-resolution MS detection have been successfully applied,^216,219^ with high resolution having particular advantages in reducing or avoiding interference from coeluting, nonglycopeptide signals.

Next to LC-MS, MALDI-MS approaches have been developed for IgG Fc glycopeptide profiling, with various HT applications.^211,212^ Of note, by applying relatively cold MALDI matrices, these studies succeeded in limiting sialic acid loss and successfully detected sialylated IgG Fc glycopeptides, albeit with a still prominent loss of part of the sialic acids. Further developments have been made to largely or even completely prevent sialic acid loss. First, a particularly cold MALDI matrix was implemented, in combination with an intermediate-pressure MALDI source on an FTICR-MS, which allowed rapid collisional cooling of the protonated glycopeptide ions.^165,223^ Second, linkage-specific sialic acid derivatization was established for IgG Fc glycopeptides, allowing the facile, robust detection of both sialylated and nonsialylated IgG Fc glycopeptide.^177^ Both methodological innovations have, to our knowledge, not yet found their way into HT glycopeptide profiling, with HT LC-MS glycopeptide analysis approaches clearly prevailing.

Commonly data processing is performed in a targeted manner in batch mode using a glycopeptide list of the IgG subclasses of interest.^224^ Data curation focuses on confirming analyte identity on the basis of accurate mass, retention time, and isotopic pattern. The latter is also used for assessing and excluding potential interferences for high-precision quantification.

#### 5.4.3 Peak Assignment

Analyte assignment is generally performed on the basis of accurate mass, retention time, and isotopic pattern using common glycobiological knowledge for a sanity check of the observed glycopeptide compositions and profiles. Analyte identities can be further confirmed for selected cases using tandem MS and released glycan analysis.^225,226^ With the increase of sensitivity and dynamic range of analytical methods, additional, minor glycopeptide variants such as hybrid-type glycans and monoantennary species are being included.^227^ The range of human IgG Fc glycans may be further increased with the detection of very low-abundant sulfated species, yet compelling evidence for their presence of the IgG Fc portion is still pending.^228,229^

### 5.5 Lectin Microarrays

Lectin microarrays was reported for the first time in 2005.^99−102^ The display of lectins in a microarray format enables multiple, distinct binding interactions to be observed simultaneously and therefore provides a complementary method for the HT screening of carbohydrates on glycoproteins or glycolipids.^230^ On the basis of the interaction and binding of the carbohydrate residues of the analyzed compounds with the corresponding lectins, lectin microarrays allow determining their specificity and the registration of interactions at very high accuracy, while the application of lectin microarrays in the analysis of hundreds or thousands of samples has been hurdled by the fact that they are mostly used for comparative analysis of glycan profiles (e.g., disease versus healthy) due to lack of quantitation, and rely on complementary techniques, most often MS,^106^ for the complete determination of glycan structures. However, in contrast to UHPLC and CE, which require the release of glycans from a glycoprotein, and MS, which mostly involves enzymatic digestion of a glycoprotein, lectin microarrays enable rapid and direct measurement of glycan profiles in complex biological samples, including *O*-glycans,^108,231^ on an intact glycoprotein^232^ and GSLs^233,234^ without the need for protein digestion and glycan release.

#### 5.5.1 Sample Preparation and Measurement

**Figure 12:**
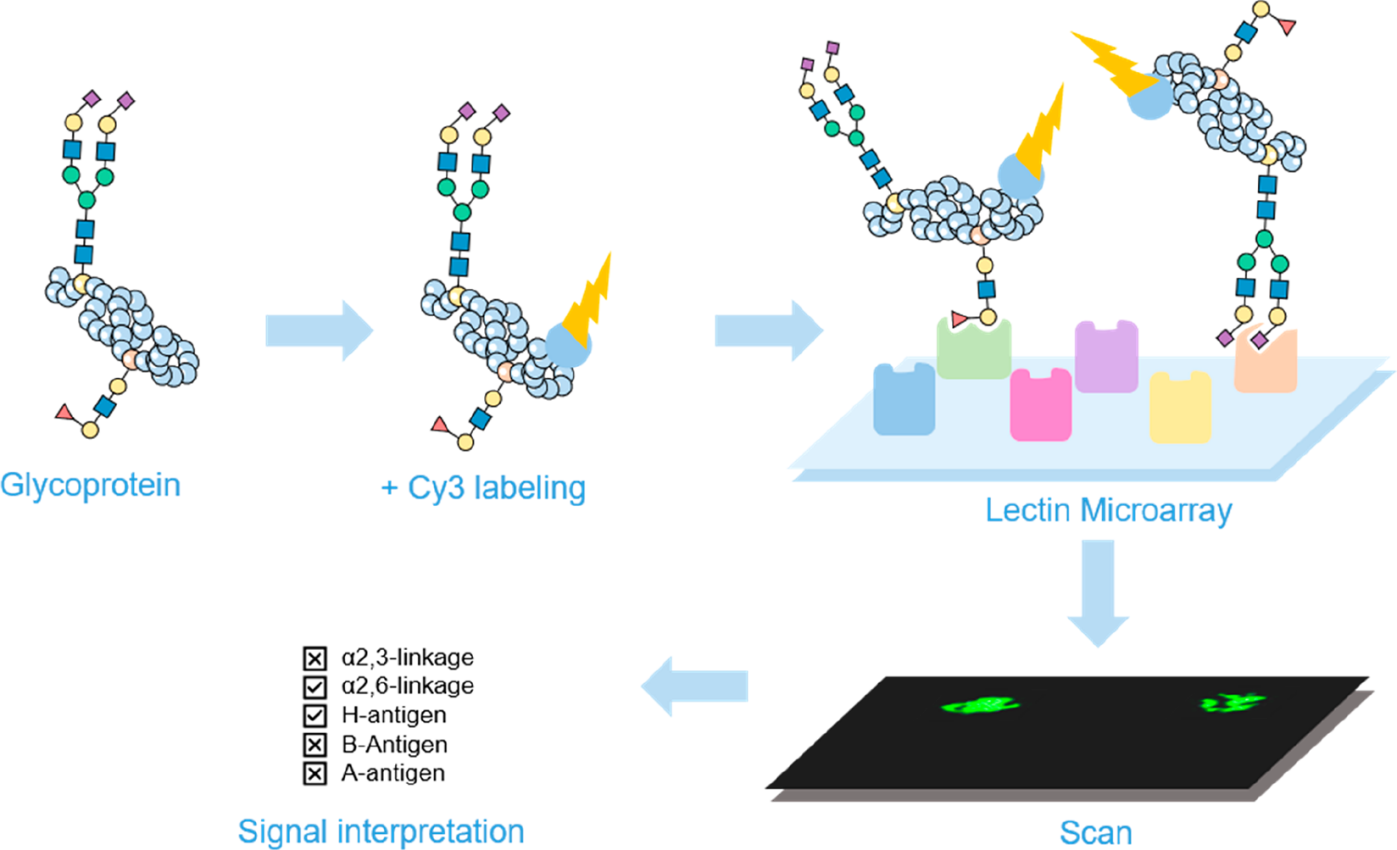
Glycan microarray analysis. A graphical representation of a glycan microarray workflow, the consecutive processing steps are as follows: (1) glycoprotein is labeled with a fluorescent tag through Cy3 labeling, (2) the glycoprotein binds at the lectin microarray based upon the presented lectin on the microarray, and (3) the microarray is scanned, followed by (4) the interpretation of the signals.

Lectins with known and overlapping binding specificity are immobilized as microdots on a solid, usually glass, surface which has previously been activated by biochemical (e.g., streptavidin) or chemical (e.g., epoxy, *N*-hydroxysuccinimide (NHS), amino, gold) derivatization. After lectin immobilization, residual activated groups are blocked (e.g., using glycan-free serum albumin or amine). Interaction of carbohydrate residues with the corresponding lectins can be detected either directly through their prior labeling with fluorescent reagents (e.g., Cy3 monoreactive dye)^106,235^ or indirectly (e.g., by overlaying a fluorescently labeled antibody (if available))^103^ against the target glycoprotein (approach termed antibody-overlay lectin microarray) (Figure 12). Direct labeling is often preferred, however, it requires a relatively high amount of glycoproteins, it can disrupt glycoprotein–lectin interactions and it suffers from low sensitivity. On the other hand, indirect detection of glycan–lectin interactions using tyramide signal amplification for antibody-overlay lectin microarray (TSA-ALM), developed by Meany et al. in 2011 enabled detection of weak glycan–lectin interactions as a result of 100-fold increased sensitivity by using biotinylated tyramide.^104^ Indirect labeling, therefore, offers increased sensitivity due to signal amplification as well as high specificity due to low background labeling.^105^ Specific lectin–glycan interactions are detected with high accuracy after washing off the unbound probe^98^ to obtain the characteristic “glycan fingerprint”.

The detection of interactions can be achieved using confocal and nonconfocal fluorescence^98^ and evanescent-field activated fluorescence.^98,101,148^ A bimolecular fluorescence quenching and recovery detection do not require the preliminary labeling of target carbohydrates.^236^

#### 5.5.2 Data Processing/Peak Assignment/Identification

Fluorescent intensities of each lectin–glycoconjugate spot are usually extracted using appropriate software, e.g., Gene Pix,^106^ Mapix,^231^ GlycoStation ToolsPro,^237^ etc. Generally, the background is subtracted and signal values less than average background + two standard deviations are excluded from further analysis to eliminate the influence of nonspecific adsorption.^106^ Global normalization (to obtain normalized fluorescent intensities; NFIs) is further used to eliminate fluorescence bias, and processed data of the parallel data sets are compared with each other based on fold-changes. Normalized data is often analyzed using unsupervised average hierarchical cluster analysis (HCA) and principal component analysis (PCA).^106^

Automated data analysis for lectin microarray technology has been reported already back in 2007 by Rosenfeld et al., who developed an algorithm for glycan fingerprint interpretation based on the data obtained by analyzing hundreds of fingerprints.^103^ The fingerprint, a histogram representing the intensity of lectin–glycan binding, is finally represented as a table of glycan structures found in the sample and their relative abundances.

Identification of glycan epitopes largely depends on the repertoire of lectins used for microarray preparation. Historically, these have mostly been plant lectins whose specificity does not cover all human glycosyl epitopes and could lead to erroneous interpretation. Discovery and biochemical characterization of other natural lectins, as well as recombinant development of new lectins with more narrow specificity, can aid in obtaining more detailed information on structural glycan features facilitating their use in glycomic applications. Lectin microarrays have been successfully applied for glycan profiling of complex protein samples in highly heterogeneous matrices and could, in the future, find their application niche in cases where sample processing for detection with other techniques, e.g., MS, would be extremely laborious and complex. This approach could be useful for rapid assessment of the physiological status of cells or tissues by profiling cell surface carbohydrate expression patterns.

### 5.6 Data (Pre-)Processing and Analysis

**Figure 13:**
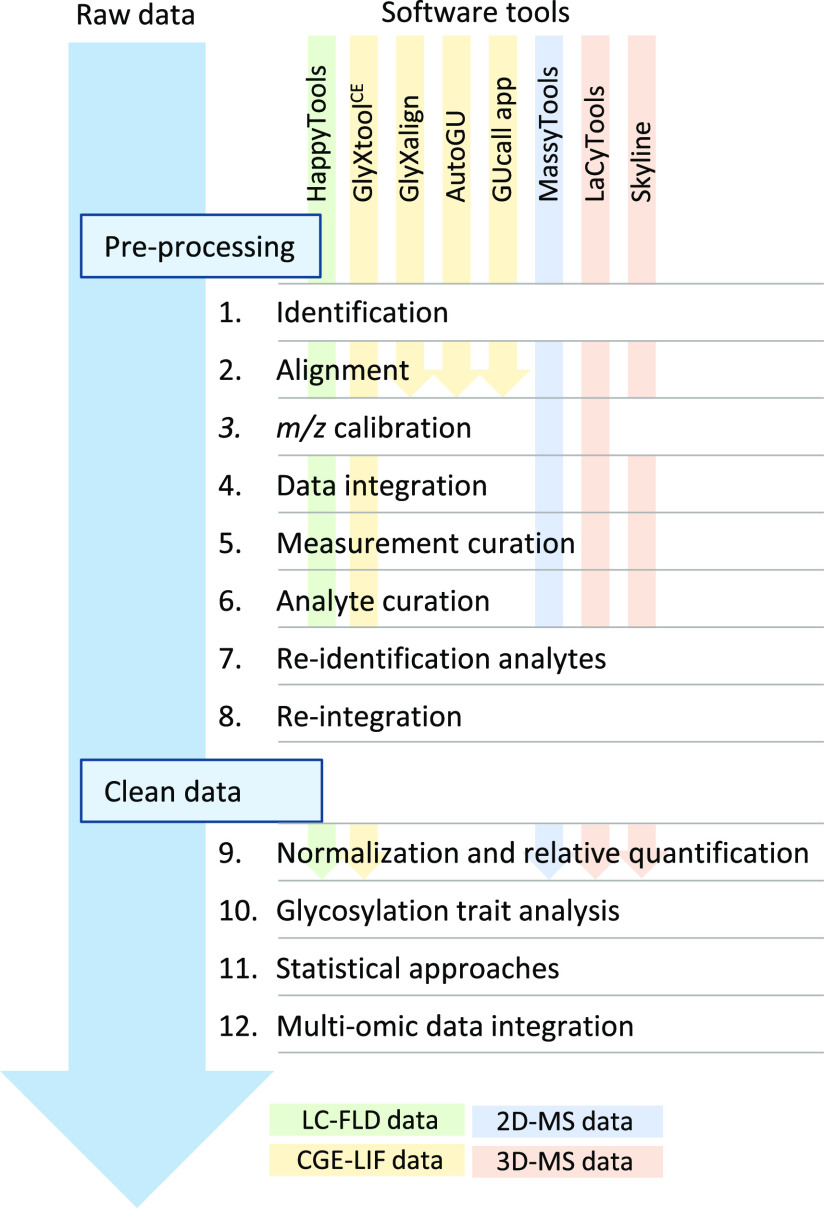
Workflow for data (pre)processing and analysis. The various steps (1–8) involve preprocessing of raw data into clean data. Eventually, the data is normalized, and each analyte will be relatively quantified based upon the total summed area of all analytes observed in a measurement (%area), also known as direct glycosylation traits. To obtain insights in the biosynthetic pathway specific glycosylation features can be summed (e.g., galactosylation, sialylation, fucosylation). Either the direct or derived glycosylation traits can be used for statistical evaluation and the whole data set can be integrated with other data sets (e.g., proteomics or genomics).

In recent years, various computational tools have been developed to allow a faster and more user-friendly processing of HT glycomics data, e.g., for the identification of potential glycan biomarkers. Nonetheless, data processing still presents a major bottleneck in HT glycomics. Typically, HT glycomics data undergo various preprocessing steps as well as pretreatment steps before they can be used for data analysis as detailed in the following sections (Figure 13).

#### 5.6.1 Identification

Most of the current HT glycomics data processing methods are designed to target specific sets of analytes and prior to data processing, the glycomic analytes present in the sample should be explored and identified by employing strategies described earlier for the different HT glycomic technologies (see sections 5.1–5.5).^167^

Dependent on the applied technique, various libraries, tools, and databases are available to identify the glycan compositions based upon (normalized) retention time (HPLC-FLD; GlycoStore, previously GlycoBase),^238^ migration time (CGE-LIF; GlycoStore^239^ and glyXbase),^154^ accurate mass (MS; GlycoWorkbench, GlycanMass, UniCarb-DB (MS/MS), GlycoStore,^239^ and GlycoMod) or a combination of retention time and accurate mass data (UHPLC-FLR-MS, UNIFI).^240^ An extensive overview of available databases and repositories is provided in Table 1 and described more comprehensively in a recent review.^241^ Analyte identification can be supported by exoglycosidase treatments as well as the use of internal and external standards. For MS-based data, additional MS/MS experiments can be performed to further gain confidence in the glycan structure. For the analysis of released glycans, which are often analyzed by negative mode MS/MS, spectral libraries are available (Table 1),^242^ but often manual interpretation and identification is required which can, for example, be supported by GlycoWorkbench.^243^ Various software tools are available for glycoproteomics data (e.g., GlycoForest^244^ and Byonic; Table 1). However, also here manual interpretation and verification are recommended to contain the risk of false automatic assignments by the software tool. In cases where only accurate MS data is available, analytes can be assigned based on differences in retention time as well as mass increments relative to confidently identified glycans using tools such as GlycoMod,^245^ GlycoWorkbench,^243^ and GlycopeptideGraphMS^246^ (Table 1). It should be noted that current MS HT glycoproteomic approaches mainly provide monosaccharide compositional data and isomer differentiation is not achieved (except when sialic acid derivatization is employed).

#### 5.6.2 Data Pre-Processing

Prior to data extraction, the raw data should be transformed into clean data and during this procedure often corrections are performed such as baseline correction or an alignment. This is to avoid any technical variation introduced by the analytical method that has been used and to match peaks across the sample set. Various software tools are available to perform these preprocessing steps in batches (Figure 13 and Table 1).

##### 5.6.2.1 Alignment and Calibration

To ensure precision and minimize day-to-day and other potential technical variations introduced by the analytical platform, the first step for raw chromatographic and electrophoretic data involves the conversion of the observed retention/migration time to relative retention/migration times. For multiplexed CGE-LIF based data, an alignment algorithm is featured in glyXalign that identifies distinctive data points in the electropherogram.^247^ In addition, the alignment procedure can be followed live with an intuitive graphical user interface. Next to this tool, several tools are available that rely on the separate analysis of a glucose ladder across the sample set. Upon the basis of this ladder, the migration time can be standardized in GU. However, this calculation was, until a decade ago, a time-consuming task as no tools were available for the automatic assignment. This holdup disappeared with the release of the freely available tools such as the autoGU^238^ (recently replaced by GlycanAnalyzer)^248^ and the GUcal app.^153,249^

The introduction of a triple-internal standard (maltose, maltotriose, and maltopentadecaose; Agilent) avoids the need for additional measurements of a glucose ladder, as the internal standard can be coinjected with the samples.^250^ Here, the migration time will be transformed to a migration time unit (MTU), which will be assigned to each observed peak. However, caution is needed that the coinjected standards do not interfere with the analytes of interest (comigration), which could skew the MTU value. The commercially available software tool glyXtool^CE^ (glyXera)^251^ combines the coinjection of the triple-internal standard with the analysis of a GU ladder, resulting in a double migration time alignment (MTU′′).

Similar to glyXalign for CE-LIF data, a freely available computational tool called HappyTools, has been developed for LC-FLD data and allows automatic peak picking followed by the alignment of the retention times.^252^ While automated peak picking can be used, the user can also choose to provide a predefined list of analytes that will be used for peak picking and provides information about the peaks (e.g., retention time of the peak as well as the time window of the peak).

**Figure 14:**
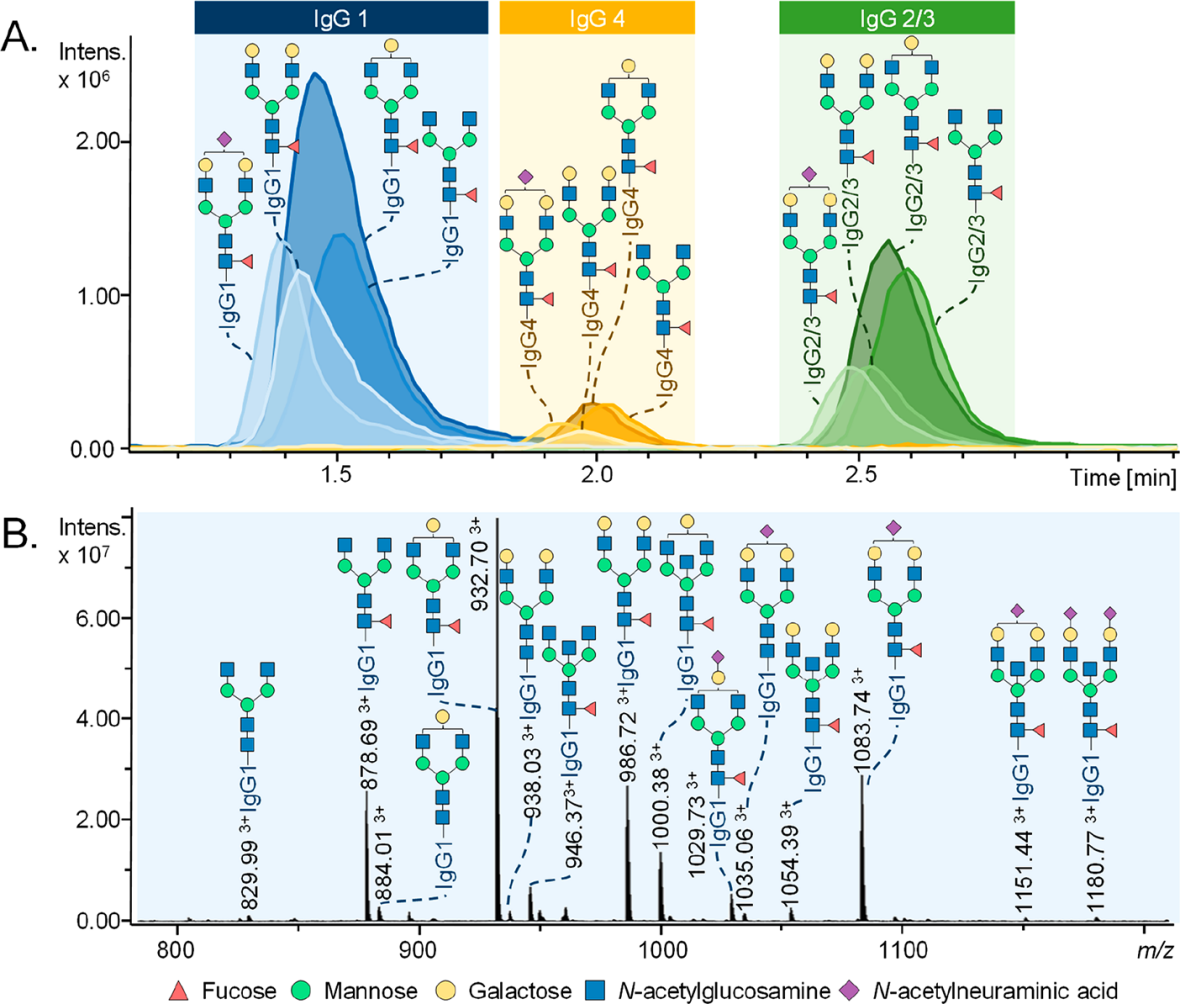
RP-LC-MS separation of tryptic Fc glycopeptides of a polyclonal serum IgG standard. (A) Extracted ion chromatograms (EIC) of the most abundant Fc glycopeptides per IgG subclass from polyclonal human serum IgG. The separation is mainly driven by the hydrophobicity of the peptide backbone and separate clusters are obtained where all glycoforms of a specific subclass elute. (B) Illustration of a summed mass spectrum of the cluster containing IgG Fc glycopeptides from subclass IgG1. Only the mass range of the triply charged species is displayed. IgG1 = EEQYNSTYR; IgG2/3 = EEQFNSTFR; IgG4 = EEQFNSTYR.

In the case of 2D MS-related data, MassyTools is capable of performing the calibration of the data when prior knowledge is available.^253^ Namely, specific masses need to be provided to perform the calibration of the spectra. Ideally, internal standards which cover the full *m*/*z* range can be used for this process. However, in most cases, predetermined analytes are selected that are known to be abundant in the samples as well as being previously identified by other techniques (e.g., tandem MS and exoglycosidase treatment) and a minimum of three *m*/*z* values should be provided. The alignment is also an important aspect for 3D MS data, where time is added as a third dimension (when compared to 2D MS data). For this purpose, other software tools are available such as LaCyTools, which is a targeted data processing package.^224^ Compared to MassyTools, there is no automated peak picking and prior knowledge is required. An alignment file should be provided that contains a list of masses with the desired/expected migration or retention times. For an appropriate alignment, at least three features with a unique retention time are required, preferably with a retention time covering the time window of interest. The alignment can then be followed up by a calibration step similar to that of MassyTools. However, this will not be performed on the total data set but only on those for specific clusters that will be predefined in the reference list. This list will contain all the analytes that should be integrated, and, depending on the separation technique, specific clusters can be defined. For example, RP-LC-MS analysis of IgG glycopeptides results in the (almost) coelution of the glycopeptides from the same IgG isoforms, as the separation is defined by the peptide portion (IgG subclass) and not so much by the glycan.^213^ This results in three distinct clusters of glycopeptides, which can be defined in the reference list with a specific retention time (± retention time range). It is important to note that, based upon each unique retention time and corresponding time window, LaCyTools will create 2D data (Figure 14B) from the 3D data by summing all spectra in the defined range per cluster (Figure 14A). The analytes that should be used for calibration can be selected in the last column of the reference list. However, please note that, if various clusters are defined, each cluster should contain at least three analytes that can be used for calibration. This calibration step could be considered a drawback of the software tool as, dependent on the analysis, not all clusters might have enough (confirmed) analytes that could be used for calibration.

Exclusively for use with MALDI-MS data, rMSIproc is an open-source and publicly available R-package for spectral preprocessing and feature extraction.^254^ Although the package was developed to work with MALDI-MSI data (Bruker XMASS, imzML, and.tar formats), its open-source nature allows its application to XY-type files. The spectral preprocessing features Savitzky–Golay smoothing, label-free spectral alignment, recalibration of *m*/*z* values, peak picking, intensity normalization (to either total ion current/count (TIC), or using the root-means-squared (RMS) method) and signal-to-noise (S/N) ratio threshold peak picking. All processing can be applied to individual spectra, as well as batches of multiple spectra using the merged processing feature. The latter option provides a merged peak matrix, providing the intensities of all individual spectra projected on a common *m*/*z* axis.

To allow a faster identification process for HT glycomic data sets, new analytes can be identified by using a sample pool of analytes per investigated group (e.g., patients versus healthy controls) using exoglycosidase treatments or MS to assign unidentified peaks or *m*/*z* values. If new features are observed, it will be explored if these are indeed potential new analytes that can be added to the analytes/reference list by consulting the databases (Table 1).

##### 5.6.2.2 Data Integration

A list of target analytes can be established, based upon the previous step, and used for the integration of the glycans or glycopeptides. While in some cases manual integration is necessary, to enable a HT glycomic workflow, several software tools can be considered for automated integration. Common tools are HappyTools for LC-FLD, glyXtool^CE^ for CGE-LIF, MassyTools for 2D MS data, and LaCyTools or Skyline for 3D MS data, and all make use of a target list and have the ability to subtract the background. For complex samples, more sophisticated approaches based on machine learning are also being developed.^79^

Whereas LaCyTools can be considered a bit more of a black box, Skyline allows visual interpretation of the automatically integrated data by using EICs/electropherograms.^255^ Moreover, it allows to assess the data quality, adjust the peak integration, and supports the raw data formats from almost every MS vendor (AB SCIEX, Agilent, Bruker, Thermo, and Waters), while LaCyTools requires a more generic form (mzXML). In the case of isomeric separation, caution is needed using LaCyTools. Namely, when the isomers are baseline resolved separate clusters can be created and can be integrated. If this is not the case and both isomers fall within the same time window of the cluster, the isomers will be considered the same analyte due to the same *m*/*z* value and integrated in such a way. Or if the time window is not set properly, only a part of an isomer might be taken into account for the integration.

##### 5.6.2.3 Data Curation

To evaluate whether the individual analytes or measurements should be included for further processing, a (semi)automatic curation can be performed. Several parameters can be used for this; for example, HappyTools provides information about the observed retention time versus the expected retention time, observed S/N for each peak, and how well the observed peak fits with a Gaussian peak. Recently, an enhanced semiautomated computational approach based on electropherograms clustering, manual peak curation, and the HappyTools has also been reported.^256^ Both LaCyTools and Skyline provide valuable information about the integrated analytes such as S/N, ppm error, and the similarity between the observed isotopic pattern of each analyte (per charge state) versus the theoretical isotopic distribution. Specific values can be set for each parameter to decide whether the observed feature can be assigned as the expected analyte or that it might be an isobaric interference and that it should be removed from the data set. The same accounts for the measurement, when a large fraction of analytes is observed with an overall low S/N, it should be evaluated whether the specific measurement should be excluded from the data set.

It is recommended to upload the curated assigned chromatographic, electrophoretic, and mass spectrometric compositional data on GlycoStore (www.glycostore.org) to further support the resource that facilitates detailed glycan analysis for different workflows, or on their designated repositories (e.g., GlyTouCan; Table 1).

#### 5.6.3 Normalization and Relative Quantification

Eventually, the final relative quantification of the detected glycoforms can be obtained, after the data preprocessing steps, by normalizing the data based upon the total area of all observed analytes (per protein and site, if applicable). This will result in %area, which will be further processed to assess whether a batch correction is needed and, eventually, to perform statistical analysis. Relative quantification of glycans remains the most widely used approach because it is more time- and cost-effective compared to absolute quantification, offering at the same time insights into aberrant glycan synthesis and regulation of the glycosylation process. On the other hand, absolute quantification may have an application niche in clinical diagnostics of free oligosaccharides, e.g., in milk or urine, as well as for glycosylation analysis of therapeutic glycoproteins.^257^ However, it should be noted that absolute quantification is not yet applied for HT glycomic workflows, even though advancements have been made in the CE field by the usage of an APTS labeled standard^149^ and in the MS field by the usage of isotopically labeled standard^258^ and labels.^126,259^

#### 5.6.4 Glycosylation Trait Analysis

Directly measured glycosylation traits depend on the used technology and are generally individual fluorescently labeled glycan structures or individual glycopeptides that have been efficiently detected and quantified by UHPLC/CGE/MALDI analysis or LC-MS analysis, respectively. Even when the same quantification technology is used, differences in sample preparation protocols or analysis conditions affect separation (in the case of UHPLC and CGE) or ionization (in the case of MALDI and LC-MS) and can result in differentially quantified glycan structures. To enable integration of different studies and/or measurements, derived traits based on the shared glycan structural features (e.g., sialylation, galactosylation, fucosylation, branching, etc.) are being calculated from directly measured glycan species. Derived glycosylation traits are biologically more related to activities of specific enzymes in the glycosylation pathway and underlying genetic polymorphisms, making them beneficial for understanding the functional relevance of obtained results as well as for GWAS.

#### 5.6.5 Data Analysis

Glycan measurements are not normally distributed (right-skewed distribution) and are usually log-transformed after normalization. Batch correction is then performed on log-transformed measurements by modeling the technical source of variation (which sample was analyzed on which plate) as a batch covariate.^260^ To correct the measurements for experimental noise, estimated batch effects are subtracted from log-transformed measurements and obtained results used for further statistical analysis. Because of the analysis of multiple (direct and/or derived) glycosylation traits correction of *p*-values for multiple testing should be applied.

#### 5.6.6 Multi-Omic Data Integration

One of the challenges of vast glycomics data generation and integration with databases, is its complexity in terms of nonuniform glycan traits annotation that usually depends on the level of profiling, used analytical technology, originating glycoconjugates, etc. Although data generated by different technologies can be integrated, the same has been hindered by a lack of a digital standard to represent glycoproteins, consequently often leaving existing databases unpopulated with newly generated data.

Recent efforts of the global glycoinformatics community, through The GlySpace Alliance^261^ are directed toward providing relevant, trustable, and quality information regarding the glycan structures, their origin, biosynthesis, regulation, and functional roles. In line with that, the Minimum Information Required for A Glycomics Experiment (MIRAGE) commission aims to improve the quality of the glycomic data in the literature by implementing guidelines (e.g., sample preparation, glycan microarray analysis, LC, CE, and MS analysis)^262−266^ and templates that ensure that the most important details of a glycomic study are provided in their publications, making sure that these reported experiments can be repeated by others. Several journals now require these MIRAGE guidelines prior to submission of a manuscript. Whereas a standard semantic framework, GlycoConjugate Ontology (GlycoCoO), has been developed, solely on describing and representing glycoproteomics data.^267^

Efficient data integration is an essential prerequisite for some applications of HT glycomics, e.g., GWAS analysis, because they rely on the use of multiple cohorts data sometimes even generated with different technologies (e.g., UHPLC and LC-MS).^268^

## 6 Applications

The ability to reliably detect interindividual differences at the molecular level is the prerequisite of personalized medicine. Glycome composition integrates genetic, epigenetic, and environmental factors into chemical structures that can be reliably quantified, which makes it an ideal biomarker for personalized medicine.^269^ However, large studies need to be performed to properly evaluate and validate the biomarker potential of glycans. The total number of glycomic analyses is still very low if compared to the number of genetic, epigenetic, transcriptomic, metabolomic, or microbiome analyses, but thanks to the novel analytical approaches, more and more glycome data is being generated (Table 2).

**Table 2:** High-Throughput Glycomic Studies Involving More than 500 Samples^a^

cohort description	sample no.	technology	manual or automated	data processing tool	ref
**IgG**					
RA	1099	MALDI-MS	manual	flexAnalysis	van de Geijn et al. (2009)^212^
population study	2298	UHPLC-FLD, MALDI-MS, LC-MS	manual	GlycoWorkbench	Pucić et al. (2011)*^69^
population study	1709	MALDI-MS	manual	flexAnalysis	Pučić Baković et al. (2013)^211^
population study	5117	UHPLC-FLD	manual	NA	Krištić et al. (2013)^70^
GWAS	4095	UHPLC-FLD, MALDI-MS	manual	NA	Lauc et al. (2013)*^71^
population study, genetics and epigenetics	1050	UHPLC-FLD	manual	NA	Menni, Keser et al. (2013)^270^
RA	1800	LC-MS	manual	Xtractor2D	Bondt et al. (2013)^271^
population study, GWAS	1201	UHPLC-FLD, CGE-LIF, MALDI-MS, LC-MS	manual	flexAnalysis, DataAnalysis, Xtractor2D, glyXtool, glyXalign	Huffman et al. (2014)^167^
population study	3515	UHPLC-FLD	manual	NA	Nikolac Perkovic et al. (2014)^272^
SLE	1020	UHPLC-FLD	manual	NA	Vučković et al. (2015)*^273^
IBD	1114	UHPLC-FLD	manual	NA	Trbojević-Akmačić et al. (2015)*^274^
population study	701	UHPLC-FLD	manual	NA	Yu et al. (2016)^275^
allergic diseases	893	LC-MS	manual	NA	Pezer et al. (2016)^276^
CRC	1298	UHPLC-FLD	manual	NA	Vučković et al. (2016)*^277^
kidney disfunction	3274	UHPLC-FLD	manual	NA	Barrios et al. (2016)^278^
low back pain	4511	UHPLC-FLD	manual	NA	Freidin et al. (2016)^279^
hypertension	4757	UHPLC-FLD	manual	NA	Wang et al. (2016)^280^
population study	1826	LC-MS	manual	Xtractor 2D	Plomp et al. (2017)^281^
population study, role of hormones	1010	HPLC-FLD, UPLC-FLD	manual	NA	Ercan et al. (2017)^282^
glycosylation regulation	5243	LC-MS	manual	DataAnalysis, Xtractor2D	Benedetti et al. (2017)^283^
GWAS	8129	UHPLC-FLD	manual	NA	Shen et al. (2017)^284^
T2D	5984	UHPLC-FLD	manual	NA	Lemmers et al. (2017)^285^
statin therapy	4009	UHPLC-FLD	manual	NA	Keser et al. (2017)^286^
dyslipidaemia	598	UHPLC-FLD	manual	NA	Liu et al. (2018)^287^
hypertension	630	LC-MS	manual	NA	Liu et al. (2018)^288^
IBD	3441	LC-MS	manual	LaCyTools	Šimurina, de Haan, Vučković et al. (2018)*^289^
population study	637	UHPLC-FLD	manual	NA	Russell et al. (2019)*^74^
hyperuricemia	635	UHPLC-FLD	manual	NA	Hou et al. (2019)^290^
cardiometabolic diseases	701	UHPLC-FLD	manual	NA	Wang et al. (2019)^291^
T2D	849	UHPLC-FLD	manual	NA	Li et al. (2019)^292^
GWAS	12320	UHPLC-FLD, LC-MS	manual	NA	Klarić et al. (2020)*^268^
T2D	1112	UHPLC-FLD	manual	NA	Wu et al. (2020)^293^
T2D	1815	UHPLC-FLD	manual	NA	Singh et al. (2020)^31^
thyroid diseases	4636	UHPLC	manual	NA	Martin et al. (2020)^77^
population study	13061	UHPLC-FLD, LC-MS	manual	NA	Štambuk et al. (2020)*^29^
population study, weight intervention	3841	UHPLC-FLD	manual	NA	Greto et al. (2021)*^294^
hypertension	3452	UHPLC-FLD	manual	NA	Kifer et al. (2021)*^295^
hypertension and T2D	883	UHPLC-FLD	manual	NA	Meng et al. (2021)^296^
perimenopause	5080	UHPLC-FLD	manual	NA	Deriš et al. (2022)^297^
**Total Serum Proteins**					
population study	594	CE-LIF	manual	GeneMapper	Vanhooren et al. (2010)^298^
hypertension	972	CE-LIF	manual	Genescan	Vilar-Bergua et al. (2015)^299^
T2D	1161	CE-LIF	manual	Genescan	Testa et al. (2015)^300^
breast cancer	585	UHPLC-FLD	automated	NA	Saldova et al. (2017)^301^
RA	1562	MALDI-MS	manual and automated	flexAnalysis, MassyTools, GlycoWorkbench	Reiding et al. (2018)*^302^
**Total Plasma Proteins**					
population study	1008	HPLC-FLD	manual	GlycoBase	Knežević et al. (2009)* ^27^
population study	1914	HPLC-FLD	manual	GlycoBase	Knežević et al. (2010)^28^
GWAS	2705	HPLC-FLD	manual	GlycoBase	Lauc et al. (2010)*^118^
population study, lipidomics	2035	HPLC-FLD	manual	NA	Igl, Polašek et al. (2011)^303^
GWAS	3533	HPLC-FLD	manual	GlycoBase	Huffman et al. (2011)*^119^
metabolic syndrome	732	HPLC-FLD	manual	GlycoBase	Lu et al. (2011)^304^
ADHD and autism	525	HPLC-FLD	manual	NA	Pivac et al. (2011)^305^
medication	2360	HPLC-FLD	manual	NA	Saldova et al. (2012)^306^
HNF1A-MODY	783	HPLC-FLD	manual	GlycoBase	Thanabalasingham et al. (2013)^307^
population study	2144	MALDI-MS	manual	DataAnalysis, GlycoWorkbench	Reiding et al. (2017)^302^
GWAS	1338	UHPLC-FLD	manual	NA	Suhre et al. (2017)^308^
T2D	505	UHPLC-FLD	manual	NA	Adua et al. (2017)^309^
T2D	1614	UHPLC-FLD	manual	NA	Keser et al. (2017)^310^
T2D	2281	MALDI-MS	automated	flexAnalysis, MassyTools	Dotz et al. (2018)^311^
IBD	3631	MALDI-MS	manual and automated	MassyTools	Clerc, Novokmet, Dotz et al. (2018)*^312^
HNF1A-MODY	989	UHPLC-FLD	manual	NA	Juszczak et al. (2018)*^78^
CRC	1231	UHPLC-FLD	manual and automated	GlycoBase	Doherty et al. (2018)*^313^
GWAS	3811	UHPLC-FLD	manual	NA	Sharapov et al. (2019)*^314^
population study	2396	HPLC-FLD	manual	Matlab	Ruhaak et al. (2020)^315^
metformin and statin use in T2D	3333	MALDI-MS	automated	DataAnalysis	Singh et al. (2020)^31^
cardiometabolic risk	3140	UHPLC-FLD	manual	NA	Wittenbecher et al. (2020)^76^
GWAS	4802	UHPLC-FLD	manual	NA	Sharapov et al. (2021)*^316^
T2D	506	UHPLC-FLD	manual	NA	Edua et al. (2021)^317^
diabetes	610	UHPLC-FLD	manual	GlycoStore	Cvetko et al. (2021)^318^
T2D complications	3333	MALDI-MS, FTICR-MS	manual and automated	MassyTools	Memarian et al. (2021)^319^
**IgG and Total Plasma Proteins**					
kidney disease in T1D	818	UHPLC-FLD	manual	NA	Bermingham et al. (2017)^75^
evaluation of glycomics normalization methods	5139	UHPLC-FLD, MALDI-MS, LC-MS	manual	DataAnalysis, GlycoWorkbench	Benedetti et al. (2020)^133^
age-related macular degeneration	2835	HPLC-FLD, UHPLC-FLD	manual	NA	Bućan et al. (2022)^320^
**IgA**					
RA	1800	MALDI-MS	manual	MassyTools	Bondt et al. (2017)^321^
**Antigen-Specific IgG**					
RA	703	LC-MS	manual	LaCyTools	Bondt et al. (2018)^222^
COVID-19	650	LC-MS	manual	LaCyTools	Pongracz et al. (2021)*^216^
viral infections	2 × 400	LC-MS	manual	Skyline	Larsen et al. (2021)^217^
**Apolipoprotein CIII**					
population study	771	MALDI-MS	automated	MassyTools	Demus et al. (2021)^322^
**HMOs**					
infant morbidity and inflammation	659	LC-MS	manual	MassHunter Qualitative Analysis	Jorgensen et al. (2021)*^323^
**AGP**					
diabetes	635	LC-MS	manual	LaCyTools	Tijardović et al. (2022)^324^
**Tf and IgG**					
GWAS	1900	UHPLC-FLD	manual	NA	Landini et al. (2022)*^325^

a Studies denoted with an asterisk were highlighted in the main body of the manuscript. RA, rheumatoid arthritis; SLE, systemic lupus erythematosus; IBD, inflammatory bowel disease; CRC, colorectal cancer; T2D, type 2 diabetes; ADHD, attention-deficit hyperactivity disorder; HNF1A-MODY, hepatocyte nuclear factor 1α maturity-onset diabetes of the young; T1D, type 1 diabetes; COVID-19, coronavirus disease 2019.

### 6.1 HT-Glycomics in Epidemiological Studies

In 1988, Parekh et al. were the first to report on the age-dependence of IgG glycosylation by analyzing the degree of galactosylation of total serum IgG *N*-glycans (both Fab and Fc) in a group of 151 healthy individuals of both sexes aged 1–70 years.^38^ Any heterogeneity in terms of attached sialic acids, *N*-acetylglucosamine, and fucose was eliminated by appropriate exoglycosidase treatments prior to oligosaccharide analysis so that galactosylation was the only trait analyzed. Glycans were released, isolated, and radioactively labeled, as described previously by Parekh et al., in 1985 and Ashford et al., in 1987.^10,39^ In these studies, it was discovered that the mean incidence of agalactosylated *N*-glycans on IgG, in which both outer arms terminate in *N*-acetylglucosamine, is over 30% at birth, reaches a minimum of about 20% in individuals at age 25, and then increases steadily until it reaches about 40% at age 70. The mean incidence of monogalactosylated *N*-glycans remains impressively consistent at about 40% across the age range, whereas digalactosylated *N*-glycans show a parabolic pattern inverse to the parabolic age-dependence curve of agalactosylated *N*-glycans. There were no sex-specific differences found. The first large-scale study (>500 samples) of *N*-glycans was carried out on 1008 individual plasma samples by Knežević et al. in 2009^27^ and assessed whether there is any variability or heredity as well as key environmental factors that affect the human plasma *N*-glycome. The *N*-glycans from total plasma proteins were released and labeled with 2-AB following the procedure described by Royle et al.^53^ By combining HPLC analysis of fluorescently labeled glycans with sialidase digestion, glycans were separated and quantified into 33 chromatographic peaks. A high degree of variability was observed, with a median ratio of minimum to maximum values of 6.17 and significant age- and sex-specific differences. Heritability estimates varied widely for individual glycans, ranging from very low to very high. Glycan-wide environmental determinants with statistically significant effects of various variables such as diet, smoking, and cholesterol levels were also noted.

In 2011, Pučić et al., published the first large-scale population study of the IgG *N*-glycome applying a novel HT method for isolation of IgG from 2298 plasma samples.^69^ After IgG isolation, PNGase F was used to release the *N*-glycans attached to IgG followed by 2-AB labeling as described by Knežević et al.^27^ The results showed that nearly 96% of all neutral IgG glycans were core-fucosylated. Compared to other major plasma glycoproteins, IgG *N*-glycans are also less sialylated, with approximately 3% disialylated (A2G2S2) glycan structures. Agalactosylated and monogalactosylated structures were found to be 40% each, and digalactosylated structures represented 20% of the neutral IgG glycome. On average, 18% of neutral glycans contained a bisecting *N*-acetylglucosamine.

A study in Western Australia has shown that, in addition to the replicated association of BMI to agalactosylated IgG, measures of central adiposity explain the most variation in the IgG glycome and are associated with an increased abundance of pro-inflammatory IgG *N*-glycans (637 community-based individuals in the age range 45–69 years). This study suggests that the waist-to-height ratio or android/gynoid ratio should be considered instead of BMI when controlling for adiposity in IgG glycome biomarker studies.^74^

In the study by Greto et al., in 2021, an altered IgG *N*-glycome was observed after extensive weight loss following a low-calorie diet, bariatric surgery, or a decrease of BMI with time.^294^ The levels of bisecting *N*-acetylglucosamine substantially decreased after the low-calorie diet intervention following bariatric surgery, the core-fucosylated and agalactosylated glycans decreased, and an increase was observed for digalactosylated and monosialylated glycans, regardless of the type of surgery. The abundance of digalactosylated glycans also increased with a decrease in BMI, while the abundance of agalactosylated and high mannose glycans decreased with weight loss. These changes in relation to BMI drop for plasma-derived IgG *N*-glycan traits were observed by longitudinal monitoring of 1680 participants, and statistical analysis was performed on a subset of 3742 samples (measurements) that contained BMI information. These findings highlighted the effect of weight loss on IgG *N*-glycosylation. The largest population study, hitherto, holds the analysis of the IgG glycome over 10 000 individuals from 27 different populations.^29^ This study by Štambuk et al., confirmed strong associations between different IgG glycans (and Fc glycopeptides) and age but also the place of residence of an individual.^29^ Interestingly, several IgG glycans strongly correlated with the expected lifespan, suggesting that IgG glycome composition may correlate with all-cause mortality risk. Cardiovascular diseases (CVD) are the main cause of mortality, and Kifer et al., recently identified four IgG *N*-glycan traits that associate with an increased risk of incident hypertension.^295^ Three glycan traits, representing simple glycan structures containing a core fucose with only one or no galactose residues attached to the bisecting *N*-acetylglucosamine, significantly increase in individuals who developed hypertension during follow-up when compared with those defined as controls, whereas only one glycan structure decreases, a more complex digalactosylated structure with two sialic acid residues attached to the galactose molecules and without a bisecting *N*-acetylglucosamine. These results indicate that the IgG glycoprofile of individuals who would develop incidental hypertension during follow-up manifests a proinflammatory pattern and is associated with obesity years before diagnosis, which indeed supports a role of IgG glycosylation in the incident hypertension.

### 6.2 HT-Glycomics in Genetic Studies

The biosynthetic pathway of *N*-glycosylation is well defined, but very little is known about the genetic regulation of glycosylation. In 2010, Lauc et al. published the first GWAS of the *N*-glycome, which shed initial light on the associations between common genetic polymorphisms and protein *N*-linked glycosylation.^118^ The analysis of 2705 individuals from three population cohorts, from Croatia and Scotland, revealed that common variants in hepatocyte nuclear factor 1α (*HNF1A*) and fucosyltransferase genes *FUT6* and *FUT8* affect the *N*-glycan levels in human plasma. Subsequent functional studies confirmed that HNF1A regulates the expression of key fucosyltransferase and fucose biosynthesis genes and in this way acts as the main regulator of plasma protein fucosylation.^118^

As a sequel to the study of Lauc et al., the analysis was extended to 33 direct traits and 13 derived glycosylation traits in 3533 individuals by Huffman et al., using the same sample preparation protocol from 2011.^119^ In this European cohort, the previous findings were replicated and, additionally, three novel gene associations were identified (*MGAT5*, *B3GAT1*, and *SLC9A9*). While *MGAT5* encodes a glycosyltransferase which is known to synthesize the associated glycans, neither *B3GAT1* nor *SLC9A9* had previously been functionally associated with glycosylation of plasma proteins. Because B3GAT1 has glucuronyltransferase activity, Huffman et al. have shown that glucuronic acid is present on the antennae of plasma glycoproteins and detected the corresponding HPLC peak. *SLC9A9* encodes a proton pump that affects pH in the endosomal compartment. Recently, it has been reported that changes in the pH of the Golgi can affect protein sialylation,^326^ providing a possible mechanism for the observed association.

Sharapov et al., reported a GWAS of the composition of human plasma *N*-glycome in up to 3811 participants, which was measured by UHPLC.^314^ Starting with the 36 directly measured traits, an additional 77 derived traits were calculated, leading to a total of 113 glycan traits. From this, 12 different loci were discovered and replicated, the majority of loci contain genes encoding enzymes directly involved in glycosylation (*FUT3*/*FUT6*, *FUT8*, *B3GAT1*, *ST6GAL1*, *B4GALT1*, *ST3GAL4*, *MGAT3*, and *MGAT5*) and a known regulator of plasma protein fucosylation (*HNF1A*). Moreover, functional genomic annotation suggests a role for several other genes including *DERL3*, *CHCHD10*, *TMEM121*, *IGH*, and *IKZF1*. With an aim to verify the previous findings, a replication study was published using an independent set of 4802 individuals in 2021, where 36 directly measured traits and 81 derived glycan traits, resulting in a total of 117 glycan traits, were analyzed.^316^ Additionally, the association of three loci near the genes *PRRC2A* (*HLA* region), *RUNX3*/*MAN1C1*, and *SLC9A9*, which have not been identified before, was reported.

The first GWAS of an individual protein was performed on IgG, which is the most abundant plasma glycoprotein.^71^ This study implemented the IgG isolation procedure using monolithic protein G plates followed by the *N*-glycan release and labeling protocol as described by Pučić et al.^69^ HILIC-UHPLC was used as measurement and detection platform. Lauc et al. performed the first GWAS in 2013 to identify genetic loci associated with IgG *N*-glycosylation on 2247 individuals from the same European populations^71^ used for the GWAS study by Lauc et al. in 2010.^118^ Overall, nine genome-wide significant loci were identified of which four loci (*ST6GAL1*, *B4GALT1*, *FUT8*, and *MGAT3*) contained genes encoding glycosyltransferases. The remaining five loci (*IKZF1*, *IL6ST-ANKRD55*, *ABCF2-SMARCD3*, *SUV420H1*, and *SMARCB1-DERL3*) included genes that were previously not implicated in protein glycosylation, although most of them have strongly been associated with autoimmune and inflammatory conditions and/or hematological cancers. This study demonstrated that GWAS can be used to identify novel loci that control the glycosylation of a single plasma protein.

In 2020, the second and – up to now – largest GWAS study of IgG *N*-glycosylation was published by Klarić et al.^268^ Here, 8090 samples were analyzed from individuals of European ancestry and suggested how associated loci form a functional network. This new study doubled the number of associated loci compared to the first IgG *N*-glycome GWAS study in 2013.^71^ In total, 27 loci showed genome-wide significant associations, with another six loci showing suggestive significance. Thirteen of the genome-wide significant loci were found to validate prior findings, while 14 loci had never been linked to IgG glycosylation before. The same single nucleotide polymorphism (SNP) and glycan association was repeated in a meta-analysis of four different European cohorts (*N* = 2388) for 19 of 27 relevant loci (9 of 14 previously unassociated). There is no known role in glycosylation for any of the genes in the novelly associated loci.

Generally, GWAS studies indicated that regulatory networks that govern protein glycosylation include many other genes in addition to enzymes that are directly involved. These networks seem to be protein and/or cell-type specific, as indicated by the recent comparison of genes that regulate IgG and Tf glycosylation.^325^

### 6.3 HT-Glycomics in Clinical Studies

Glycome composition integrates genetic, epigenetic, and environmental factors, which makes glycans promising biomarkers for complex diseases. Furthermore, the glycocalyx, a thick layer of glycans on the surface of cells, engages in the contact of a cell and its environment and different cells with one another, including immune cells screening for danger.^327^ IgG glycans are involved in multiple humoral immune processes, such as ADCC, complement activation and complement-dependent cytotoxicity (CDC), antigen neutralization, target opsonization for phagocytosis, and hypersensitivity reactions. Significant alterations in the IgG glycome have also been reported in different diseases,^273,274,328−332^ making it a prospective biomarker for various diseases.

Rheumatoid arthritis (RA) is one of the most prevalent chronic inflammatory diseases which primarily involves the joints. Changes in IgG have been associated with RA, and most patients develop autoantibodies against IgG. Already in 1976, Mullinax et al. reported an apparent decrease in the galactose content of the Fc region of serum IgG from RA patients,^37^ which sparked the hypothesis that glycosylation of IgG might change in RA and could be part of the disease pathogenesis. On the basis of their work, Parekh et al. demonstrated in 1985 that the portion of IgG glycoforms that do not carry galactoses is particularly high for RA and primary osteoarthritis (OA) patients.^10^ This study was done on 42 IgG samples obtained from the serum of healthy controls and RA and OA patients.

Gindzienska-Sieskiewicz et al. analyzed IgG glycosylation from 50 patients with RA in comparison with 30 healthy controls and showed that galactosylation of IgG in patients with RA correlates with severity and duration of illness.^328^ Also, in patients with a long duration of RA, a significant decrease of galactose ratio in comparison with patients who have had arthritis for less than five years was observed. Patients with severe RA had a reduced IgG galactose content compared to the group of patients in remission. In the same study, it was demonstrated that a decrease of galactosylation is positively correlated with disease activity in patients with active RA.^328^ Gudelj et al., studied IgG glycosylation in RA in two prospective cohorts by measuring IgG *N*-glycan traits in 179 subjects who developed RA within 10 years using HILIC-UHPLC-FLD.^329^ In contrast to other studies, no correlation was observed in the decrease of galactosylation and sialylation in RA cases, with the time between recruitment to the study and RA diagnosis. These results indicate that decreased galactosylation might be a pre-existing risk factor involved in the disease development.

On the other hand, a HT glycomic analysis showed that serum protein *N*-glycosylation was strongly modified during pregnancy, both in RA patients and healthy controls.^302^ Using MALDI-TOF-MS, changes in the serum *N*-glycome were observed during 285 pregnancies (samples were collected before conception (only for RA patients), at three time points during pregnancy and at three time points after delivery). Specifically, an increase in galactosylation of diantennary *N*-glycans, an increase in tri- and tetraantennary species as well as an increase of α2,3-linked sialylation and a decrease in *N*-glycans with bisecting *N*-acetylglucosamine were found.^302^ The change in RA disease activity was shown to be negatively associated with the galactosylation of diantennary *N*-glycans and positively with the sialylation of triantennary fucosylated glycans.

Systemic lupus erythematosus (SLE) is a chronic autoimmune disease that predominantly affects women and certain ethnic groups.^333^ SLE condition is influenced both by genes and by the environment, which results in the production of pathogenic autoantibodies, mainly of the IgG1 and IgG3 subclasses. Vučković et al., analyzed the composition of the IgG *N*-glycome in 261 SLE patients and 247 matched controls of Latin American Mestizo origin and in two independent replication cohorts of different ethnicity (108 SLE patients and 193 controls from Trinidad as well as 106 SLE patients and 105 controls from China) using the HILIC-UHPLC-FLD.^273^ A significant difference was observed in the IgG *N*-glycome composition between patients and controls. The most significant changes included a decrease in IgG galactosylation, sialylation, core-fucosylation, as well as an increase in bisecting *N*-acetylglucosamine. The magnitude of observed changes was associated with the intensity of the disease, indicating that aberrant IgG glycosylation may be important for the molecular mechanism in SLE.

Inflammatory bowel disease (IBD) is considered to be a result of environment, gut microbiota composition, and aberrant immune response in genetically susceptible individuals. Trbojević-Akmačić et al., showed significant differences in the IgG *N*-glycome composition between patients with ulcerative colitis (UC) or Crohn’s disease (CD) compared with controls.^274^ The IgG *N*-glycome was analyzed in 507 patients with UC, 287 patients with CD, and 320 controls by HILIC-UHPLC-FLD. IgG galactosylation was significantly decreased in both UC and CD patients, while IgG sialylation was significantly decreased in CD.^274^ Moreover, larger HT-studies on the level of IgG Fc glycopeptides^289^ and total plasma *N*-glycome^312^ showed that changes in *N*-glycosylation associated with the disease and clinical features of IBD, indicating that IgG and total plasma protein *N*-glycan profiles have translational potential as IBD biomarkers.^289^

As mentioned before, glycans play a major role in the immune system and represent one of the main defenses against various pathogens. The viruses use host-cell machinery to glycosylate their own proteins during replication. Viral envelope proteins from a variety of human pathogens including HIV-1, influenza virus, severe acute respiratory syndrome coronavirus (SARS-CoV)-1, and SARS-CoV-2, Zika virus, dengue virus, and Ebola virus have evolved to be extensively glycosylated. These viral host-cell-derived glycans facilitate various structural and functional roles, from immune evasion enabled by glycan shielding to the enhancement of immune cell infection. Giron et al., profiled circulating glycomes (plasma and bulk IgG) in 47 HIV-infected individuals.^334^ CGE-LIF and lectin microarray were used for glycomic analysis. They identified several total plasma protein *N*-glycome traits associated with a time-to-viral-rebound in two geographically distinct cohorts.^334^ IgG glycosylation was also recently studied in 167 patients with mild COVID-19 and 166 patients with severe COVID-19 using a HILIC-UHPLC-FLD.^335^ A significant decrease in IgG *N*-glycans containing bisecting *N*-acetylglucosamine was reported in severe, compared to mild, COVID-19 cases. Another study, conducted by Wang et al., showed a decreased level of total IgG fucosylation, sialylation, and galactosylation in severe COVID-19 cases compared to controls.^336^ Moreover, LC-MS analysis of IgG1 Fc glycopeptides in a longitudinal cohort of COVID-19 patients has demonstrated significant changes in anti-S IgG1 *N*-glycosylation near the disease onset compared to total plasma IgG1. These differences in *N*-glycosylation of anti-S IgG1 and total IgG1 were rapidly diminishing during the disease course.^216^

Recently, glycobiology has gained importance in cancer research given its role in understanding various cancer mechanisms and for diagnostic application.^337−341^ Also, advances in the field of proteomics and glycomics have helped glycobiologists decipher the link between glycan structures and disease progression.^342^ Glycosylation acts as a key regulatory mechanism, controlling several physiopathological processes and defects in glycosylation are associated with various diseases, including cancers.^277,342−344^ While glycoproteins are some of the most common clinically used biomarkers for diagnosis and monitoring of malignant progression, their glycosylation has not, yet, been taken into account.^339,345−351^ However, it has been widely shown that glycosylation changes can be associated with cancer, of which most are related to sialylation, fucosylation, mucin-type *O*-glycans, as well as *N*- and *O*-linked glycan branching.^338,352,353^

Sialylated carbohydrates have an important role in cellular recognition, cell adhesion, and cell signaling. An increase in α2,3- and α2,6-linked sialylation has been closely associated with cancer.^354^ A study by Cheeseman et al. revealed that elevated concentrations of sialic acids in plasma and serum correlated positively with the presence of CVD, diabetes, and the development of malignant tumors.^344^ Another study by Laider et al. found a significant correlation between increased α2,6-sialylation and adhesion abilities of metastatic melanoma cells.^355^ In line with these observations, Kremser et al., identified an altered *N*-glycome by MALDI-MS on integrins α3β1 and α5β3 and revealed that glycan modifications have been related with increased melanoma cell motility, impacting cell–ECM interactions.^356^ Fucosylation has also been associated with cancer.^357^

Overexpression of core-fucosylated glycans is an important feature of lung cancer and breast cancer,^358,359^ and it is also reflected in the serum activities of fucosyltransferases during the process of hepatocarcinogenesis.^360^ Noda et al., showed a highly significant increase in the levels of α1,6-fucosylated α-fetoprotein (AFP) in patients with hepatocellular carcinoma (HCC) in comparison to chronic liver diseases which was in part caused by up-regulation of the α1,6-fucosyltransferase.^361^ West et al., analyzed 138 HCC tissue samples by in situ *N*-glycan MALDI-MSI and showed that during malignant transformation levels of complex β1,6-branched *N*-linked glycans, as well as levels of fucosylation, are increased compared to adjacent untransformed tissue or tissue from patients with liver cirrhosis without cancer.^362^ Moreover, increased fucosylation was associated with reduced survival time. This analytical approach overrides the need for microdissection and tissue solubilization prior to analysis, making it an attractive approach for HT screening of tissue samples.

Another common feature of cancer is the overexpression of the GalNAc-type *O*-glycans, also called mucin-type *O*-glycans. Mucin-type *O*-glycans are mostly found in the transmembrane and on secreted glycoproteins. Also, during malignancy, the incomplete synthesis of *O*-glycans can occur and lead to abnormal expression of truncated *O*-glycans; disaccharide Thomsen–Friedenreich antigen (T antigen, Galβ1-3GalNAcα1-*O*-Ser/Thr), monosaccharide GalNAcα1-*O*-Ser/Thr (also known as Tn antigen), and their sialylated form (STn-antigen, Neu5Acα2-6GalNAcα1-*O*-Ser/Thr).^363,364^ The recent application of precise and stable glycan gene editing in mammalian cell lines combined with a HT MS approach has contributed to the characterization of the *O*-glycoproteome of cancer cells and identification of *O*-glycoproteins containing STn-antigen in sera of gastric cancer and healthy individuals.^363,365^ Two candidate biomarkers carrying the STn-antigen were identified, CD44 and GalNAc-T5, with CD44 validated as expressed in gastric cancer tissue. In addition, STn has been found on plasminogen in serum from patients with intestinal metaplasia and gastritis.^366^ Plasminogen *O*-glycans were analyzed by nano-HPLC-MALDI-TOF/TOF after lysine-sepharose affinity chromatography, in-gel de-*O*-glycosylation by reductive β-elimination and permethylation.^366^ A recent study that explored the serum protein *O*-glycosylation in 62 CRC patients and 20 healthy individuals by Gizaw et al.^367^ has revealed differences in *O*-glycan patterns between cancer samples and healthy controls that correlated with cancer progression. Moreover, *O*-glycan profiles differed between male and female patients during cancer progression.

Although all of the above-mentioned biomarkers have shown an aberrant glycosylation in relation to cancer,^368−370^ they have limited application due to their relatively low specificity and sensitivity. With the advent of new methods and technologies for glycan analysis, new biological information is generated, and potential disease biomarkers can be identified. Additionally, the newly developed HT platform technologies have enabled the analysis of large cohorts of samples in an efficient manner.^371^

For example, Miyoshi et al., observed an increased concentration of fucosylated haptoglobin in serum of patients with pancreatic cancer compared with healthy controls as well as with patients diagnosed with other types of cancer, e.g., HCC, gastric cancer, or CRC.^372^ Site-directed analysis of haptoglobin *N*-glycopeptides by RP-HPLC-ESI-MS showed a site-specific increase in fucosylation of diantennary glycans on two *N*-glycosylation sites and an increase in fucosylation of triantennary glycans on all four *N*-glycosylation sites in pancreatic cancer patients compared to chronic pancreatitis and healthy individuals. Further clinical investigation on 100 patients with CRC located near the liver showed higher levels of fucosylated haptoglobin,^372^ implicating cancer location as an important factor for changes in haptoglobin fucosylation. Moreover, haptoglobin glycosylation, along with Tf glycosylation, has shown to change in ovarian cancer,^121^ and along with IgG, Tf, and AGP glycosylation in patients bearing stomach adenocarcinoma.^373^

Glycosylation in CRC has also been studied on the level of total plasma *N*-glycome^313^ and IgG *N*-glycome.^277^ It was shown that CRC associated with a decrease in IgG galactosylation, IgG sialylation, and an increase in core-fucosylation of neutral *N*-glycans with a concurrent decrease of core-fucosylation of sialylated *N*-glycans. A model based on age, sex, and *N*-glycans showed a good discriminative power between CRC cases and controls (area under the curve = 0.755) implicating that variation in IgG *N*-glycosylation could be important for the prediction of disease course. Statistically significant differences were observed also on the level of the plasma *N*-glycome. A decrease in the core fucosylated diantennary glycans F(6)A2G2 and F(6)A2G2S(6)1 was highly significant at all stages of CRC. Additionally, stage 1 showed a unique biomarker signature compared to stages 2 up to 4.^313^

Altered total serum protein *N*-glycosylation (both fucosylation and sialylation) has been shown in a study on 13 sera with benign prostate hyperplasia (BPH) and 34 PCa samples, using HT normal phase (NP) and weak anion exchange (WAX)-HPLC *N*-glycan analysis in combination with exoglycosidase digestion.^374^ The levels of diantennary *N*-glycans containing core-fucose and *N*-glycans containing α2,3-linked sialic acids were significantly increased in PCa patients compared with patients with BPH. Triantennary trigalactosylated *N*-glycans and tetraantennary tetrasialylated *N*-glycans with antennary fucose were significantly decreased, and tetraantennary tetrasialylated *N*-glycans increased in Gleason score 7 compared with Gleason score 5 PCa patients. Identification of glycosylation patterns specific for BPH, Gleason 7, and Gleason 5 PCa patients with the used technology, implicated its potential use for noninvasive diagnosis. PCa, BPH patients, and healthy controls were also shown to have different total urine *N*-glycosylation profile (after desialylation) analyzed using a DNA sequencer-aided fluorophore-assisted carbohydrate electrophoresis (DSA-FACE) system. The urine *N*-glycome (after digital rectal examination) and prostate volume were combined into a urinary glycoprofile marker (UGM) that was able to discriminate between PCa and BPH.^375,376^ PSA has been recognized as a specific biomarker for PCa that can distinguish it from BPH. Measurement of a2,3-linked sialylation on PSA, which is characteristic for PCa patients, may improve the accuracy of early detection.^377^

**Figure 15:**
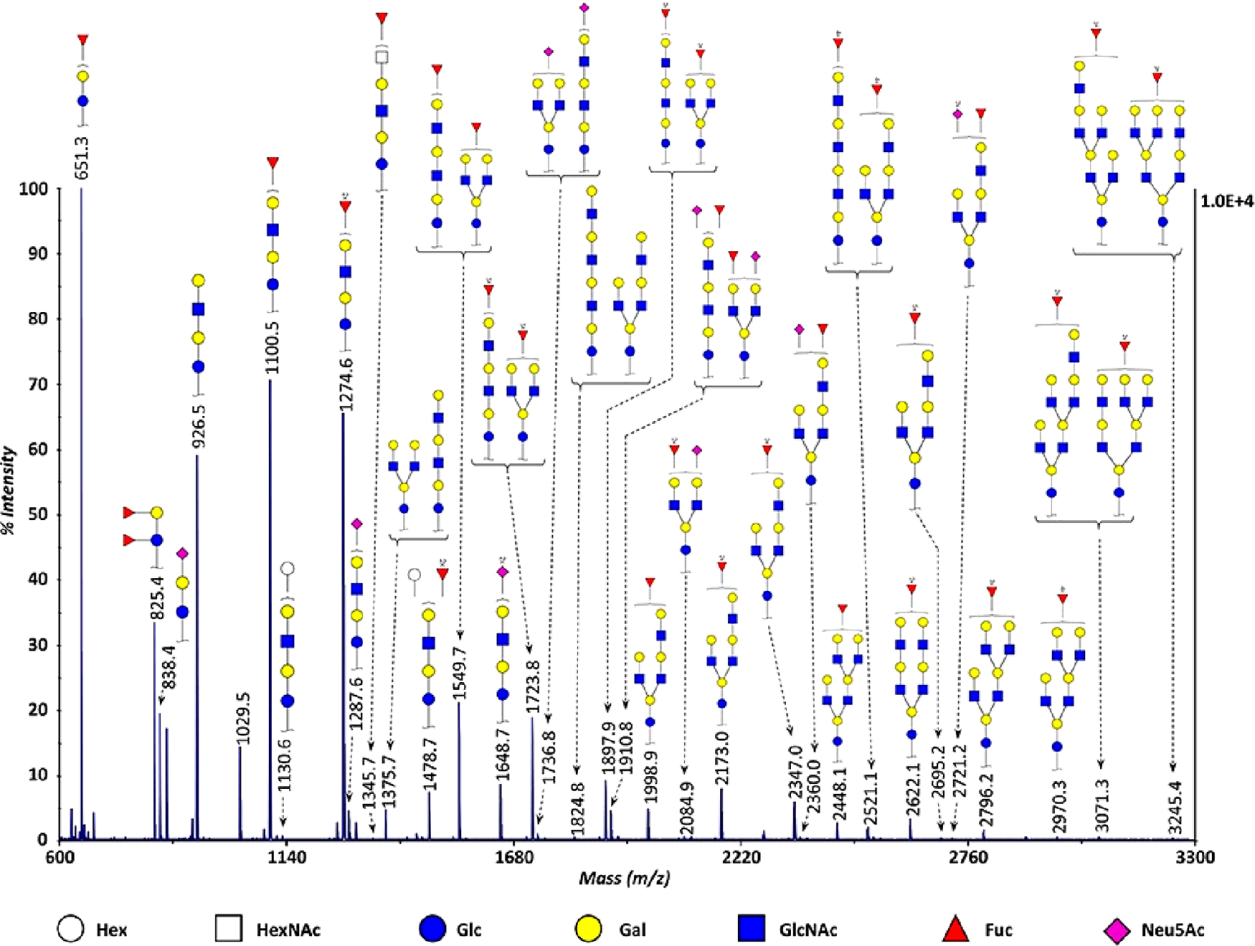
Representative MALDI-TOF-MS spectrum of permethylated HMOs. All masses correspond to fully permethylated, free reducing end, sodium adducts of HMOs. Possible structures for each mass are shown. Free lactose was excluded from the spectrum. HMOs were identified using GlycoMod. Reproduced with permission from ref (16). Copyright 2020 Oxford University Press.

HMOs are another clinically relevant glycan class with extensive nutritional and health benefits for infants.^378,379^ Because of their extreme complexity and diversity their comprehensive analysis, especially in HT mode, remains challenging. Significant progress in the quantification of this diverse glycan class has been made in the past decade^16,17,380,381^ (Figure 15), facilitating analysis in large-scale studies. Current knowledge on associations of HMO content with infant growth and health status is extensively reviewed by Sprenger et al.^379^ Most notably, a study done by HPLC-FLD analysis of 2-AB labeled oligosaccharides from 410 human milk samples showed that HMO concentrations and profiles differ between healthy women from different geographical regions.^382^ Analysis of HMOs by nano-LC-chip TOF-MS^15^ in two cohorts of 303 Malawian mother–infant sets showed a lower abundance of fucosylated and sialylated HMOs in breast milk of nonsecretor mothers (negative for the functional enzyme encoded by the fucosyltransferase 2 gene), whose infants were minimal compared to those showing normal growth.^381^ Another recent study employing the same analytical technology on 659 human milk samples collected at six months postpartum aimed to explore the association of HMOs and bioactive proteins with markers of inflammation and longitudinal prevalence of infant morbidity.^323^ Differences in the relative abundance of HMOs were observed between secretor and nonsecretor mothers, mainly a lower abundance of fucosylated HMOs and a higher abundance of sialylated HMOs in nonsecretors. Additionally, depending on the secretor status, significant associations of HMOs and bioactive proteins with inflammatory markers and infant morbidity were observed.^323^ Considering longitudinal changes in HMO composition during lactation^383^ and the heterogeneity of HMOs in women of, e.g., different geographical backgrounds or secretor status, more extensive longitudinal studies focused on different populations are needed.

Relevance of HMOs in the context of infant growth,^381,384^ allergies,^385,386^ and other diseases^379^ makes it an important glycan class also in the context of formula-fed infants’ health, as well as in the biopharmaceutical industry.^387^ Today, HMOs are synthesized artificially and added to infant milk formulations and baby food. As such, their analysis is subjected to stringent controls warranting highly robust and sensitive HT methods.

### 6.4 Glycoprofiling of Biopharmaceuticals

Two-thirds of therapeutic proteins currently on the market are glycoproteins. Glycans have been recognized as a critical quality attribute influencing function, efficacy, and safety of a drug, making their analysis essential throughout the screening of new drug candidates, product development, and manufacturing processes as well as in comparing biosimilars and biobetters to originator drug.^388^ Biotherapeutic glycoproteins are predominantly mAbs based on IgG1, Fc-fusion proteins, and cytokines.

The potency of therapeutic mAbs depends on effective antigen recognition combined with an apt effector function, which is largely dependent on the structure of Fc *N*-glycans. Core-fucose is known to decrease IgG binding to FcγRIIIA receptor reducing the ADCC,^389,390^ while terminal galactose increases the CDC, sialic acid affects inflammatory properties, and mannose affects pharmacokinetics.^388^ Glycosylation itself, if containing nonhuman glycans originating from, e.g., murine cell lines, can be immunogenic which compromises drug safety. Moreover, different host cellular production systems result in distinct glycosylation profiles while cell culture conditions, e.g., nutrients, oxygen level, and pH, also influence glycosylation,^391^ causing structural and functional changes and affecting therapeutic behavior. Upstream processing conditions and their effect on glycosylation must be monitored to ensure the final drug quality and safety as well as batch-to-batch consistency. Glycoengineering through culture conditions manipulations, gene knockouts, and expression of glycan-modifying enzymes is one of the approaches used in biotherapeutic glycoprotein development that also requires glycan characterization throughout the process.

**Figure 16:**
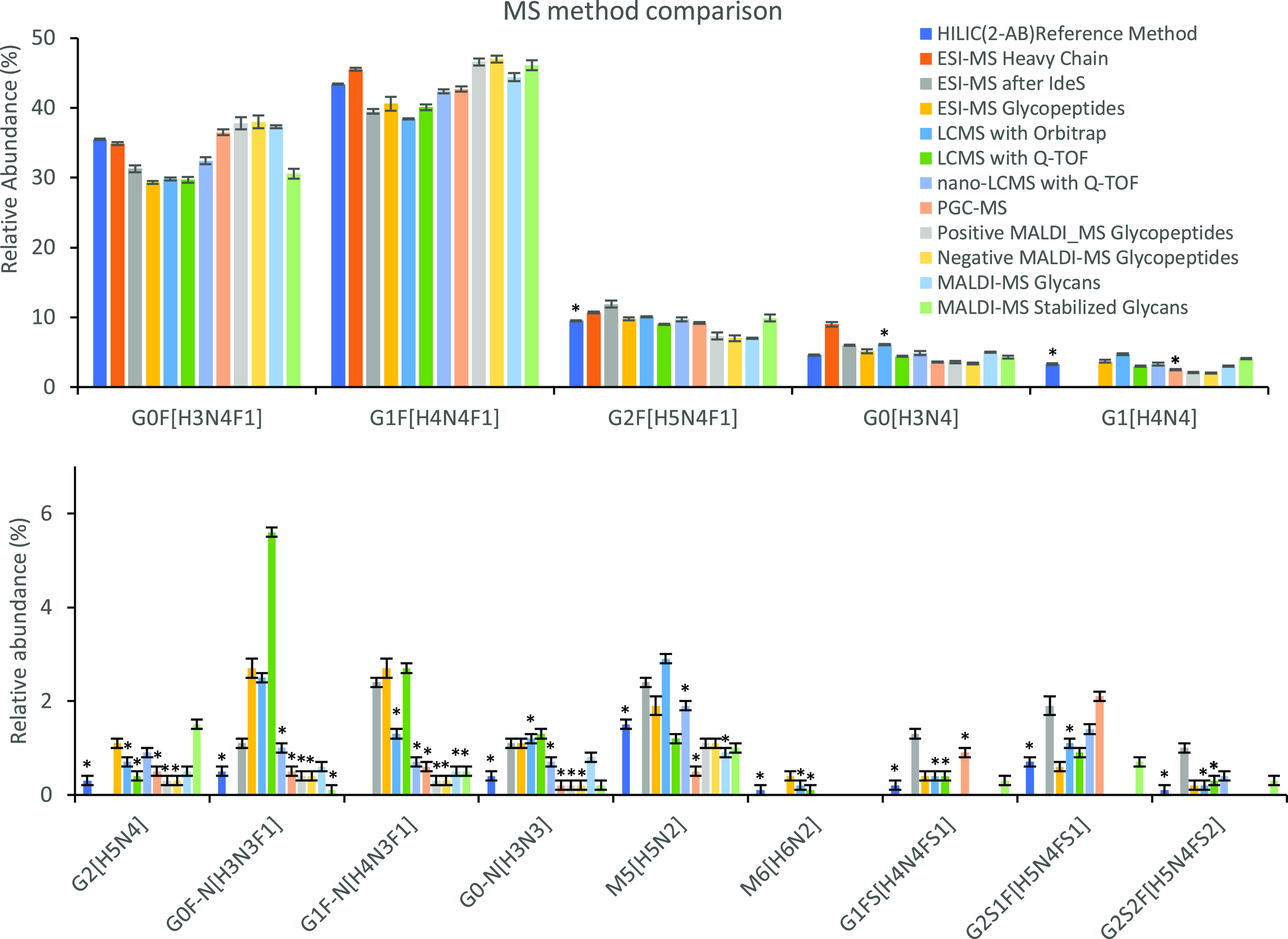
Relative quantitative evaluation of MS-based method performance in the analysis of therapeutic IgG Fc glycosylation. Each analytical method was applied in a batch of six replicates (the first set taken from the data set). Error bars represent the standard deviation. G1[H4N4] was not quantified for the ESI-MS after IdeS method. M6[H6N2], G1FS[H4N4FS1], and G2S1F [H5N4FF1S1] were not quantified for the analysis of glycopeptides in positive and negative ionization mode using MALDI-MS. All other missing bar graphs indicate those species were not detected with that specific method. Key: H, hexose; N, *N*-acetylhexosamine; F, deoxyhexose; S, *N*-acetylneuraminic acid (sialic acid); G0F-N, agalactosylated, core-fucosylated, monoantennary species. Data was obtained from ref (394) Copyright 2015 Reusch, et al.

Recent interlaboratory study on the glycosylation analysis of a NIST mAb reference material has demonstrated the applicability of a wide range of analytical methods for glycan analysis on the level of free glycans, glycopeptides, protein fragments, and intact mAb.^392^ However, not all of these methods have a high level of robustness necessary for HT applications and/or are not easily applicable in biopharmaceutical companies due to more stringent requirements resulting in the use of well-established and conservative methods, e.g., LC-FLD or LC-MS analysis of fluorophore-labeled glycans (Figure 16). Classical labeling approaches used in the biopharmaceutical industry (e.g., 2-AB and 2-AA labeling) are slowly being replaced by rapid deglycosylation and labeling of therapeutic glycoproteins using glycan analysis kits containing Rapid PNGase F and RapiFluor-MS^240^ or InstantPC labels.^129,393^ These novel labels provide increased sensitivity and higher ionization efficiency compared to traditional 2-AB and 2-AA, allowing for parallel analysis of the same sample with orthogonal techniques, e.g., LC-FLD and LC-MS, without the additional sample preparation steps. Moreover, increased sensitivity allows the detection of low abundant glycoforms. In line with the rapid labeling chemistries also goes robotization of sample preparation for less hands-on time, allowing the sample preparation of samples in a 96-well format in a few hours.^240^ Derived glycosylation traits are often used for the comparison of different biotherapeutics because they relate to differences in circulation half-time or mAbs effector functions.

Lectin microarrays have been shown to have the potential as a rapid orthogonal technique in glycosylation screening during therapeutic glycoprotein development.^235,237^ With relatively high throughput and sensitivity, lectin microarrays are especially advantageous during process development. However, because of the semiquantitative nature of results, for structural confirmation and more accurate quantitation, the use of standard methods such as U(H)PLC-FLD, CGE-LIF, and MS is warranted.

Lectin–glycan recognition has recently been coupled with the biolayer interferometry into a rapid HT screening platform based on biotinylated recombinant prokaryotic lectins and streptavidin-coated sensor.^395^

Some of the most widely used biotherapeutics, e.g., Fc-fusion protein etanercept, are also *O*-glycosylated, with glycosylation potentially affecting their biological function. Therefore, complete characterization of therapeutic glycoproteins’ glycosylation is of utmost importance. Reproducible and sensitive *O*-glycosylation profiling entails specific analytical challenges as already described above. Usually, sequential analysis of both *N*- and *O*-glycans is performed on the same sample, relying mostly on in-depth LC-MS profiling techniques. Comprehensive analysis of site-specific *N*- and *O*-glycosylation of etanercept by HILIC-UHPLC-FLR and LC-MS has been described by Houel et al.,^396^ while in-depth site-specific *O*-glycosylation profiling of glucagon-like peptide-1 (GLP1)-Fc fusion protein containing linker peptide has been recently described by Hashii et al.^397^*O*-Glycosylation analysis of therapeutic glycoproteins would benefit from future improvements of current strategies for *O*-glycan analysis and development of new ones.

### 6.5 Potential Diagnostic Applications

#### 6.5.1 Liver Fibrosis

Liver fibrosis is a result of chronic liver damage accompanied by the accumulation of extracellular matrix proteins, which is a characteristic of most types of chronic liver diseases. The main causes of liver fibrosis are alcohol abuse, chronic hepatitis C virus (HCV) infection, and nonalcoholic steatohepatitis.^398^ If advanced, liver fibrosis leads to cirrhosis, liver failure, and portal hypertension, often requiring liver transplantation. Diagnosis of liver fibrosis in the early stages of the disease remains challenging due to the absence of symptoms until the disease already progressed, warranting the development of noninvasive early stage biomarkers.

In 2001, Callewaert et al., used a DSA-FACE to profile sialidase-treated *N*-glycans from the whole serum,^59^ which was the basis for the development of a cirrhosis biomarker (GlycoCirrhoTest)^60^ and a fibrosis biomarker (GlycoFibroTest)^399^ for patients with chronic HCV infection. Cao et al., adapted the DSA-FACE approach on a CE-based ABI 3500 system and simplified the procedure to four main steps: *N*-glycan release, *N*-glycan labeling, removal of sialic acids, and *N*-glycan profiling.^400^ The sera of 432 hepatitis B virus (HBV)-infected patients with liver fibrosis were analyzed to investigate the correlation between *N*-glycans and HBV-induced liver fibrosis and to verify a multiparameter diagnostic model related to changes in the serum *N*-glycome.^400^ Significantly changed *N*-glycan peaks in different fibrosis stages were selected in the modeling group, and multiparametric diagnostic models were established based on changed *N*-glycan levels by logistic regression analysis. *N*-Glycans models were compared with the aspartate aminotransferase to platelet ratio index (APRI), fibrosis index based on the four factors (FIB-4), glutamyltranspeptidase platelet albumin index (S index), GlycoCirrhoTest, and GlycoFibroTest. They found that the alterations of serum *N*-glycans are associated with HBV-related liver fibrosis and showed that multiparameter *N*-glycan models are powerful in diagnosing early stage fibrosis.

#### 6.5.2 Diabetes

Diabetes mellitus is a high-prevalence heterogenic group of metabolic disorders characterized by the presence of hyperglycemia. This is a lifetime disease leading to reduced life expectancy, premature morbidity, and mortality. Because the disease burden is rising worldwide, many studies aim to identify predisposing factors, establish an early diagnosis, and correct classification of the disease as well as identify novel therapeutic targets.

In 2018, Juszczak et al. published research on 989 individuals diagnosed with diabetes when younger than 45 years of age (so-called “maturity-onset diabetes of the young” or MODY).^78^ MODY is caused due to variants in the *HNF1A* gene, and it is frequently misdiagnosed. Considering previous findings that plasma levels of antennary fucosylated *N*-glycans and high-sensitivity C-reactive protein (hs-CRP) are reduced in individuals with HNF1A-MODY, a nongenetic biomarker could improve subject selection for genetic testing and increase diagnostic rates. This study suggested the potential use of *N*-glycans and hs-CRP in discriminating individuals with damaging *HNF1A* alleles from those without HNF1A variants by identifying 29 individuals harboring 25 rare *HNF1A* alleles, of which 3 were novel and 12 were considered pathogenic. Antennary fucosylated *N*-glycans and hs-CRP were able to differentiate subjects with damaging *HNF1A* alleles from those without rare *HNF1A* alleles. *N*-Glycan release, labeling, and cleanup were performed as described previously by Trbojević-Akmačić et al. in 2015.^73^ Fluorescently labeled glycans were separated by HILIC-UHPLC-FLD into 42 chromatographic peaks, which enabled reliable quantification.

#### 6.5.3 Breast Cancer

Breast cancer is currently the most prevalent cancer worldwide and the most dominant cause of cancer-related deaths in women.^401^ Early detection and diagnosis significantly improve the survival rate. However, currently used diagnostic markers are not fairly specific and sensitive, calling for more reliable biomarker identification. Glycosylation changes of serum proteins and tumor tissue have been associated with breast cancer progression having potential as new prominent diagnostic biomarkers.^402,403^

In 2016, Ju et al. reported five difucosylated *N*-glycans as potential biomarkers in initial breast cancer and recurrent breast cancer patients.^404^ They analyzed free permethylated *N*-glycans from serum glycoproteins in 134 samples, including 91 breast cancer sera and 43 healthy controls using linear ion-trap quadrupole (LTQ)-ESI-MS and observed significant changes in *N*-glycosylation between healthy controls, initial diagnostic patients, and recurrent patients. By comparing breast cancer patients and controls, a positive correlation was found between increased difucosylation and disease progression. Also, increased difucosylated *N*-glycans, containing both core and antennary fucose, could be a more reliable indicator of recurrent breast cancer because it was proved to outperform currently used breast cancer biomarkers, such as carcinoembryonic antigen (CEA), CA 15–3, and CA 125.

Recently, Terkelsen et al. have reported *N*-glycan signatures from tumor interstitial fluid (TIF, *n* = 85), paired normal interstitial fluids (NIF, *n* = 54), and serum of breast cancer patients (*n* = 28) as well as their association with clinical outcomes.^405^*N*-Glycans from the samples were released using a HT automated method by a liquid-handling robot (2-AB labeling) and analyzed by HILIC-UHPLC-FLR. An increase in the expression levels of nine *N*-glycans were reported, containing bisecting *N*-acetylglucosamine contributing to tumor suppression in NIF. Furthermore, levels of five *N*-glycans in TIF correlated with that in paired serum. To sum up, these results imply that profiling of *N*-glycans from breast tumor fluids is a promising biomarker for detection of tumor-derived glycan-signatures in the blood and may improve the diagnostic and prognostic stratification of patients with breast cancer.

Another recent age-matched case-control study explored the biomarker potential of total serum *N*-glycome for the detection of breast cancer. Analysis of total serum protein *N*-glycome by MALDI-FTICR-MS from 145 breast cancer patients and 171 healthy individuals showed that serum *N*-glycome differs between various cancer subtypes. Of note, some global total serum protein *N*-glycomic signatures that had previously been reported between breast cancer patients and controls were not replicated in the current cohort, implicating the effect of the heterogeneous character of the disease on *N*-glycome profiles.^406^

Heavily *O*-glycosylated mucin 1 is a recognized biomarker for the diagnosis of breast cancer.^407,408^ It has been known that the alterations of mucin *O*-glycans occur in the mammary gland during malignancy,^408^ and developing comprehensive *O*-glycan profiling workflows is an alternative strategy for the breast cancer biomarker discovery. Kirmiz et al., used the MALDI-FTICR-MS-based approach to analyze *O*-glycans extracted from breast cancer cell line supernatant, serum of a breast cancer mouse model, and human breast cancer patient samples.^409^ Observed differences in *O*-glycan profiles were sufficient to distinguish the patients with breast cancer from those without cancer by PCA, although the findings were tentative due to the small number of samples involved in the study. Also, similar glycan patterns were observed as in serum from ovarian cancer patients.^410^

#### 6.5.4 Intact Analysis of Transferrin

Human Tf is a well-known biomarker of congenital disorders of glycosylation (CDGs),^411−413^ and the diagnosis is mainly based on the glycoform pattern observed for intact Tf by isoelectric focusing (IEF).^414^ However, using this method, it is difficult to discriminate between CDG-I and CDG-II and it often leads to false-negative results.^415^ Therefore, for fast and high sensitivity profiling and accurate characterization of heterogeneous glycan structures, high-performance separation techniques coupled to MS are used.^416,417^ Tegtmeyer et al., reported that using MS to analyze intact Tf in some patients results in a combination of Tf isoforms with a lack of glycans and abnormal glycan structures lacking galactose.^418^ Abu et al. analyzed plasma Tf from 19 CDG samples by high-resolution qTOF-MS, covering a broad range of biochemical and clinical severity.^419^ Also, to define normal Tf glycosylation, they analyzed 20 healthy volunteers by Tf-IEF and qTOF-MS. Total plasma glycoprofiling showed a decrease in galactosylation and sialylation in most phosphoglucomutase-1 (PGM1)-CDG patients, while fucosylation and high mannose glycans were increased. Also, using the qTOF-MS Tf glycoprofiling approach, Tf was revealed to be a highly sensitive and specific glycomarker for the diagnosis, as well as the primary test for PGM1-CDG.

More recently, Hipgrave et al., studied glycan profiles for Tf and IgG Fc from CDG patients and healthy controls using ESI-MS of intact proteins and (LC)-MS of tryptic glycopeptides.^420^ They reported lower levels of sialylated structures on plasma proteins as compared to healthy controls due to defects in proteins involved in Golgi trafficking and cytidine monophosphate (CMP)-sialic acid transport. Also, using MALDI-TOF-MS, they differentiated sialic acid linkage isomers via derivatization and reported unprecedented sialic linkage-specific effects, specifically, α2,3-sialylation might feature as a potential marker in future investigations.

## 7 Future Perspectives

Hitherto, HT glycomics is focused on *N*-glycan analysis and largely ignores other types of glycosylation. This is mostly due to a lack of universal enzymes that enable easy and HT workflows, as is the case for HT *N*-glycomics analyses, which are often accomplished at the PNGase F released *N*-glycan level. In the coming years, these relatively broadly disseminated HT *N*-glycomics analyses will expectedly benefit from the implementation of standards, both for identification and quantification purposes. Chemoenzymatic synthesis of *N*-glycans is rapidly evolving, including the development of stable isotope-labeled glycan standards.^421−423^ Implementation of glycan standards at various levels of the samples preparation and measurement workflows will not only allow highly accurate quantification but will also vastly improve the robustness of glycomic workflows, paving the way for the dissemination of glycoanalyses into nonglyco-specialist and routine laboratories. MS glycomic assays will particularly benefit from the broad use of standards, which will accelerate the implementation of glycomic assays in clinical diagnostics applications as well as the characterization of glycoprotein drugs in the biopharmaceutical industry.

Next to HT *N*-glycomics, the analysis of HMOs increasingly receives attention, and large numbers of samples are being analyzed at increasing depth, using a diverse range of methodologies, including HILIC-UHPLC-FLD, CE-LIF, and MS.^383,424−427^ HMO analysis contributes to deciphering the role and repertoire of bioactive carbohydrates and glycan-based neutraceuticals with potential health benefits, adding to our increasing insights into the link between metabolic disorders, glycome, and immune status.^319,428^

Regarding non-MS-based HT glycomics applications, relatively cheap, long-established labels such as 2-AB and APTS are still dominating. However, new labels with advantageous chromatographic and fluorescence, as well as MS detection, properties will increasingly be implemented. Also, the development of RP-UHPLC applications would allow HT-glycomics applications to be implemented on common nano-LC-high resolution-MS/MS platforms, which are broadly available in MS proteomics laboratories and will allow high-sensitivity detection of labeled glycans in HT mode. Expectedly, RP applications will gain ground relative to the currently dominating HILIC applications.

MS analysis of glycopeptides is rapidly advancing in HT glycomics applications, as it often allows protein- and site-specific glycosylation profiling. Similar to what was mentioned above, stable isotope-labeled glycopeptide standards will be very valuable for the smooth, further development and dissemination of these assays. Currently, HT glycopeptide analysis assays cover typically only a few proteins and not more than a dozen different peptide portions, with several hundred glycopeptide species quantified in an LC-MS run. With MS allowing multiplexed detection of complex glycopeptide mixtures featuring both profound peptide and glycan heterogeneity, the coverage of hundreds and potentially thousands of *N*-glycosylation sites should be feasible regarding analyte detection by LC-MS, and such analyses have repeatedly been performed in low-throughput mode. For HT glycopeptide-based analyses of complex mixtures, at the above-mentioned depth, robust HT sample preparation workflows will be needed, together with powerful tools for glycopeptide assignment and quantification from the complex LC-MS data, as well as suitable postprocessing data analysis strategies.

Intact glycoprotein analysis is another, very promising mode for HT protein glycosylation analysis, and while this approach has historically not been pursued a lot, it has vast potential. Importantly, in the biopharmaceutical industry, intact protein analysis is increasingly being implemented as a drug release assay, highlighting the power of these approaches. Similarly, intact glycoprotein analysis has already found its way into clinical diagnostics, showcasing its suitability for various types of applications. Strikingly, sample preparation workflows are often relatively simple, with a single affinity purification step sufficing, and increased implementation of such assays for HT glycoproteomics applications in clinical diagnostics and biopharma is foreseen.

Of note, both glycopeptide and intact glycoprotein-based HT glycomics workflows are amenable for the characterization of both *N*- and *O*-glycosylated proteins as demonstrated for human polyclonal IgG and IgA^19^ and for apoCIIIa analysis.^322^ While innovation on *O*-glycan release methods is continuing, the glycopeptide- and intact glycoprotein-based MS approaches will expectedly be the major drivers of HT *O*-glycomics in the coming years. Other fields, such as glycolipidomics and GAG analysis are still lagging behind concerning the development and implementation of HT glycomics applications.

MS-based HT glycosylation profiling methods can benefit from the implementation of ion mobility at the intact analyte level, i.e., right after analyte ionization.^201^ This extra dimension in glycan or glycopeptide analysis is beneficial in cases where isomer separation is achieved. Ion mobility is a promising addition for both MALDI and ESI ionization modes and is particularly paying off in combination with TOF analyzers.

Tissues and cell types differ wildly in their glycosylation repertoire, and only a very limited portion of this variety has hitherto been mapped. MSI has already for several years reached the level of spatial resolution that allows analysis of *N*-glycomic diversity in tissues at the cellular level.^429−435^ Approaches to assess *O*-glycans, glycolipids, and GAGs are still lagging behind, largely due to limitations in on-tissue sample preparation, with the lack of broad-specificity enzymes presenting a major bottleneck.^436^ Regarding on-tissue *N*-glycomics, recent improvements in ionization (MALDI-2) help to perform in-depth *N*-glycomic analysis with a decent dynamic range and spatial resolution.^201^ The implementation of specific derivatization techniques^176,437^ as well as ion mobility separation, provide big steps forward toward resolving glycan isomers, which is key for assessing the glycomic status of a tissue or cell type. Implementing these recent improvements in *N*-glycan MSI for the analysis of large tissue microarrays will bring HT *N*-glycomics to the next level and provide unprecedented insights into the glycobiology of tumor development as well as mucosal host–microbe and host–pathogen interactions.

For the years to come, glycomics and glycoproteomics analyses will come with making choices along the axes of throughput, analytical depth, and sensitivity. Future developments are sorely needed to further increase the speed, ease, and throughput of glycomics analyses for supporting dissemination into less specialized laboratories, including clinical diagnostics and various biopharma applications. At the same time, an increase in analytical depth is pursued, moving toward full structural definition of analytes with regard to glycan structure, glycosylation site, and contribution of glycosylation to the overall structural and functional proteoform repertoire. Additional depth is sought by covering thousands of glycosylation sites at the glycopeptide level,^438^ which is currently still at the cost of full structural elucidation of glycans as well as throughput. Highly promising are the high-sensitivity glycomics workflows, often relying on established proteomics and genomics hardware and platforms such as high-end nano-LC-MS/MS that can serve proteomics and glycomics purposes alike, as well as DNA analyzers for CE-LIF glycan analysis at utmost sensitivity and throughput. In fact, implementation of HT glycomics on HT platforms designed for other omics disciplines such as genomics and proteomics has proven a particularly successful approach, and further convergence and integration of HT omics approaches is envisioned.

## Abbreviations

%area: relative percentage area
%rPHP: relative peak height proportions
2-AA: 2-aminobenzoic acid
2-AB: 2-aminobenzamide
2-ANTS: 2-amino-1-naphthalenesulfonic acid
2-PA: 2-aminopyridine
2D: two-dimensional
3D: three-dimensional
ADCC: antibody-dependent cellular cytotoxicity
ADHD: attention-deficit hyperactivity disorder
AFP: α-fetoprotein
AGP: α-1-acid glycoprotein
APRI: aspartate aminotransferase to platelet ratio index
APTS: 8-amino-1,3,6-pyrenetrisulfonic acid trisodium salt
AQC: 6-aminoquinolyl-*N*-hydroxysuccinimidyl carbamate
ART: antiretroviral therapy
BMI: body mass index
BPH: benign prostate hyperplasia
CD: Crohn’s disease
CDC: complement-dependent cytotoxicity
CDG: congenital disorder of glycosylation
CE: capillary electrophoresis
CEA: carcinoembryonic antigen
CGE: capillary gel electrophoresis
CMP: cytidine monophosphate
COVID-19: coronavirus disease 2019
CRC: colorectal cancer
CVD: cardiovascular disease
CZE: capillary zone electrophoresis
DEN: dopant-enriched nitrogen
DHB: 2,5-dihydroxybenzoic acid
DMSO: dimethyl sulfoxide
DSA-FACE: DNA sequencer-aided fluorophore-assisted carbohydrate electrophoresis
ECM: extracellular matrix
EIC: extracted ion chromatogram
ELISA: enzyme-linked immunoassay
ESI: electrospray ionization
Fab: antigen-binding fragment
Fc: fragment crystallizable
FIB-4: fibrosis index based on the four factors
FLD: fluorescent detector
Fru: fructose
FTICR: Fourier transform ion cyclotron resonance
GAGs: glycosaminoglycans
Gal: galactose
GHP: hydrophilic polypropylene, GH Polypro
Glc: glucose
GLP-1: glucagon-like peptide-1
GSLs: glycosphingolipids
GU: glucose unit
GWAS: genome-wide association study
HBV: hepatitis B virus
HCA: hierarchical cluster analysis
HCC: hepatocellular carcinoma
HCV: hepatitis C virus
HILIC: hydrophilic-interaction liquid chromatography
HIV: human immunodeficiency virus
HMOs: human milk oligosaccharides
HNF1A: hepatocyte nuclear factor 1α
HPLC: high-performance liquid chromatography
hs-CRP: high-sensitivity C-reactive protein
HT: high-throughput
IBD: inflammatory bowel disease
IEF: isoelectric focusing
IgA: immunoglobulin A
IgG: immunoglobulin G
LC: liquid chromatography
LIF: laser-induced fluorescence
LTQ: linear ion-trap mass spectrometer
mAbs: monoclonal antibodies
MALDI: matrix assisted laser desorption/ionization
MIRAGE: Minimum Information Required for a Glycomics Experiment
MODY: maturity-onset diabetes of the young
MS: mass spectrometry
MSI: mass spectrometry imaging
MTU: migration time unit
MTU′′: double migration time alignment
NFIs: normalized fluorescent intensities
NicAc: nicotinic acid
NIF: normal interstitial fluids
NHS: *N*-hydroxysuccinimide
NMR: nuclear magnetic resonance
NP: normal phase
OA: osteoarthritis
PCA: principal component analysis
PCa: prostate cancer
PGC: porous graphitic carbon
PGM1: phosphoglucomutase-1
PNGase F: peptide-*N*-glycosidase F
ProA: procainamide
PSA: prostate-specific antigen
PVDF: polyvinylidene fluoride
RA: rheumatoid arthritis
RMS: root-means-squared
RP: reversed-phase
rt-PA: recombinant tissue-type plasminogen activator
S/N: signal-to-noise
S index: glutamyltranspeptidase platelet albumin index
SARS-CoV: severe acute respiratory syndrome coronavirus
SDS-PAGE: sodium dodecyl sulfate-polyacrylamide gel electrophoresis
SLE: systemic lupus erythematosus
SNP: single nucleotide polymorphism
SPE: solid-phase extraction
T1D: type 1 diabetes
T2D: type 2 diabetes
TAG: toolbox accelerating glycomics
Tf: transferrin
TIC: total ion current/count
TIF: tumor interstitial fluid
TOF: time-of-flight
Trp: tryptophan
TSA-ALM: tyramide signal amplification for antibody-overlay lectin microarray
UC: ulcerative colitis
UGM: urinary glycoprofile marker
UHPLC: ultrahigh-performance liquid chromatography
UV: ultraviolet
UVLD: ultraviolet laser desorption
WAX: weak anion exchange
